# Sulfur Ylide-Mediated [2 + 1] Annulation for the Synthesis of Spiro[cyclopropane-indolizine] Derivatives

**DOI:** 10.1021/acsomega.6c03855

**Published:** 2026-07-10

**Authors:** Zheng-Yan Li, Yi-Na Ma, Xing Zheng, Tong Li, Minglong Yuan, Lin Jiang, Mingwei Yuan

**Affiliations:** National and Local Joint Engineering Research Center for Green Preparation Technology of Biobased Materials, School of Chemistry and Environment, 145309 Yunnan Minzu University, Kunming 650504, China

## Abstract

A diastereoselective synthesis of spiro­[cyclo­pro­pane-indo­lizine] derivatives via a [2 + 1] annulation reaction has been developed. This Michael-initiated ring closure of 7-arylmethylidene-6,7-dihydroindolizin-8­(5*H*)-ones with acyl-stabilized sulfur ylides furnishes a diverse array of densely functionalized spirocyclopropanes in moderate to excellent yields (up to 94%) with generally high to excellent diastereoselectivity (up to >20:1 dr). Further derivatization of these tricyclic scaffold-containing products has been accomplished via free-radical C–N bond coupling or sequential cyclopropane ring-opening followed by heteroatom-mediated 1,5-dicarbonyl cyclization.

## Graphical abstract

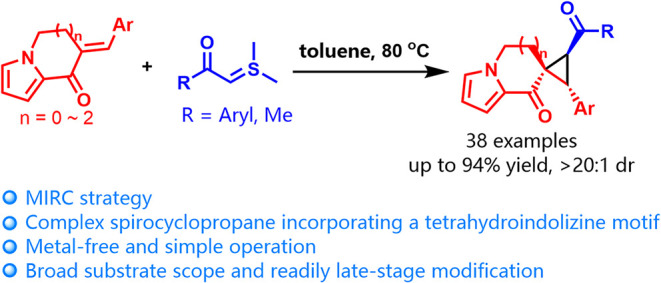

## Introduction

Pyrrole ring is a ubiquitous heteroaromatic scaffold widely present in a broad range of bioactive molecules and natural products, which usually exhibit numerous bioactivities such as antipsychotic, antihypertensive, anticancer, antibacterial effects and enzyme inhibitory properties.^1^ As a distinctive subclass within the structurally complex pyrrole family, 5,6,7,8-tetrahydroindolizines feature an N-fused pyrrole-piperidine core and frequently occur in nature sources such as Polygonatines,^2^ Myrmicarin M215B^3^ and exochomine,^4^ as well as synthetic bioactive molecule like CMV423 ^5^ (Figure 1). To date, 5,6,7,8-tetrahydroindolizines have served as practically useful building blocks for the total synthesis of indolizidine alkaloids bearing varying saturation levels.^6–9^

**Figure 1:**
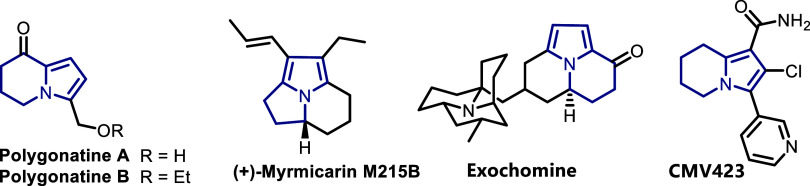
Natural or synthetic bioactive compounds bearing a tetrahydroindolizine core.

Cyclopropane motif represents one of the most popular pharmacophores in marketed small-molecule drugs and potential drug candidates.^10^ Owing to intrinsic ring strain, cyclopropanes often serve as versatile building blocks for the synthesis of diverse functionalized carbocycles and heterocycles through ring-opening and ring-expansion transformations.^11^ Especially, spirocyclopropanes facilitate the construction of high-valued spirocyclic compounds. In drug screening, rational modulation of their unique molecular conformations can improve the metabolic stability and target-binding affinity of candidate molecules.^12^

Given their significant applications in medicinal and synthetic chemistry, the development of efficient synthetic methodologies for spirocyclopropanes has become a topic of growing interest. Several classic strategies, including Simmons–Smith cyclopropanation,^13^ transition metal-catalyzed [2 + 1] cycloaddition via diazo transfer,^14^ Kulinkovich cyclopropanation^15^ and metal-free Michael-initiated ring closure (MIRC) reaction^16^ have been well documented over the past few years. Among these strategies, MIRC reactions between various C1 nucleophiles and activated alkenes have emerged as highly promising approaches. The MIRC strategy allows the application of a wide spectrum of substrates and versatile activation modes, while typically affording satisfying stereocontrol. Stable sulfur ylides are well-known methylene transfer reagents in synthetic organic chemistry and have been widely reported to be involved in cyclization reactions with electron-deficient α,β-unsaturated systems.^17,18^ However, the MIRC transformations of such ylides with enones featuring exocyclic C=C bonds still remains underdeveloped for diversifying spirocyclopropane architectures (Scheme 1, eqn (a-f)).^19–24^

**Scheme 1:**
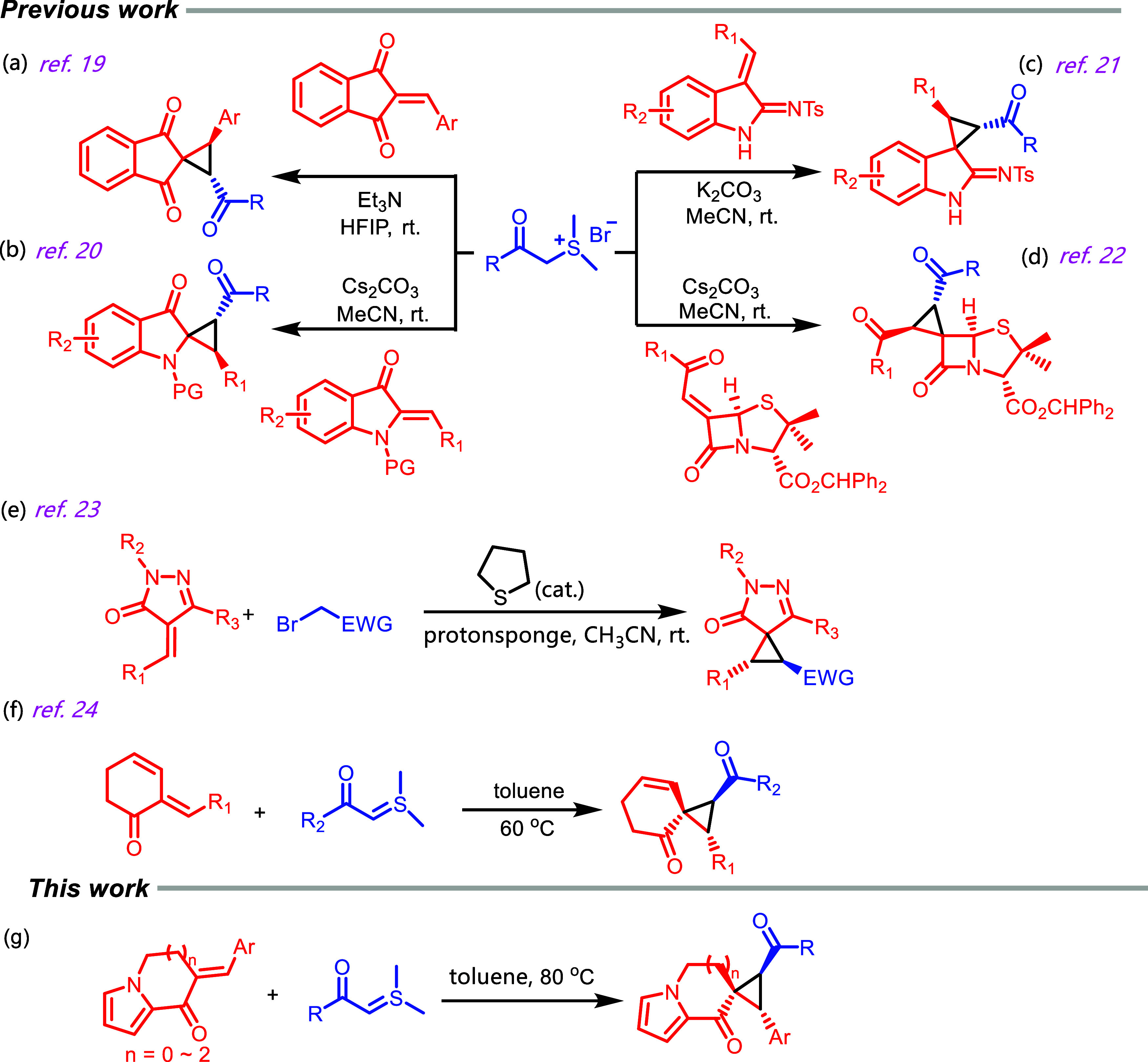
Spirocyclopropanation of Enones Bearing Exocyclic C=C Bonds with Sulfur Ylides (Previous Work and This Work)

In our earlier study on the sulfur ylide-mediated spirocyclopropanation of exocyclic enones, we confirmed that the extended branched π-conjugated structure of enones was beneficial for effective stabilization of the carbanion intermediates formed *via* Michael addition, thereby facilitating the subsequent cyclization (Scheme 1, eqn (f)).^24^ As part of our ongoing efforts to develop novel synthetic methodologies for structural diversification of spirocyclopropanes, we envisaged that 7-arylmethylidene-6,7-dihydroindolizin-8­(5*H*)-ones, a class of nitrogen-containing fused-ring exocyclic enones, could offer a favorable electron delocalization pathway to address the common issue of unstable intermediates typically encountered in the spirocyclopropanation of exocyclic enones *via* MIRC approaches. Herein, we present a diastereoselective synthesis of spiro­[cyclopropane-indolizine] derivatives *via* a [2 + 1] annulation of 7-arylmethylidene-6,7-dihydroindolizin-8­(5*H*)-ones with acyl-stabilized sulfur ylides (Scheme 1, eqn (g)). Given the importance of integrating two privileged pharmacophores into a single scaffold in drug discovery, the resulting spirocycles may hold promise for medicinal applications.

## Results and Discussion

We began our investigation by testing the feasibility of the proposed spirocyclopropanation process, and readily preparable (*E*)-7-benzylidene-6,7-dihydroindolizin-8­(5*H*)-one **1a** and sulfur ylide bearing *p*-methoxybenzoyl **2a** were selected as model substrates in DCE (Table 1, entry 1). Gratifyingly, the anticipated [2 + 1] annulation took place efficiently at a 1:1.5 molar ratio of **1a** and **2a** at 80 °C, and the spirocyclopropane **3aa** was preferentially produced with 2,3-*trans* selectivity in 74% isolated yield with 6.7:1 dr. Encouraged by this result, we continued to study the solvent effect either at 80 °C or at reflux temperature (entries 2–8). The reaction was found to tolerate various solvents. Among them, toluene proved to be the optimal choice by improving the yield of **3aa** to 82%. Subsequently, a brief investigation into the molar ratio of **1a** and **2a** was carried out. To our delight, when the loading of **2a** was increased to 2.0 equiv, **3aa** was obtained in 86% yield with the same diastereoselectivity. In comparison, decreasing the loading of **2a** led to an obvious drop in yield (entries 9 and 10). Further variation of the reaction temperature or substrate concentration did not enhance the outcome, instead, diminished yields and sluggish reaction rates were observed in most cases (entries 11–14). Besides, performing the reaction under an argon atmosphere exerted little effect on further improving the reaction yield (entry 15). After establishing the optimal reaction conditions (entry 9), we further explored a catalytic version of this transformation by reacting **1a** with *p*-methoxyphenacyl bromide (2.0 equiv) and Cs_2_CO_3_ (2.0 equiv) in the presence of tetrahydrothiophene as a catalyst (20 mol %). However, only trace **3aa** was detected at 80 °C alongside full consumption of the bromide.

**Table 1:**
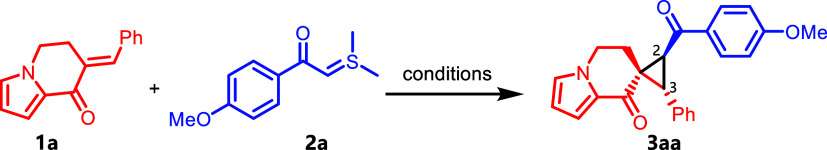
Optimization of the Reaction Conditions^a^

entry	solvent	time (h)	*T* (°C)	ratio **1a**/**2a**	Dr^b^	yield (%)^c^
1	DCE	24	80	1:1.5	6.7:1	74
2	toluene	44	80	1:1.5	10:1	82
3	MeCN	24	80	1:1.5	10:1	78
4	PhCF_3_	44	80	1:1.5	10:1	78
5	PhF	44	80	1:1.5	10:1	80
6	EtOH	44	reflux	1:1.5	10:1	27
7	DMF	24	80	1:1.5	10:1	25
8	THF	44	reflux	1:1.5	20:1	19
**9**	**toluene**	**44**	**80**	1:2.0	10:1	**86**
10	toluene	44	80	1:1.2	10:1	75
11	toluene	70	70	1:2.0	20:1	70
12	toluene	44	90	1:2.0	6.7:1	74
13^d^	toluene	44	80	1:2.0	6.7:1	82
14^e^	toluene	66	80	1:2.0	10:1	77
15^f^	toluene	44	80	1:2.0	10:1	87

a Unless otherwise noted, the reactions were carried out on a 0.2 mmol of **1a** in 2.0 mL of solvent.

b Dr was determined by ^1^H NMR analysis of the crude reaction mixtures.

c Isolated yield.

d 1.0 mL of toluene.

e 4.0 mL of toluene.

f Under an Ar atmosphere.

With acceptable optimized conditions in hand, we next evaluated the reaction scope of (*E*)-7-arylidene-6,7-dihydroindolizin-8­(5*H*)-one **1** using sulfur ylide **2a** as a cyclization partner. As presented in Table 2, enones **1** with various aryl groups (Ar) were well tolerated, giving access to the corresponding spiro­[cyclopropane-indolizine] derivatives **3** with good to excellent yields and diastereoselectivity within 44 h. The Ar group could be a phenyl ring that bears different electronic and steric substituents (**3aa-3pa**). In general, superior yields were achieved for enones **1** with an electron-withdrawing group (i.e., halogen, −CN, -NO_2_) on the phenyl ring, as opposed to those appended with an electron-donating group (i.e., -OMe, -Me, -Ph). The position of substituents (R) on the phenyl rings exerts an obvious influence on diastereoselectivity, as *ortho*-substituted enones exhibit lower diastereoselectivity than their *meta*- or *para*-substituted counterparts. As a structural analogue of the phenyl-containing **1a**, the sterically more hindered 2-naphthyl-substituted enone also underwent the reaction smoothly, giving product **3qa** in 81% yield with 10:1 dr. Moreover, enones incorporating heteroaryl moieties, such as 2-furyl and 2-thienyl groups, were also compatible with this annulation, albeit affording products **3ra** and **3sa** in moderate yields. In addition, substrates **1** bearing five- or seven-membered cyclic ketone moiety could also be converted into the desired products, though the yield of the seven-membered product **3ua** was much lower than the five-membered counterparts **3ta**.

**Table 2:**
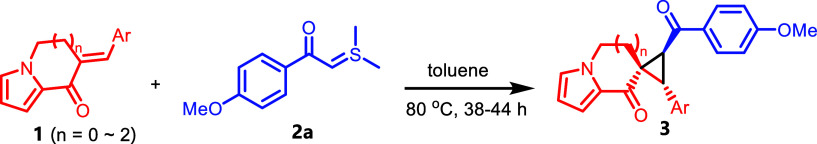

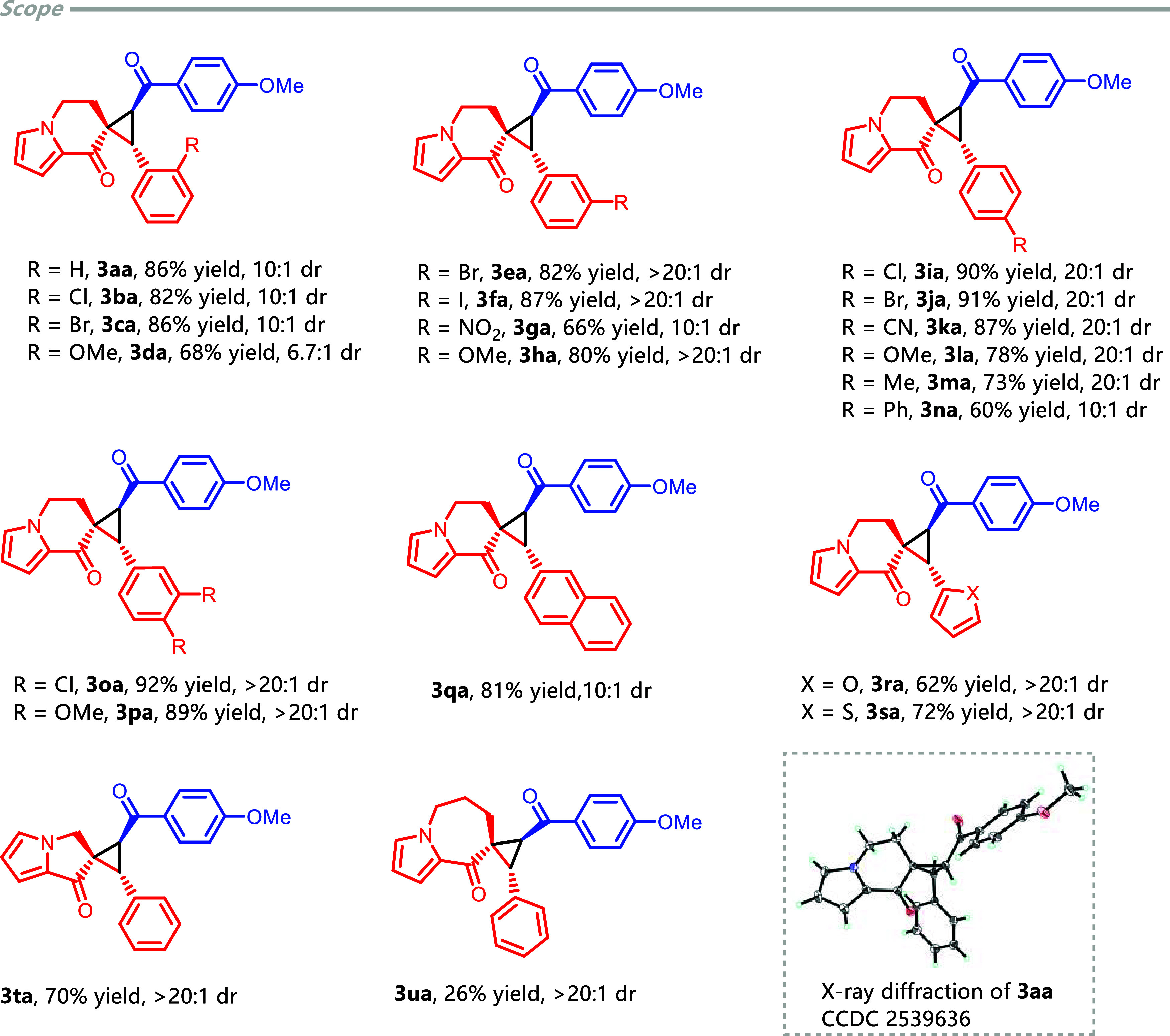
Substrate Scope of (*E*)-7-Arylidene-6,7-dihydroindolizin-8­(5*H*)-ones **1** ^a^ ^,^ ^b^

a All reactions were carried out using **1** (0.2 mmol) and **2a** (0.4 mmol) in toluene (2.0 mL) at 80 °C.

b Isolated yield; dr was determined by ^1^H NMR analysis of the crude reaction mixtures.

The structure of the product **3aa** was unambiguously confirmed by X-ray crystallographic analysis (CCDC 2539636)^25^ and other products were assigned by analogy.^26^

To further expand the substrate scope, we then turned our attention to exploring the functional group tolerance of other stable sulfur ylides **2**. During our exploration, we found that in many cases the products overlapped in polarity with phenyl-substituted substrate **1a**, thus causing difficulties in TLC monitoring and isolation. Therefore, 3,4-dichloro- and 4-methoxy-substituted enones (**1o** and **1l**) were employed in place of **1a** to react with sulfur ylides. As summarized in Table 3, sulfur ylides **2** bearing halogen or electron-donating substituents at different positions on the phenyl ring exhibited good functional group tolerance, generating the corresponding products in moderate to excellent yields with generally high diastereoselectivity. Nevertheless, substrates bearing a strong electron-withdrawing group (i.e., -NO_2_ or −CN) on the phenyl ring exerted a deleterious effect on the product yields under standard conditions (**3og** and **3oj**). This outcome can be rationalized by the significantly reduced nucleophilicity of sulfur ylides induced by strongly electron-withdrawing acyl substituents. Furthermore, variation of R_2_ by 2-naphthyl and 2-thienyl substituents was feasible, affording products **3on** and **3oo** in 86% and 80% yields, respectively. Notably, the acetyl-substituted sulfur ylide was amenable to this annulation reaction, furnishing product **3op** as a single diastereomer in 86% yield.

**Table 3:**
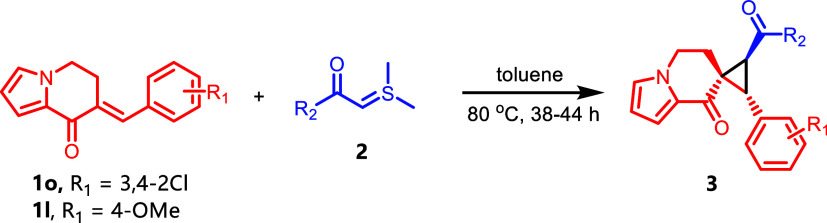

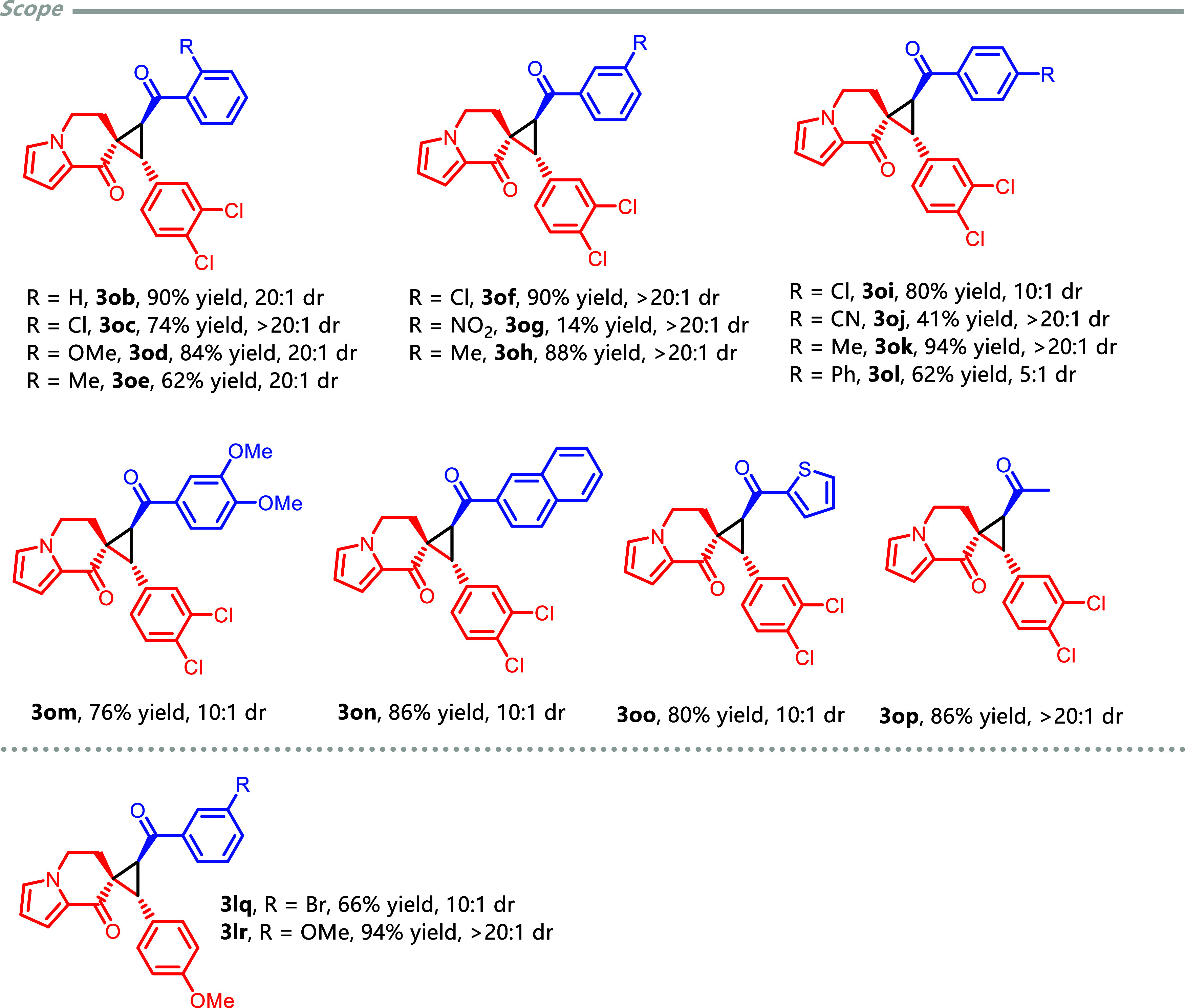
Substrate Scope of Sulfur Ylides **2** ^a^,^b^

a All reactions were carried out using **1o** or **1l** (0.2 mmol) and **2** (0.4 mmol) in toluene (2.0 mL) at 80 °C.

b Isolated yield; dr was determined by ^1^H NMR analysis of the crude reaction mixtures.

To demonstrate the preparative utility of this protocol, a gram-scale reaction was carried out (Scheme 2, eqn (a)). Pleasingly, the reaction of 1.0 g of **1a** with **2a** proceeded efficiently under the established reaction conditions, furnishing product **3aa** without an apparent loss in either yield or diastereoselectivity. The spirocyclic product **3aa** could be easily transformed into versatile derivatives. For example, reductive ring-opening of the cyclopropane skeleton mediated by zinc powder and zinc chloride delivered a pair of isomers **4** with poor diastereoselectivity. Treatment of the diastereoisomeric mixture of ring-opening intermediates **4** with an excess amount of ammonium acetate in glacial acetic acid triggered a [5 + 1] heterocyclization, furnishing heterocycle **5** bearing a valuable pyrrole-fused naphthyridine scaffold (eqn (b)). Furthermore, the coupling reaction of **3aa** with 1*H*-benzotriazole in the presence of Selectfluor delivered product **6** *via* a single-electron transfer pathway (eqn (c)).

**Scheme 2:**
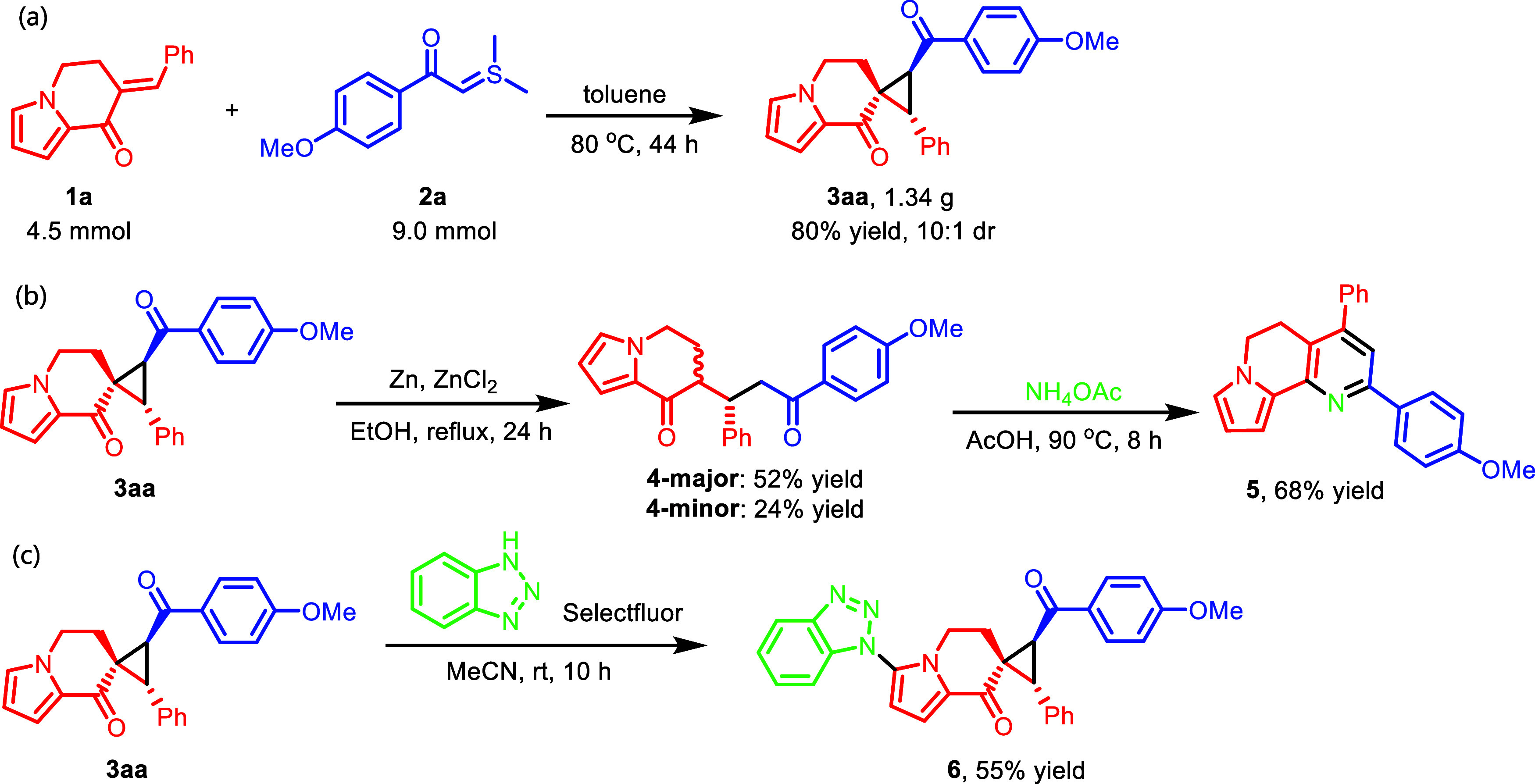
Gram-Scale Experiment and Synthetic Transformations of Product **3aa**

Based on the experimental results and previous literature reports,^20,22^ a plausible reaction mechanism accounting for the MIRC approach is proposed as outlined in Scheme 3. First, the reaction is triggered by nucleophilic attack of sulfur ylide **2a** on enone **1a**. Zwitterionic intermediates then form predominantly as the more stable 2,3-*trans*-configured species **Int. I**, which is favored over the 2,3-*cis* analogue **Int. II** due to reduced steric hindrance. **Int. I** further undergoes intramolecular S_N_2 cyclization with concomitant extrusion of dimethyl sulfide, furnishing the major 2,3-*trans* isomer **3aa**.

**Scheme 3:**
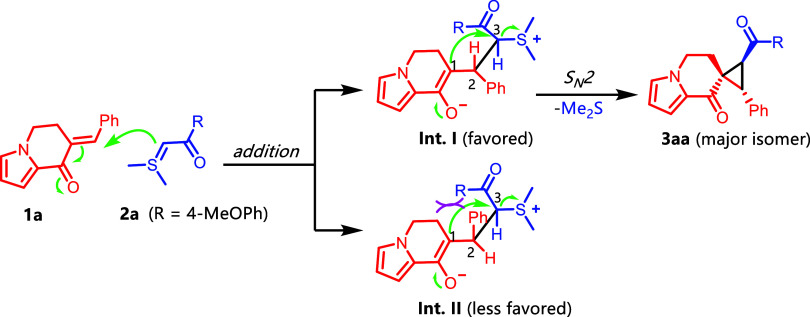
Plausible Reaction Mechanism

## Conclusion

In conclusion, we have developed a practical method for the diastereoselective construction of spiro­[cyclopropane-indolizine] derivatives through the MIRC reaction. Within this [2 + 1] annulation, both (*E*)-7-arylidene-6,7-dihydroindolizin-8­(5*H*)-ones and acyl-stabilized sulfur ylides show good functionality tolerance of the standard reaction conditions in most cases, giving access to target products in moderate to excellent yields with high to excellent diastereoselectivity. This synthetic protocol can be readily scaled up. Derivatization studies demonstrate that a pyrrole-fused naphthyridine framework could be constructed via sequential cyclopropane ring-opening followed by heteroatom-mediated [5 + 1] cyclization. Moreover, the 1*H*-benzotriazole moiety could be introduced to the resulting spirocyclopropane products. We anticipate that this protocol will offer a new entry for expanding the library of complex spirocyclopropane molecules, and the intriguing structural features of the products will draw attention from synthetic chemists and pharmacologists in the near future.

## Supplementary Material



## Data Availability

The data underlying this study are available in the published article and its Supporting Information.

## Notes

General information, synthetic procedures, characterization data and copies of NMR spectra for compounds **3**–**6**, X-ray crystal structure of compound **3aa** (PDF)
