# Supplementary material for: Sulfur Ylide-Mediated [2 + 1] Annulation for the Synthesis of Spiro[cyclopropane-indolizine] Derivatives
Source: ACS Omega. 2026 Jul 10;11(28):42673–80. doi: 10.1021/acsomega.6c03855 (PMC13393377; doi:10.1021/acsomega.6c03855)
Supplement: Supplementary file 1 [file ao6c03855_si_001.pdf]

# SUPPORTING INFORMATION

## Sulfur Ylide-Mediated [2+1] Annulation for the Synthesis of Spiro[cyclopropane-indolizine] Derivatives

Zheng-Yan Li, Yi-Na Ma, Xing Zheng, Tong Li, Minglong Yuan, Lin Jiang* and Mingwei Yuan*

*National and Local Joint Engineering Research Center for Green Preparation Technology of Biobased Materials, School of Chemistry and Environment, Yunnan Minzu University, Kunming 650504, P. R. China*

# Table of Contents

I. General information....................................................................................................... 1

II. Synthetic procedures.....................................................................................................1-3

1) General procedure for the synthesis of products **3** ................................................................. 1
2) Gram-scale synthesis of product **3aa** ......................................................................1-2
3) Synthetic procedure for preparing compound **4** ................................................................ 2
4) Synthetic procedure for preparing compound **5** ................................................................ 2
5) Synthetic procedure for preparing compound **6** ..............................................................2-3

III. Characterization of all the newly synthesized compounds **3-6** ..........................................3-21

IV. References...................................................................................................................21-22

V. Copies of NMR spectra..................................................................................................23-65

VI. X-ray crystallographic data of product **3aa** ...............................................................66-67

# I. General information

NMR spectra were recorded on a Bruker ADVANCE III instrument at 600 MHz ($^{1}$H NMR) and 150 MHz ($^{13}$C NMR), or at 400 MHz ($^{1}$H NMR) and 100 MHz ($^{13}$C NMR) with chloroform-*d* as the solvent. The chemical shifts (δ) were expressed in parts per million (ppm) and the coupling constants (*J*) were in Hz. $^{1}$H NMR spectra was reported using tetramethylsilane (TMS) as an internal standard (TMS at 0.00 ppm) and $^{13}$C NMR spectra using solvent as an internal standard ($CDCl_3$ at 77.16 ppm). HRMS (ESI) were recorded on a Waters SYNAPT G2 mass spectrometer. X-ray diffraction experiments were carried out on an Agilent Gemini and the data obtained were deposited at the Cambridge Crystallographic Data Centre. Reactions were monitored by thin-layer chromatography (TLC) with pre-coated G254 silica gel. Column chromatography was carried out on silica gel (200−300 mesh). Unless otherwise noted, all solvents and chemical reagents were used as commercially available without further purification. All melting points were measured with the samples after column chromatography and uncorrected. Compounds (*E*)-7-arylidene-6,7-dihydroindolizin-8(5*H*)-ones **1**[1,2] and sulfur ylides **2**[3], used as starting materials, were prepared following the literature procedures.

# II. Synthetic procedures

1). General procedure for the synthesis of products **3**

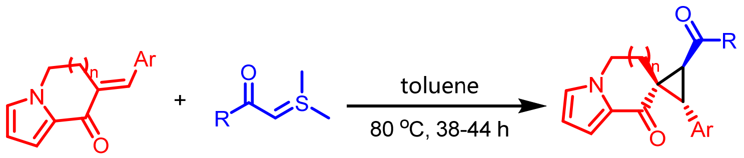


**1** **2** **3**

A solution of **1** (0.20 mmol) and acyl-stabilized sulfur ylide **2** (0.40 mmol) was stirred in dry toluene (2 mL) at 80 °C. Upon completion of the reaction as monitored by TLC, the reaction mixture was cooled to room temperature and then purified by flash chromatography over silica gel (eluent: petroleum ether/ethyl acetate = 5:1 (v/v)) to provide the product **3**.

2). Gram-scale synthesis of product **3aa**

The reaction was performed on a gram scale according to the general procedure, with only adjusting the reaction scales. A mixture of (*E*)-7-benzylidene-6,7-dihydroindolizin-8(5*H*)-one **1a** (1.00 g, 4.5

mmol) and sulfur ylide **2a** (1.89 g, 9.0 mmol) in toluene (45 mL) was stirred at 80 °C for 44 h. After completion, the reaction mixture was cooled to room temperature and then purified by flash chromatography over silica gel (eluent: petroleum ether/ethyl acetate = 5:1 (v/v)) to provide the product **3aa** in 80% yield (1.34 g) with 10:1 dr.

3). Synthetic procedure for preparing compound **4**

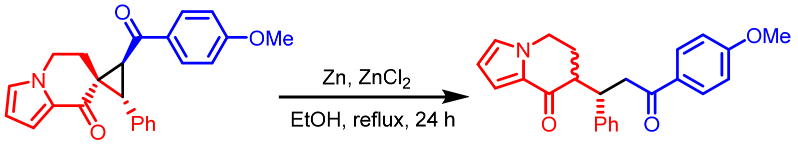


**3aa** **4** (major and minor)

A mixture of product **3aa** (37.1 mg, 0.10 mmol), Zn (32.7 mg, 0.50 mmol) and $ZnCl_2$ (68.2 mg, 0.50 mmol) was stirred in EtOH (2 mL) at reflux for 24 h. After completion, the reaction mixture was cooled to room temperature and filtered through celite. The filtrate was concentrated under reduced pressure, and the residue was purified by flash chromatography over silica gel (eluent: petroleum ether/ethyl acetate = 5:1 (v/v)) to afford the compounds **4-major** and **4-minor**.

4). Synthetic procedure for preparing compound **5**

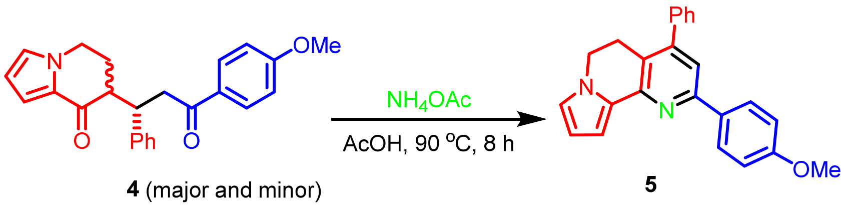


**4** (major and minor) **5**

A mixture of compound **4** (major and minor, 65.5 mg, 0.18 mmol) and $NH_4OAc$ (111.0 mg, 1.44 mmol) was stirred in AcOH (2 mL) at 90 °C for 8 h. After completion, the reaction mixture was cooled to room temperature, diluted with ethyl acetate (10 mL), and washed with water (5 mL) three times. Then the organic layer was successively washed with saturated brine, dried over anhydrous $Na_2SO_4$, and concentrated under reduced pressure. The crude is purified by flash chromatography over silica gel (eluent: petroleum ether/ethyl acetate = 8:1 (v/v)) to provide the compound **5**.

5). Synthetic procedure for preparing compound **6**

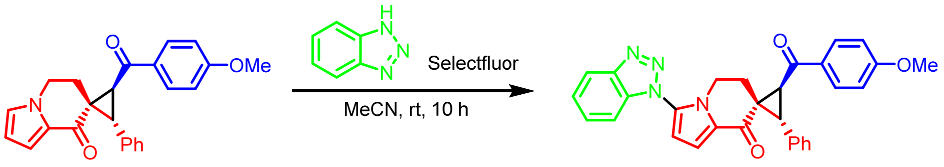


**3aa** **6**

A mixture of product **3aa** (37.1 mg, 0.10 mmol), 1*H*-benzotriazole (17.9 mg, 0.15 mmol) and Selectfluor (46.1 mg, 0.13 mmol) was stirred in MeCN (1 mL) at rt for 10 h. After completion, the reaction mixture was dispersed in $CH_2Cl_2$ (10 mL), and then washed with water (2 mL) three times. Then the organic layer was successively washed with saturated brine, dried over anhydrous $Na_2SO_4$, and concentrated under reduced pressure. The crude is purified was purified by flash chromatography over silica gel (eluent: petroleum ether/ethyl acetate = 4:1 (v/v)) to give the compound **6**.

## III. Characterization of all the newly synthesized compounds 3-6

### (1S,2S,3R)-2-(4-Methoxybenzoyl)-3-phenyl-5',6'-dihydro-8'*H*-spiro[cyclopropane-1,7'-indolizin]-8'-one (3aa)

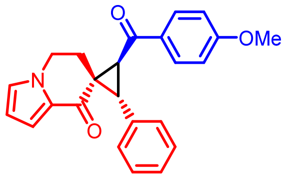


Yellow solid, 63.9 mg, 86% yield, m.p. 126-128 °C. $^1$H NMR (400 MHz, $CDCl_3$) δ 8.10 (d, $J$ = 8.0 Hz, 2H), 7.21 (d, $J$ = 4.0 Hz, 2H), 7.17-7.14 (m, 3H), 6.97 (d, $J$ = 8.0 Hz, 2H), 6.91 (d, $J$ = 4.0 Hz, 1H), 6.87-6.86 (m, 1H), 6.23 (d, $J$ = 8.0 Hz, 1H), 4.50 (d, $J$ = 8.0 Hz, 1H), 4.41-4.27 (m, 2H), 3.88 (s, 3H), 3.52 (d, $J$ = 8.0 Hz, 1H), 2.35 (td, $J$ = 12.0 Hz, 8.0 Hz, 1H), 2.24 (d, $J$ = 16.0 Hz, 1H). $^{13}$C NMR (100 MHz, $CDCl_3$) δ 194.6, 181.3, 164.1, 134.7, 131.5, 131.1, 130.8, 129.3, 128.3, 127.2, 126.1, 114.9, 114.1, 110.8, 55.7, 45.4, 43.3, 39.0, 32.8, 28.5. HRMS (ESI) calcd. for $C_{24}H_{22}NO_3$ $[M+H]^+$ 372.1600, found 372.1604.

### (1S,2S,3S)-2-(4-Methoxybenzoyl)-3-phenyl-5',6'-dihydro-8'*H*-spiro[cyclopropane-1,7'-indolizin]-8'-one (3aa′)

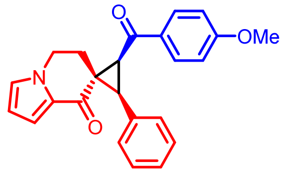


As a separable diastereoisomer of **3aa**, **3aa′** was prepared on a 1.0 mmol scale of **1a**: Yellow oil. $^1$H

NMR (400 MHz, $CDCl_3$) δ 8.04 (d, $J$ = 8.0 Hz, 2H), 7.25-7.19 (m, 3H), 7.10-7.07 (m, 3H), 6.95 (d, $J$ = 8.0 Hz, 2H), 6.87 (d, $J$ = 4.0 Hz, 1H), 6.30 (d, $J$ = 4.0 Hz, 1H), 4.15-4.01 (m, 2H), 3.87 (s, 3H), 3.77 (d, $J$ = 8.0 Hz, 1H), 3.54 (d, $J$ = 8.0 Hz, 1H), 2.79-2.73 (m, 1H), 2.55-2.48 (m, 1H). $^{13}C$ NMR (100 MHz, $CDCl_3$) δ 194.6, 185.7, 163.7, 133.8, 131.9, 130.6, 130.3, 130.0, 128.3, 127.0, 126.5, 114.8, 113.9, 110.8, 55.6, 44.8, 38.0, 36.9, 34.4, 23.8. HRMS (ESI) calcd. for $C_{24}H_{22}NO_3$ $[M+H]^+$ 372.1600, found 372.1602.

**(1S,2S,3S)-2-(2-Chlorophenyl)-3-(4-methoxybenzoyl)-5',6'-dihydro-8'*H*-spiro[cyclopropane-1, 7'-indolizin]-8'-one (3ba)**

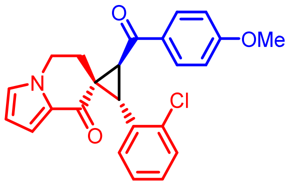


Yellow oil, 66.6 mg, 82% yield. $^1H$ NMR (600 MHz, $CDCl_3$) δ 8.08 (d, $J$ = 6.0 Hz, 2H), 7.28 (d, $J$ = 6.0 Hz, 1H), 7.24 (d, $J$ = 6.0 Hz, 1H), 7.20-7.18 (m, 1H), 7.15 (d, $J$ = 12.0 Hz, 1H), 6.96 (d, $J$ = 12.0 Hz, 2H), 6.91 (d, $J$ = 6.0 Hz, 1H), 6.85 (s, 1H), 6.22 (d, $J$ = 6.0 Hz, 1H), 4.63-4.59 (m, 1H), 4.45 (d, $J$ = 12.0 Hz, 1H), 4.19 (dt, $J$ = 12.0 Hz, 6.0 Hz), 3.87 (s, 3H), 3.55 (d, $J$ = 6.0 Hz, 1H), 2.36-2.34 (m, 2H). $^{13}C$ NMR (150 MHz, $CDCl_3$) δ 194.1, 180.9, 164.1, 135.2, 132.6, 131.2, 131.0, 130.8, 130.7, 129.1, 128.7, 126.4, 126.3, 114.8, 114.0, 110.7, 55.6, 44.7, 42.2, 37.5, 33.0, 28.1. HRMS (ESI) calcd. for $C_{24}H_{20}ClNNaO_3$ $[M+Na]^+$ 428.1029, found 428.1034.

**(1S,2S,3S)-2-(2-Bromophenyl)-3-(4-methoxybenzoyl)-5',6'-dihydro-8'*H*-spiro[cyclopropane-1, 7'-indolizin]-8'-one (3ca)**

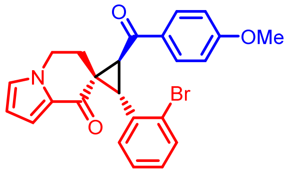


Yellow solid, 77.5 mg, 86% yield, m.p. 133-135 °C. $^1H$ NMR (600 MHz, $CDCl_3$) δ 7.98 (d, $J$ = 6.0 Hz, 2H), 7.35 (d, $J$ = 6.0 Hz, 1H), 7.18 (d, $J$ = 6.0 Hz, 1H), 7.15-7.13 (m, 1H), 6.99-6.96 (m, 1H), 6.86 (d, $J$ = 6.0 Hz, 2H), 6.83 (d, $J$ = 6.0 Hz, 1H), 6.76 (s, 1H), 6.13 (d, $J$ = 6.0 Hz, 1H), 4.58 (dt, $J$ = 12.0 Hz, 6.0 Hz), 4.33 (d, $J$ = 6.0 Hz, 1H), 4.08 (dt, $J$ = 12.0 Hz, 6 Hz, 1H), 3.77 (s, 3H), 3.43 (d, $J$ = 12.0 Hz, 1H), 2.32-2.22 (m, 2H). $^{13}C$ NMR (150 MHz, $CDCl_3$) δ 194.0, 181.0, 164.0, 134.2, 132.4,

131.3, 131.0, 130.9, 130.7, 128.9, 127.0, 126.3, 125.8, 114.8, 114.0, 110.7, 55.6, 44.8, 42.3, 40.0, 33.7, 28.0. HRMS (ESI) calcd. for $C_{24}H_{21}BrNO_3$ $[M+H]^+$ 450.0705, found 450.0708.

**(1S,2S,3R)-2-(4-Methoxybenzoyl)-3-(2-methoxyphenyl)-5',6'-dihydro-8'*H*-spiro[cyclopropane-1,7'-indolizin]-8'-one (3da)**

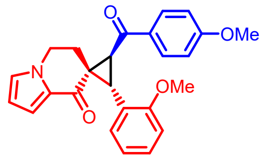


Yellow solid, 54.6 mg, 68% yield, m.p. 178-180 °C. $^1H$ NMR (600 MHz, $CDCl_3$) δ 8.09 (d, $J$ = 12.0 Hz, 2H), 7.20-7.15 (m, 2H), 6.96 (d, $J$ = 6.0 Hz, 2H), 6.89-6.87 (m, 3H), 6.67 (d, $J$ = 6.0 Hz, 1H), 6.21 (d, $J$ = 6.0 Hz, 1H), 4.62 (td, $J$ = 12.0 Hz, 6.0 Hz, 1H), 4.36 (d, $J$ = 6.0 Hz, 1H), 4.25-4.22 (m, 1H), 3.87 (s, 3H), 3.38 (d, $J$ = 12.0 Hz, 1H), 3.34 (s, 3H), 2.34 (td, $J$ = 12.0 Hz, 6.0 Hz, 1H), 2.24 (d, $J$ = 18.0 Hz, 1H). $^{13}C$ NMR (150 MHz, $CDCl_3$) δ 195.1, 181.6, 163.9, 158.1, 131.4, 131.2, 130.9, 130.5, 128.6, 125.5, 123.3, 120.3, 114.0, 113.9, 110.3, 109.9, 55.6, 54.4, 44.6, 41.9, 34.7, 32.5, 28.2. HRMS (ESI) calcd. for $C_{25}H_{24}NO_4$ $[M+H]^+$ 402.1705, found 402.1703.

**(1S,2R,3S)-2-(3-Bromophenyl)-3-(4-methoxybenzoyl)-5',6'-dihydro-8'*H*-spiro[cyclopropane-1,7'-indolizin]-8'-one (3ea)**

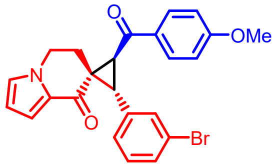


Yellow solid, 73.8 mg, 82% yield, m.p. 156-158 °C. $^1H$ NMR (600 MHz, $CDCl_3$) δ 8.07 (d, $J$ = 6.0 Hz, 2H), 7.34 (s, 1H), 7.30-7.28 (m, 1H), 7.07 (d, $J$ = 6.0 Hz, 2H), 6.96 (d, $J$ = 12.0 Hz, 2H), 6.94 (d, $J$ = 6.0 Hz, 1H), 6.88 (s, 1H), 6.24 (d, $J$ = 6.0 Hz, 1H), 4.46 (d, $J$ = 6.0 Hz, 1H), 4.36-4.26 (m, 2H), 3.87 (s, 3H), 3.46 (d, $J$ = 6.0 Hz, 1H), 2.33 (td, $J$ = 18.0 Hz, 6.0 Hz, 1H), 2.20 (d, $J$ = 12.0 Hz, 1H). $^{13}C$ NMR (150 MHz, $CDCl_3$) δ 194.1, 180.8, 164.1, 137.1, 132.4, 131.3, 131.0, 130.6, 130.3, 129.7, 127.8, 126.4, 122.3, 115.1, 114.1, 110.9, 55.7, 45.2, 43.2, 38.0, 32.8, 28.3. HRMS (ESI) calcd. for $C_{24}H_{21}BrNO_3$ $[M+H]^+$ 450.0705, found 450.0708.

**(1S,2R,3S)-2-(3-Iodophenyl)-3-(4-methoxybenzoyl)-5',6'-dihydro-8'*H*-spiro[cyclopropane-1,7'-indolizin]-8'-one (3fa)**

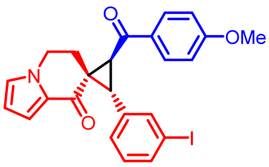


Yellow solid, 86.5 mg, 87% yield, m.p. 100-102 ºC. $^{1}$H NMR (600 MHz, $CDCl_3$) δ 8.07 (d, $J$ = 12.0 Hz, 2H), 7.54 (s, 1H), 7.48 (d, $J$ = 12.0 Hz, 1H), 7.10 (d, $J$ = 6.0 Hz, 1H), 6.96-6.92 (m, 4H), 6.88 (s, 1H), 6.24 (d, $J$ = 6.0 Hz, 1H), 4.44 (d, $J$ = 6.0 Hz, 1H), 4.35-4.26 (m, 2H), 3.86 (s, 3H), 3.44 (d, $J$ = 6.0 Hz, 1H), 2.32 (td, $J$ = 12.0 Hz, 6.0 Hz, 1H), 2.18 (d, $J$ = 12.0 Hz, 1H). $^{13}$C NMR (150 MHz, $CDCl_3$) δ 194.0, 180.8, 164.1, 138.3, 137.2, 136.2, 131.2, 131.0, 130.5, 129.9, 128.4, 126.4, 1151, 114.0, 110.9, 94.2, 55.6, 45.2, 43.1, 37.8, 32.7, 28.2. HRMS (ESI) calcd. for $C_{24}H_{20}INNaO_3$ $[M+Na]^+$ 520.0386, found 520.0390.

**(1S,2S,3R)-2-(4-Methoxybenzoyl)-3-(3-nitrophenyl)-5',6'-dihydro-8'*H*-spiro[cyclopropane-1,7'-indolizin]-8'-one (3ga)**

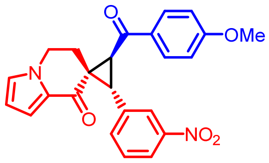


Yellow solid, 55.0 mg, 66% yield, m.p. 215-217 ºC. $^{1}$H NMR (600 MHz, $CDCl_3$) δ 8.07 (d, $J$ = 6.0 Hz, 2H), 8.06 (s, 1H), 8.02 (d, $J$ = 12.0 Hz, 1H), 7.50 (d, $J$ = 6.0 Hz, 1H), 7.40-7.37 (m, 1H), 6.97 (d, $J$ = 6.0 Hz, 2H), 6.91-6.90 (m, 2H), 6.23 (d, $J$ = 6.0 Hz, 1H), 4.52 (d, $J$ = 6.0 Hz, 1H), 4.38-4.29 (m, 2H), 3.88 (s, 3H), 3.57 (d, $J$ = 6.0 Hz, 1H), 2.37 (td, $J$ = 12.0 Hz, 6.0 Hz, 1H), 2.23 (d, $J$ = 12.0 Hz, 1H). $^{13}$C NMR (150 MHz, $CDCl_3$) δ 193.6, 180.6, 164.3, 148.1, 136.9, 135.4, 131.1, 131.1, 130.4, 129.1, 126.8, 124.4, 122.3, 115.4, 114.2, 111.1, 55.7, 45.1, 43.1, 37.5, 33.0, 28.1. HRMS (ESI) calcd. for $C_{24}H_{21}N_2O_5$ $[M+H]^+$ 417.1450, found 417.1454.

**(1S,2S,3R)-2-(4-Methoxybenzoyl)-3-(3-methoxyphenyl)-5',6'-dihydro-8'*H*-spiro[cyclopropane-1,7'-indolizin]-8'-one (3ha)**

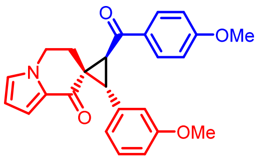


Yellow solid, 64.2 mg, 80% yield, m.p. 143-145 ºC. $^{1}$H NMR (600 MHz, $CDCl_3$) δ 8.09 (d, $J$ = 12.0 Hz, 2H), 7.13-7.11 (m, 1H), 6.96 (d, $J$ = 6.0 Hz, 2H), 6.93 (d, $J$ = 6.0 Hz, 1H), 6.86 (s, 1H), 6.75 (d, $J$ = 12.0 Hz, 1H), 6.71 (s, 1H), 6.70 (d, $J$ = 6.0 Hz, 1H), 6.22 (d, $J$ = 6.0 Hz, 1H), 4.49 (d, $J$ = 6.0 Hz,

1H), 4.36 (td, $J$ = 12.0 Hz, 6.0 Hz, 1H), 4.30-4.26 (m, 1H), 3.86 (s, 3H), 3.70 (s, 3H), 3.49 (d, $J$ = 6.0 Hz, 1H), 2.33 (td, $J$ = 12.0 Hz, 6.0 Hz, 1H), 2.20 (d, $J$ = 12.0 Hz, 1H). $^{13}$C NMR (150 MHz, $CDCl_3$) δ 194.5, 181.2, 164.0, 159.3, 136.3, 131.4, 131.0, 130.7, 129.2, 126.1, 121.6, 115.1, 114.8, 114.0, 112.4, 110.7, 55.6, 55.1, 45.3, 43.2, 38.7, 32.8, 28.3. HRMS (ESI) calcd. for $C_{25}H_{24}NO_4$ $[M+H]^+$ 402.1705, found 402.1708.

**(1S,2R,3S)-2-(4-Chlorophenyl)-3-(4-methoxybenzoyl)-5',6'-dihydro-8'*H*-spiro[cyclopropane-1,7'-indolizin]-8'-one (3ia)**

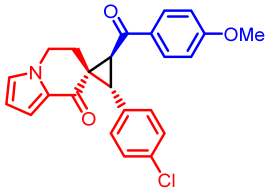


Yellow solid, 73.1 mg, 90% yield, m.p. 174-176 ºC. $^1$H NMR (600 MHz, $CDCl_3$) δ 8.07 (d, $J$ = 6.0 Hz, 2H), 7.17 (d, $J$ = 6.0 Hz, 2H), 7.10 (d, $J$ = 6.0 Hz, 2H), 6.96 (d, $J$ = 6.0 Hz, 2H), 6.92 (d, $J$ = 6.0 Hz, 1H), 6.87 (s, 1H), 6.23 (d, $J$ = 6.0 Hz, 1H), 4.46 (d, $J$ = 6.0 Hz, 1H), 4.36-4.26 (m, 2H), 3.87 (s, 3H), 3.46 (d, $J$ = 6.1 Hz, 1H), 2.36-2.31 (m, 1H), 2.20 (d, $J$ = 18.0 Hz, 1H). $^{13}$C NMR (150 MHz, $CDCl_3$) δ 194.2, 181.0, 164.1, 133.2, 132.9, 131.3, 131.0, 130.6, 130.6, 128.4, 126.4, 115.0, 114.2, 110.9, 55.6, 45.2, 43.1, 38.0, 32.8, 28.2. HRMS (ESI) calcd. for $C_{24}H_{20}ClNNaO_3$ $[M+Na]^+$ 428.1029, found 428.1025.

**(1S,2R,3S)-2-(4-Bromophenyl)-3-(4-methoxybenzoyl)-5',6'-dihydro-8'*H*-spiro[cyclopropane-1,7'-indolizin]-8'-one (3ja)**

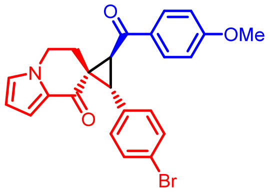


Yellow solid, 82.0 mg, 91% yield, m.p. 140-142 ºC. $^1$H NMR (600 MHz, $CDCl_3$) δ 8.07 (d, $J$ = 12.0 Hz, 2H), 7.32 (d, $J$ = 12.0 Hz, 2H), 7.04 (d, $J$ = 12.0 Hz, 2H), 6.96 (d, $J$ = 6.0 Hz, 2H), 6.92 (d, $J$ = 6.0 Hz, 1H), 6.87 (s, 1H), 6.23 (d, $J$ = 6.0 Hz, 1H), 4.45 (d, $J$ = 6.0 Hz, 1H), 4.35-4.26 (m, 2H), 3.87 (s, 3H), 3.44 (d, $J$ = 6.0 Hz, 1H), 2.33 (td, $J$ = 12.0 Hz, 6.0 Hz, 1H), 2.19 (d, $J$ = 12.0 Hz, 1H). $^{13}$C NMR (150 MHz, $CDCl_3$) δ 194.2, 181.0, 164.1, 133.8, 131.3, 131.2, 131.0, 130.9, 130.6, 126.4, 121.1, 115.0, 114.1, 110.9, 55.6, 45.2, 43.1, 38.0, 32.7, 28.2. HRMS (ESI) calcd. for $C_{24}H_{21}BrNO_3$ $[M+H]^+$

450.0705, found 450.0707.

**4-((1S,2S,3R)-2-(4-Methoxybenzoyl)-8'-oxo-5',6'-dihydro-8'*H*-spiro[cyclopropane-1,7'-indolizin]-3-yl)benzonitrile (3ka)**

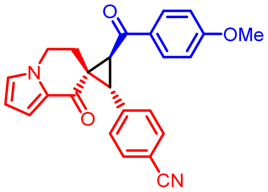


Yellow solid, 69.0 mg, 87% yield, m.p. 221-223 ºC. $^{1}$H NMR (600 MHz, $CDCl_3$) δ 8.06 (d, $J$ = 12.0 Hz, 2H), 7.50 (d, $J$ = 6.0 Hz, 2H), 7.29 (d, $J$ = 6.0 Hz, 2H), 6.96 (d, $J$ = 6.0 Hz, 2H), 6.92 (d, $J$ = 6.0 Hz, 1H), 6.89 (s, 1H), 6.24 (d, $J$ = 6.0 Hz, 1H), 4.49 (d, $J$ = 6.0 Hz, 1H), 4.34-4.29 (m, 2H), 3.88 (s, 3H), 3.52 (d, $J$ = 6.0 Hz, 1H), 2.36 (td, $J$ = 12.0 Hz, 6.0 Hz, 1H), 2.22 (d, $J$ = 12.0 Hz, 1H). $^{13}$C NMR (150 MHz, $CDCl_3$) δ 193.7, 180.6, 164.3, 140.4, 132.0, 131.1, 131.0, 130.4, 130.1, 126.7, 118.9, 115.4, 114.2, 111.1, 110.9, 55.7, 45.1, 43.3, 38.1, 32.9, 28.2. HRMS (ESI) calcd. for $C_{25}H_{20}N_2NaO_3$ $[M+Na]^+$ 419.1372, found 419.1368.

**(1S,2S,3R)-2-(4-Methoxybenzoyl)-3-(4-methoxyphenyl)-5',6'-dihydro-8'*H*-spiro[cyclopropane-1,7'-indolizin]-8'-one (3la)**

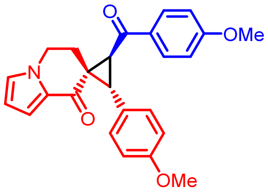


Yellow solid, 62.6 mg, 78% yield, m.p. 140-142 ºC. $^{1}$H NMR (600 MHz, $CDCl_3$) δ 8.09 (d, $J$ = 6.0 Hz, 2H), 7.08 (d, $J$ = 12.0 Hz, 2H), 6.96 (d, $J$ = 12.0 Hz, 2H), 6.92 (d, $J$ = 6.0 Hz, 1H), 6.87 (s, 1H), 6.75 (d, $J$ = 6.0 Hz, 2H), 6.23 (d, $J$ = 6.0 Hz, 1H), 4.46 (d, $J$ = 12.0 Hz, 1H), 4.36 (td, $J$ = 12.0 Hz, 6Hz 1H), 4.30-4.27 (m, 1H), 3.87 (s, 3H), 3.73 (s, 3H), 3.47 (d, $J$ = 12.0 Hz, 1H), 2.33 (td, $J$ = 12.0 Hz, 6.0 Hz, 1H), 2.20 (d, $J$ = 12.0 Hz, 1H). $^{13}$C NMR (150 MHz, $CDCl_3$) δ 194.7, 181.5, 164.0, 158.6, 131.4, 131.0, 130.8, 130.2, 126.6, 126.1, 114.8, 114.0, 113.7, 110.7, 77.2, 55.6, 55.2, 43.3, 38.4, 32.9, 28.3. HRMS (ESI) calcd. for $C_{25}H_{24}NO_4$ $[M+H]^+$ 402.1705, found 402.1706.

**(1S,2S,3R)-2-(4-Methoxybenzoyl)-3-(p-tolyl)-5',6'-dihydro-8'*H*-spiro[cyclopropane-1,7'-indolizin]-8'-one (3ma)**

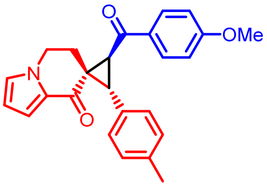

Yellow solid, 56.3 mg, 73% yield, m.p. 174-175 ºC. $^{1}$H NMR (600 MHz, $CDCl_3$) δ 8.10 (d, *J* = 6.0 Hz, 2H), 7.11-7.09 (m, 1H), 6.99-6.92 (m, 6H), 6.87 (s, 1H), 6.23 (d, *J* = 6.0 Hz, 1H), 4.49 (d, *J* = 6.0 Hz, 1H), 4.39-4.35 (m, 1H), 4.29 (d, *J* = 12.0 Hz, 1H), 3.87 (s, 3H), 3.50 (d, *J* = 6.0 Hz, 1H), 2.34 (td, *J* = 12.0 Hz, 6.0 Hz, 1H), 2.26 (s, 3H), 2.22 (d, *J* = 12.0 Hz, 1H). $^{13}$C NMR (150 MHz, $CDCl_3$) δ 194.6, 181.3, 164.0, 137.7, 134.5, 131.4, 131.0, 130.8, 130.0, 128.1, 127.9, 126.2, 126.1, 114.8, 114.0, 110.6, 55.6, 45.3, 43.2, 38.9, 32.8, 28.4, 21.5. HRMS (ESI) calcd. for $C_{25}H_{24}NO_3$ $[M+H]^+$ 386.1756, found 386.1759.

**(1S,2R,3S)-2-([1,1'-Biphenyl]-4-yl)-3-(4-methoxybenzoyl)-5',6'-dihydro-8'*H*-spiro[cyclopropane-1,7'-indolizin]-8'-one (3na)**

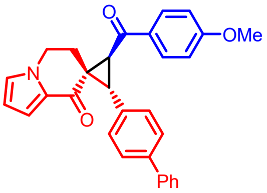

Yellow oil, 53.7 mg, 60% yield. $^{1}$H NMR (600 MHz, $CDCl_3$) δ 8.02 (d, *J* = 6.0 Hz, 2H), 7.44 (d, *J* = 6.0 Hz, 2H), 7.36 (d, *J* = 12.0 Hz, 2H), 7.31-7.29 (m, 2H), 7.22-7.19 (m, 1H), 7.16 (d, *J* = 6.0 Hz, 2H), 6.88 (d, *J* = 6.0 Hz, 2H), 6.86 (d, *J* = 6.0 Hz, 1H), 6.79 (s, 1H), 6.15 (d, *J* = 6.0 Hz, 1H), 4.46 (d, *J* = 6.0 Hz, 1H), 4.29 (td, *J* = 12.0 Hz, 6Hz 1H), 4.22-4.20 (m, 1H), 3.78 (s, 3H), 3.46 (d, *J* = 6.0 Hz, 1H), 2.27 (td, *J* = 12.0 Hz, 6.0 Hz, 1H), 2.16 (d, *J* = 12.0 Hz, 1H). $^{13}$C NMR (150 MHz, $CDCl_3$) δ 194.5, 181.3, 164.1, 140.8, 139.9, 133.8, 131.4, 131.0, 130.8, 129.6, 128.8, 127.2, 127.1, 127.0, 126.2, 114.9, 114.1, 110.8, 55.7, 45.3, 43.5, 38.7, 32.9, 28.4. HRMS (ESI) calcd. for $C_{30}H_{26}NO_3$ $[M+H]^+$ 448.1913, found 448.1917.

**(1S,2R,3S)-2-(3,4-Dichlorophenyl)-3-(4-methoxybenzoyl)-5',6'-dihydro-8'*H*-spiro[cyclopropane-1,7'-indolizin]-8'-one (3oa)**

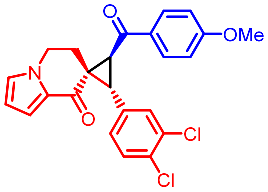


Yellow solid, 81.0 mg, 92% yield, m.p. 174-176 ºC. $^{1}$H NMR (600 MHz, $CDCl_3$) δ 7.96 (d, *J* = 12.0 Hz, 2H), 7.19 (s, 1H), 7.16 (d, *J* = 12.0 Hz, 1H), 6.90 (d, *J* = 6.0 Hz, 1H), 6.86-6.84 (m, 3H), 6.79 (s, 1H), 6.14 (d, *J* = 6.0 Hz, 1H), 4.34 (d, *J* = 6.0 Hz, 1H), 4.24-4.17 (m, 2H), 3.77 (s, 3H), 3.34 (d, *J* = 6.0 Hz, 1H), 2.23 (td, *J* = 12.0 Hz, 6.0 Hz, 1H), 2.08 (d, *J* = 12.0 Hz, 1H). $^{13}$C NMR (150 MHz, $CDCl_3$) δ 193.7, 180.6, 164.1, 135.1, 132.1, 131.2, 131.1, 131.0, 130.4, 130.1, 128.6, 126.6, 115.2, 114.1, 111.0, 55.6, 45.1, 43.0, 37.3, 32.8, 28.1. HRMS (ESI) calcd. for $C_{24}H_{19}Cl_2NNaO_3$ $[M+Na]^+$ 462.0640, found 462.0646.

**(1S,2R,3S)-2-(3,4-Dimethoxyphenyl)-3-(4-methoxybenzoyl)-5',6'-dihydro-8'*H*-spiro[cycloprop ane-1,7'-indolizin]-8'-one (3pa)**

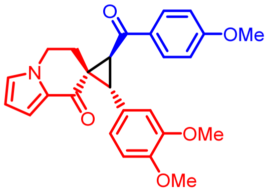


Yellow solid, 76.8 mg, 89% yield, m.p. 170-172 ºC. $^{1}$H NMR (600 MHz, $CDCl_3$) δ 8.08 (d, *J* = 12.0 Hz, 2H), 6.95 (d, *J* = 12.0 Hz, 2H), 6.93 (d, *J* = 6.0 Hz, 1H), 6.87 (s, 1H), 6.69 (s, 2H), 6.62 (s, 1H), 6.23 (d, *J* = 6.0 Hz, 1H), 4.43 (d, *J* = 6.0 Hz, 1H), 4.38-4.33 (m, 1H), 4.31-4.27 (m, 1H), 3.86 (s, 3H), 3.78 (s, 3H), 3.73 (s, 3H), 3.46 (d, *J* = 6.0 Hz, 1H), 2.33 (td, *J* = 12.0 Hz, 6.0 Hz, 1H), 2.16 (d, *J* = 12.0 Hz, 1H). $^{13}$C NMR (150 MHz, $CDCl_3$) δ 194.6, 181.4, 164.0, 148.5, 148.0, 131.5, 131.0, 130.7, 127.3, 126.1, 121.4, 114.7, 114.0, 112.4, 110.9, 110.7, 55.8, 55.8, 55.6, 45.4, 43.2, 38.4, 33.1, 28.2. HRMS (ESI) calcd. for $C_{26}H_{25}NNaO_5$ $[M+Na]^+$ 454.1630, found 454.1634.

**(1S,2S,3R)-2-(4-Methoxybenzoyl)-3-(naphthalen-2-yl)-5',6'-dihydro-8'*H*-spiro[cyclopropane-1, 7'-indolizin]-8'-one (3qa)**

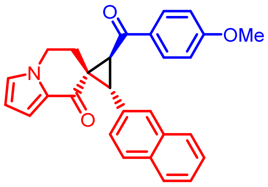


Yellow solid, 68.3 mg, 81% yield, m.p. 195-197 ºC. $^{1}$H NMR (600 MHz, $CDCl_3$) δ 8.16 (d, *J* = 6.0

Hz, 2H), 7.75 (d, $J$ = 6.0 Hz, 2H), 7.69 (d, $J$ = 12.0 Hz, 1H), 7.68 (s, 1H), 7.43-7.38 (m, 2H), 7.28 (d, $J$ = 12.0 Hz, 1H), 6.99 (d, $J$ = 12.0 Hz, 2H), 6.88 (d, $J$ = 6.0 Hz, 1H), 6.87 (s, 1H), 6.22 (d, $J$ = 6.0 Hz, 1H), 4.67 (d, $J$ = 6.0 Hz, 1H), 4.41 (td, $J$ = 12.0 Hz, 6.0 Hz, 1H), 4.29 (d, $J$ = 6.0 Hz, 1H), 3.87 (s, 3H), 3.69 (d, $J$ = 6.0 Hz, 1H), 2.37 (td, $J$ = 18.0 Hz, 6.0 Hz, 1H), 2.27 (d, $J$ = 12.0 Hz, 1H). $^{13}$C NMR (150 MHz, $CDCl_3$) δ 194.5, 181.1, 164.0, 133.2, 132.5, 132.1, 131.3, 131.0, 130.8, 128.0, 127.9, 127.8, 127.6, 127.3, 126.2, 126.1, 125.8, 114.8, 114.0, 110.7, 55.6, 45.3, 43.4, 39.0, 32.9, 28.4. HRMS (ESI) calcd. for $C_{28}H_{23}NNaO_3$ $[M+Na]^+$ 444.1576, found 444.1579.

**(1S,2S,3S)-2-(Furan-2-yl)-3-(4-methoxybenzoyl)-5',6'-dihydro-8'*H*-spiro[cyclopropane-1,7'-indolizin]-8'-one (3ra)**

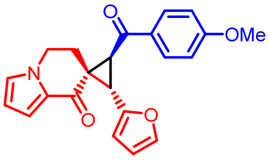


Yellow solid, 44.8 mg, 62% yield, m.p. 146-148 ºC. $^1$H NMR (600 MHz, $CDCl_3$) δ 8.06 (d, $J$ = 6.0 Hz, 2H), 7.22 (s, 1H), 6.99 (d, $J$ = 6.0 Hz, 1H), 6.95 (d, $J$ = 12.0 Hz, 2H), 6.87 (s, 1H), 6.24 (s, 2H), 6.12 (s, 1H), 4.41 (d, $J$ = 6.0 Hz, 1H), 4.37-4.32 (m, 1H), 4.25 (d, $J$ = 12.0 Hz, 1H), 3.87 (s, 3H), 3.36 (d, $J$ = 6.0 Hz, 1H), 2.31 (td, $J$ = 12.0 Hz, 6.0 Hz, 1H), 2.13 (d, $J$ = 18.0 Hz, 1H). $^{13}$C NMR (150 MHz, $CDCl_3$) δ 193.6, 180.5, 164.2, 149.5, 142.0, 131.1, 131.0, 130.5, 126.3, 115.0, 114.1, 110.8, 110.5, 107.9, 55.7, 45.1, 42.2, 32.8, 30.8, 27.9. HRMS (ESI) calcd. for $C_{22}H_{20}NO_4$ $[M+H]^+$ 362.1392, found 362.1396.

**(1R,2S,3S)-2-(4-Methoxybenzoyl)-3-(thiophen-2-yl)-5',6'-dihydro-8'*H*-spiro[cyclopropane-1,7'-indolizin]-8'-one (3sa)**

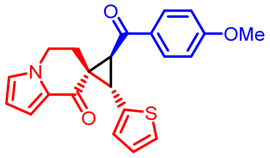


Yellow solid, 54.4 mg, 72% yield, m.p. 142-144 ºC. $^1$H NMR (600 MHz, $CDCl_3$) δ 8.07 (d, $J$ = 6.0 Hz, 2H), 7.07 (d, $J$ = 6.0 Hz, 1H), 6.98 (d, $J$ = 6.0 Hz, 1H), 6.96 (d, $J$ = 12.0 Hz, 2H), 6.87 (s, 1H), 6.86-6.84 (m, 2H), 6.24 (d, $J$ = 8.0 Hz, 1H), 4.50 (d, $J$ = 6.0 Hz, 1H), 4.34 (td, $J$ = 12.0 Hz, 6.0 Hz, 1H), 4.27 (d, $J$ = 12.0 Hz, 1H), 3.87 (s, 3H), 3.55 (d, $J$ = 6.0 Hz, 1H), 2.32 (td, $J$ = 12.0 Hz, 6.0 Hz, 1H), 2.18 (d, $J$ = 18.0 Hz, 1H). $^{13}$C NMR (150 MHz, $CDCl_3$) δ 193.8, 180.6, 164.1, 138.0, 131.2, 131.0, 130.5, 126.9, 126.6, 126.3, 124.7, 115.0, 114.1, 110.8, 55.6, 45.2, 43.7, 34.5, 32.9, 28.1. HRMS

(ESI) calcd. for $C_{22}H_{20}NO_3S$ $[M+H]^+$ 378.1164, found 378.1166.

**(1S,2S,3R)-2-(4-Methoxybenzoyl)-3-phenyl-1'*H*,3'*H*-spiro[cyclopropane-1,2'-pyrrolizin]-1'-one (3ta)**

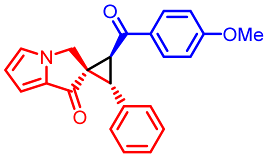


Yellow oil, 50.3 mg, 70% yield. $^1H$ NMR (600 MHz, $CDCl_3$) δ 8.02 (d, $J$ = 6.0 Hz, 2H), 7.24-7.20 (m, 4H), 7.17 (d, $J$ = 12.0 Hz, 1H), 7.02 (s, 1H), 6.90 (d, $J$ = 6.0 Hz, 2H), 6.59 (d, $J$ = 6.0 Hz, 1H), 6.43 (d, $J$ = 6.0 Hz, 1H), 4.57 (d, $J$ = 12.0 Hz, 1H), 4.34 (d, $J$ = 12.0 Hz, 1H), 4.04 (d, $J$ = 6.0 Hz, 1H), 3.81 (s, 3H), 3.49 (d, $J$ = 6.0 Hz, 1H). $^{13}C$ NMR (150 MHz, $CDCl_3$) δ 194.7, 184.5, 164.3, 133.9, 133.6, 131.0, 130.4, 129.4, 128.3, 127.5, 123.6, 116.4, 114.2, 107.7, 55.7, 49.2, 47.3, 40.4, 34.8. HRMS (ESI) calcd. for $C_{23}H_{20}NO_3$ $[M+H]^+$ 358.1443, found 358.1448.

**(1S,2S,3R)-2-(4-Methoxybenzoyl)-3-phenyl-6',7'-dihydro-5'H,9'*H*-spiro[cyclopropane-1,8'-pyrrolo[1,2-a]azepin]-9'-one (3ua)**

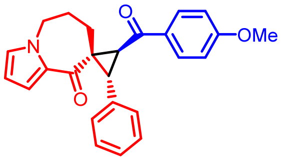


Yellow oil, 20.0 mg, 26% yield. $^1H$ NMR (600 MHz, $CDCl_3$) δ 8.08 (d, $J$ = 6.0 Hz, 2H), 7.18-7.14 (m, 4H), 7.08-7.06 (m, 1H), 6.91-6.89 (m, 3H), 6.62 (s, 1H), 6.01 (s, 1H), 4.55 (d, $J$ = 6.0 Hz, 1H), 4.26-4.21 (m, 1H), 4.04 (d, $J$ = 12.0 Hz, 1H), 3.80 (s, 3H), 3.29 (d, $J$ = 6.0 Hz, 1H), 2.11-2.07 (m, 2H), 1.86-1.80 (m, 1H), 1.76-1.72 (m, 1H). $^{13}C$ NMR (151 MHz, $CDCl_3$) δ 195.2, 185.7, 164.0, 135.2, 134.2, 131.1, 131.0, 128.5, 128.4, 128.0, 127.0, 118.4, 114.0, 109.1, 55.7, 48.6, 47.2, 36.2, 34.9, 26.4, 23.4. HRMS (ESI) calcd. for $C_{25}H_{24}NO_3$ $[M+H]^+$ 386.1756, found 386.1759.

**(1S,2S,3R)-2-Benzoyl-3-(3,4-dichlorophenyl)-5',6'-dihydro-8'*H*-spiro[cyclopropane-1,7'-indolizin]-8'-one (3ob)**

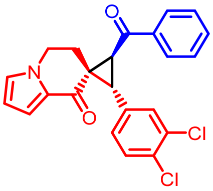


Yellow solid, 73.9 mg, 90% yield, m.p. 162-164 °C. $^1H$ NMR (600 MHz, $CDCl_3$) δ 7.99 (d, $J$ = 6.0

Hz, 2H), 7.53-7.51 (m, 1H), 7.42-7.39 (m, 2H), 7.19-7.17 (m, 2H), 6.90 (d, $J$ = 12.0 Hz, 1H), 6.87 (d, $J$ = 6.0 Hz, 1H), 6.80 (s, 1H), 6.16 (s, 1H), 4.40 (d, $J$ = 6.0 Hz, 1H), 4.26-4.19 (m, 2H), 3.37 (d, $J$ = 6.0 Hz, 1H), 2.26 (td, $J$ = 12.0 Hz, 6.0 Hz, 1H), 2.11 (d, $J$ = 12.0 Hz, 1H). $^{13}$C NMR (150 MHz, $CDCl_3$) δ 195.6, 180.4, 137.3, 134.9, 133.9, 132.2, 131.3, 131.1, 130.2, 129.0, 128.6, 128.5, 126.6, 115.4, 111.1, 45.1, 43.5, 37.5, 33.1, 28.1. HRMS (ESI) calcd. for $C_{23}H_{17}Cl_2NNaO_2$ $[M+Na]^+$ 432.0534, found 432.0530.

**(1S,2S,3R)-2-(2-Chlorobenzoyl)-3-(3,4-dichlorophenyl)-5',6'-dihydro-8'*H*-spiro[cyclopropane-1,7'-indolizin]-8'-one (3oc)**

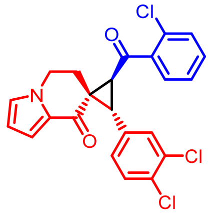


Yellow solid, 65.8 mg, 74% yield, m.p. 167-169 ºC. $^1$H NMR (600 MHz, $CDCl_3$) δ 7.51 (d, $J$ = 12.0 Hz, 1H), 7.39-7.35 (m, 2H), 7.30-7.27 (m, 1H), 7.19-7.18 (m, 2H), 6.91 (d, $J$ = 12.0 Hz, 1H), 6.85 (d, $J$ = 6.0 Hz, 1H), 6.83-6.82 (m, 1H), 6.17 (d, $J$ = 6.0 Hz, 1H), 4.30-4.24 (m, 2H), 4.25 (d, $J$ = 6.0 Hz, 1H), 3.38 (d, $J$ = 6.0 Hz, 1H), 2.45-2.36 (m, 2H). $^{13}$C NMR (150 MHz, $CDCl_3$) δ 198.3, 180.0, 139.2, 134.7, 132.8, 132.3, 132.1, 131.4, 131.3, 131.2, 131.0, 130.2, 129.9, 128.5, 127.3, 126.5, 115.5, 111.1, 45.3, 45.1, 39.5, 36.9, 28.0. HRMS (ESI) calcd. for $C_{23}H_{17}Cl_3NO_2$ $[M+H]^+$ 444.0325, found 444.0328.

**(1S,2R,3S)-2-(3,4-Dichlorophenyl)-3-(2-methoxybenzoyl)-5',6'-dihydro-8'*H*-spiro[cyclopropane-1,7'-indolizin]-8'-one (3od)**

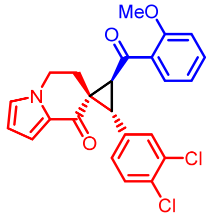


Yellow solid, 74.0 mg, 84% yield, m.p. 150-152 ºC. $^1$H NMR (600 MHz, $CDCl_3$) δ 7.61 (d, $J$ = 6.0 Hz, 1H), 7.43-7.40 (m, 1H), 7.22 (d, $J$ = 6.0 Hz, 1H), 7.17 (d, $J$ = 6.0 Hz, 1H),6.95-6.93 (m, 1H), 6.92 (dd, $J$ = 6.0 Hz, 6.0 Hz, 1H), 6.89 (d, $J$ = 6.0 Hz, 1H), 6.84 (d, $J$ = 6.0 Hz, 1H), 6.79-6.78 (m, 1H), 6.15 (d, $J$ = 6.0 Hz, 1H), 4.31 (d, $J$ = 6.0 Hz, 1H), 4.22-4.14 (m, 2H), 3.69 (s, 3H), 3.36 (d, $J$ = 6.0 Hz, 1H), 2.33-2.25 (m, 2H). $^{13}$C NMR (150 MHz, $CDCl_3$) δ 197.5, 181.3, 159.3, 135.6, 134.4, 132.0,

131.3, 130.9, 130.3, 130.0, 128.7, 128.6, 126.1, 120.8, 114.7, 111.8, 110.8, 55.7, 45.1, 43.1, 38.9, 38.4, 28.2. HRMS (ESI) calcd. for $C_{24}H_{20}Cl_2NO_3$ $[M+H]^+$ 440.0820, found 440.0822.

**(1S,2R,3S)-2-(3,4-Dichlorophenyl)-3-(2-methylbenzoyl)-5',6'-dihydro-8'*H*-spiro[cyclopropane-1,7'-indolizin]-8'-one (3oe)**

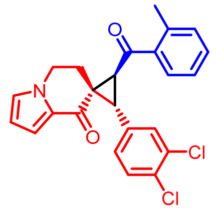


Yellow solid, 52.6 mg, 62% yield, m.p. 134-136 °C. $^1$H NMR (600 MHz, $CDCl_3$) δ 7.74 (d, $J$ = 6.0 Hz, 1H), 7.35-7.32 (m, 1H), 7.22-7.19 (m, 3H), 7.18 (d, $J$ = 6.0 Hz, 1H), 6.90 (d, $J$ = 6.0 Hz, 1H), 6.87 (d, $J$ = 6.0 Hz, 1H), 6.82-6.81 (m, 1H), 6.18 (d, $J$ = 6.0 Hz, 1H), 4.26-4.23 (m, 3H), 3.35 (d, $J$ = 6.0 Hz, 1H), 2.49 (s, 3H), 2.36-2.31 (m, 1H), 2.22 (d, $J$ = 12.0 Hz, 1H). $^{13}$C NMR (150 MHz, $CDCl_3$) δ 198.8, 180.3, 138.7, 137.8, 134.9, 132.3, 132.2, 132.2, 131.3, 131.2, 130.2, 129.9, 128.5, 126.6, 126.3, 115.4, 111.1, 45.2, 44.1, 38.0, 35.6, 28.1, 21.5. HRMS (ESI) calcd. for $C_{24}H_{19}Cl_2NNaO_2$ $[M+Na]^+$ 446.0691, found 446.0695.

**(1S,2S,3R)-2-(3-Chlorobenzoyl)-3-(3,4-dichlorophenyl)-5',6'-dihydro-8'*H*-spiro[cyclopropane-1,7'-indolizin]-8'-one (3of)**

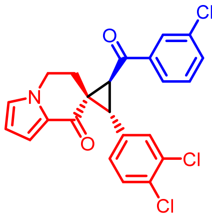


Yellow solid, 80.1 mg, 90% yield, m.p. 155-157 °C. $^1$H NMR (600 MHz, $CDCl_3$) δ 7.94-7.93 (m, 1H), 7.87 (d, $J$ = 6.0 Hz, 1H), 7.49 (d, $J$ = 12.0 Hz, 1H), 7.37-7.34 (m, 1H), 7.19 (s, 1H), 7.18 (d, $J$ = 4.8 Hz, 1H), 6.90 (d, $J$ = 6.0 Hz, 1H), 6.87 (d, $J$ = 6.0 Hz, 1H), 6.82 (s, 1H), 6.17 (d, $J$ = 6.0 Hz, 1H), 4.36 (d, $J$ = 12.0 Hz, 1H), 4.25-4.22 (m, 2H), 3.37 (d, $J$ = 6.0 Hz, 1H), 2.29-2.24 (m, 1H), 2.11 (d, $J$ = 12.0 Hz, 1H). $^{13}$C NMR (150 MHz, $CDCl_3$) δ 194.5, 180.1, 138.8, 135.4, 134.6, 133.8, 132.3, 131.4, 131.2, 131.0, 130.3, 130.2, 128.5, 128.4, 127.0, 126.7, 115.5, 111.2, 45.1, 43.9, 37.7, 33.0, 28.1. HRMS (ESI) calcd. for $C_{23}H_{17}Cl_3NO_2$ $[M+H]^+$ 444.0325, found 444.0328.

**(1S,2R,3S)-2-(3,4-Dichlorophenyl)-3-(3-nitrobenzoyl)-5',6'-dihydro-8'*H*-spiro[cyclopropane-1,**

**7'-indolizin]-8'-one (3og)**

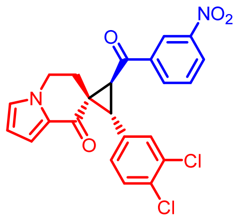

Yellow solid, 12.7 mg, 14% yield, m.p. 202-204 ºC. $^1$H NMR (600 MHz, $CDCl_3$) δ 8.88 (d, *J* = 6.0 Hz, 1H), 8.46 (d, *J* = 6.0 Hz, 1H), 8.40 (d, *J* = 6.0 Hz, 1H), 7.74-7.72 (m, 1H), 7.29 (d, *J* = 12.0 Hz, 1H), 7.28 (d, *J* = 6.0 Hz, 1H), 6.99-6.97 (m, 2H), 6.93-6.92 (m, 1H), 6.28 (d, *J* = 6.0 Hz, 1H), 4.50 (d, *J* = 6.0 Hz, 1H), 4.37-7.34 (m, 2H), 3.49 (d, *J* = 12.0 Hz, 1H), 2.39-2.34 (m, 1H), 2.24 (d, *J* = 12.0 Hz, 1H). $^{13}$C NMR (150 MHz, $CDCl_3$) δ 193.9, 179.8, 148.9, 138.6, 134.4, 134.3, 132.5, 131.7, 131.2, 131.0, 130.4, 130.4, 128.4, 128.0, 126.9, 123.2, 115.9, 111.4, 45.2, 44.4, 38.1, 33.1, 28.2. HRMS (ESI) calcd. for $C_{23}H_{17}Cl_2N_2O_4$ $[M+H]^+$ 455.0565, found 455.0569.

**(1S,2R,3S)-2-(3,4-Dichlorophenyl)-3-(3-methylbenzoyl)-5',6'-dihydro-8'*H*-spiro[cyclopropane-1,7'-indolizin]-8'-one (3oh)**

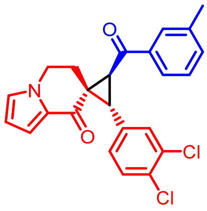

Yellow solid, 74.7 mg, 88% yield, m.p. 143-145 ºC. $^1$H NMR (600 MHz, $CDCl_3$) δ 7.78-7.77 (m, 2H), 7.32 (d, *J* = 6.0 Hz, 1H), 7.29-7.26 (m, 1H), 7.18 (s, 1H), 7.16 (d, *J* = 6.0 Hz, 1H), 6.89 (d, *J* = 12.0 Hz, 1H), 6.85 (d, *J* = 6.0 Hz, 1H), 6.78 (s, 1H), 6.14 (d, *J* = 12.0 Hz, 1H), 4.38 (d, *J* = 6.0 Hz, 1H), 4.24-4.16 (m, 2H), 3.35 (d, *J* = 6.0 Hz, 1H), 2.32 (s, 3H), 2.23 (dd, *J* = 12.0 Hz, 6.0 Hz, 1H), 2.11 (d, *J* = 12.0 Hz, 1H). $^{13}$C NMR (151 MHz, $CDCl_3$) δ 195.8, 180.4, 138.8, 137.4, 135.0, 134.7, 132.2, 131.3, 131.2, 131.1, 130.1, 128.9, 128.8, 128.5, 126.6, 126.0, 115.3, 111.0, 45.1, 43.5, 37.5, 33.0, 28.1, 21.5. found 446.0821. HRMS (ESI) calcd. for $C_{24}H_{20}Cl_2NO_2$ $[M+H]^+$ 424.0871, found 424.0870.

**(1S,2S,3R)-2-(4-Chlorobenzoyl)-3-(3,4-dichlorophenyl)-5',6'-dihydro-8'*H*-spiro[cyclopropane-1,7'-indolizin]-8'-one (3oi)**

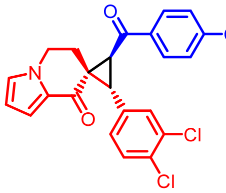


Yellow solid, 71.2 mg, 80% yield, m.p. 142-144 °C. $^{1}$H NMR (600 MHz, $CDCl_3$) δ 8.02 (d, *J* = 12.0 Hz, 2H), 7.48 (d, *J* = 12.0 Hz, 1H), 7.28 (d, *J* = 12.0 Hz, 1H), 7.26 (d, *J* = 6.0 Hz, 1H), 6.98-6.96 (m, 1H), 6.91 (s, 1H), 6.26 (d, *J* = 6.0 Hz, 1H), 4.44 (d, *J* = 6.0 Hz, 1H), 4.34-4.30 (m, 2H), 3.44 (d, *J* = 6.0 Hz, 1H), 2.37-2.32 (m, 1H), 2.18 (d, *J* = 18.0 Hz, 1H). $^{13}$C NMR (150 MHz, $CDCl_3$) δ 194.6, 180.3, 140.6, 135.7, 134.7, 132.4, 131.5, 131.3, 131.1, 130.3, 130.1, 129.4, 128.5, 126.8, 115.6, 111.3, 45.2, 43.7, 37.6, 33.0, 28.2. HRMS (ESI) calcd. for $C_{23}H_{16}Cl_3NNaO_2$ $[M+Na]^+$ 466.0144, found 466.0148.

**4-((1S,2R,3S)-2-(3,4-Dichlorophenyl)-8'-oxo-5',6'-dihydro-8'*H*-spiro[cyclopropane-1,7'-indolizine]-3-carbonyl)benzonitrile (3oj)**

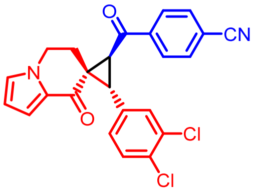


Yellow solid, 35.7 mg, 41% yield, m.p. 249-251 °C. $^{1}$H NMR (600 MHz, $CDCl_3$) δ 8.09 (d, *J* = 6.0 Hz, 2H), 7.73 (d, *J* = 12.0 Hz, 2H), 7.21 (d, *J* = 6.0 Hz, 1H), 7.19 (s, 1H), 6.90 (d, *J* = 6.0 Hz, 2H), 6.85 (s, 1H), 6.21 (d, *J* = 6.0 Hz, 1H), 4.40 (d, *J* = 6.0 Hz, 1H), 4.28-4.26 (m, 2H), 3.40 (d, *J* = 12.0 Hz, 1H), 2.30-2.25 (m, 1H), 2.12 (d, *J* = 12.0 Hz, 1H). $^{13}$C NMR (150 MHz, $CDCl_3$) δ 194.7, 179.9, 140.2, 134.4, 132.9, 132.4, 131.6, 131.2, 131., 130.3, 129.1, 128.4, 127.0 117.9, 117.1, 115.8, 111.4, 45.2, 44.3, 37.9, 33.2, 28.2. HRMS (ESI) calcd. for $C_{24}H_{17}Cl_2N_2O_2$ $[M+H]^+$ 435.0667, found 435.0669.

**(1S,2R,3S)-2-(3,4-Dichlorophenyl)-3-(4-methylbenzoyl)-5',6'-dihydro-8'*H*-spiro[cyclopropane-1,7'-indolizin]-8'-one (3ok)**

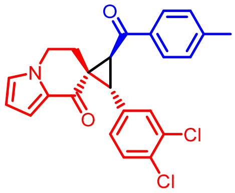


Yellow solid, 79.9 mg, 94% yield, m.p. 138-140 °C. $^{1}$H NMR (600 MHz, $CDCl_3$) δ 7.98 (d, *J* = 12.0

Hz, 2H), 7.30 (d, $J$ = 6.0 Hz, 2H), 7.28 (d, $J$ = 6.0 Hz, 2H), 6.99 (d, $J$ = 12.0 Hz, 1H), 6.96 (d, $J$ = 4.2 Hz, 1H), 6.89 (s, 1H), 6.26 (d, $J$ = 6.0 Hz, 1H), 4.46 (d, $J$ = 6.0 Hz, 1H), 4.35-4.27 (m, 2H), 3.44 (d, $J$ = 12.0 Hz, 1H), 2.43 (s, 3H), 2.37-2.32 (m, 1H), 2.20 (d, $J$ = 18.0 Hz, 1H). $^{13}$C NMR (150 MHz, $CDCl_3$) δ 195.2, 180.6, 145.0, 135.0, 135.0, 132.3, 131.4, 131.3, 131.2, 130.2, 129.7, 128.8, 128.6, 126.6, 115.4, 111.1, 45.2, 43.3, 37.5, 33.1, 28.2, 21.9. HRMS (ESI) calcd. for $C_{24}H_{20}Cl_2NO_2$ $[M+H]^+$ 424.0871, found 424.0873.

**(1S,2S,3R)-2-([1,1'-Biphenyl]-4-carbonyl)-3-(3,4-dichlorophenyl)-5',6'-dihydro-8'*H*-spiro[cyclopropane-1,7'-indolizin]-8'-one (3ol)**

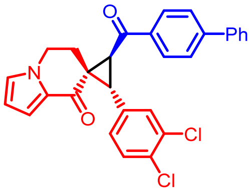


Yellow solid, 60.3 mg, 62% yield, m.p. 162-164 ºC. $^1$H NMR (600 MHz, $CDCl_3$) δ 8.17 (d, $J$ = 6.0 Hz, 2H), 7.73 (d, $J$ = 6.0 Hz, 2H), 7.64 (d, $J$ = 12.0 Hz, 2H), 7.50-7.47 (m, 2H), 7.43-7.41 (m, 1H), 7.32 (s, 1H), 7.29 (d, $J$ = 6.0 Hz, 1H), 7.02 (d, $J$ = 12.0 Hz, 1H), 6.98 (d, $J$ = 6.0 Hz, 1H), 6.91 (s, 1H), 6.27 (d, $J$ = 6.0 Hz, 1H), 4.54 (d, $J$ = 6.0 Hz, 1H), 4.37-4.29 (m, 2H), 3.49 (d, $J$ = 12.0 Hz, 1H), 2.39 (td, $J$ = 12.0 Hz, 6.0 Hz, 1H), 2.24 (d, $J$ = 12.0 Hz, 1H). $^{13}$C NMR (150 MHz, $CDCl_3$) δ 195.1, 180.5, 146.6, 139.7, 136.0, 135.0, 132.3, 131.3, 131.1, 130.2, 129.3, 129.1, 128.5, 128.5, 127.6, 127.4, 126.6, 115.4, 111.1, 45.2, 43.5, 37.5, 33.2, 28.2. HRMS (ESI) calcd. for $C_{29}H_{22}Cl_2NO_2$ $[M+H]^+$ 486.1028, found 486.1024.

**(1S,2R,3S)-2-(3,4-Dichlorophenyl)-3-(3,4-dimethoxybenzoyl)-5',6'-dihydro-8'*H*-spiro[cyclopropane-1,7'-indolizin]-8'-one (3om)**

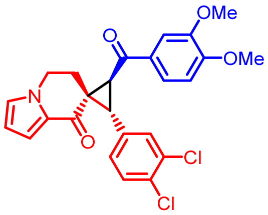


Yellow solid, 71.5 mg, 76% yield, m.p. 177-179 ºC. $^1$H NMR (600 MHz, $CDCl_3$) δ 7.70 (d, $J$ = 12.0 Hz, 1H), 7.51 (d, $J$ = 6.0 Hz, 1H), 7.21 (s, 1H), 7.20-7.19 (m, 1H), 6.92 (d, $J$ = 6.0 Hz, 1H), 6.87 (d, $J$ = 6.0 Hz, 1H), 6.84 (d, $J$ = 12.0 Hz, 1H), 6.81 (s, 1H), 6.18 (d, $J$ = 6.0 Hz, 1H), 4.37 (d, $J$ = 6.0 Hz, 1H), 4.27-4.20 (m, 2H), 3.88 (s, 3H), 3.87 (s, 3H), 3.36 (d, $J$ = 6.0 Hz, 1H), 2.28 (dd, $J$ = 12.0 Hz,

6.0 Hz, 1H), 2.11 (d, $J$ = 12.0 Hz, 1H). $^{13}$C NMR (150 MHz, $CDCl_3$) δ 193.9, 180.6, 154.0, 149.3, 135.1, 132.2, 131.3, 131.2, 131.2, 130.6, 130.2, 128.5, 126.6, 124.0, 115.3, 111.0, 110.3, 110.1, 56.3, 56.1, 45.1, 43.1, 37.4, 32.8, 28.2. HRMS (ESI) calcd. for $C_{25}H_{21}Cl_2NNaO_4$ $[M+Na]^+$ 492.0745, found 492.0748.

**(1S,2S,3R)-2-(2-Naphthoyl)-3-(3,4-dichlorophenyl)-5',6'-dihydro-8'*H*-spiro[cyclopropane-1,7'-indolizin]-8'-one (3on)**

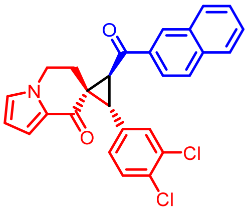


Yellow solid, 79.2 mg, 86% yield, m.p. 212-215 ºC. $^1$H NMR (600 MHz, $CDCl_3$) δ 8.65 (s, 1H), 8.11 (d, $J$ = 12.0 Hz, 1H), 8.01 (d, $J$ = 12.0 Hz, 1H), 7.91 (d, $J$ = 6.0 Hz, 1H), 7.88 (d, $J$ = 12.0 Hz, 1H), 7.63-7.60 (m, 1H), 7.57-7.55 (m, 1H), 7.33 (d, $J$ = 6.0 Hz, 1H), 7.28 (d, $J$ = 12.0 Hz, 1H), 7.03 (d, $J$ = 6.0 Hz, 1H), 7.00 (d, $J$ = 6.0 Hz, 1H), 6.89 (s, 1H), 6.26 (d, $J$ = 6.0 Hz, 1H), 4.67 (d, $J$ = 6.0 Hz, 1H), 4.36-4.27 (m, 2H), 3.52 (d, $J$ = 6.0 Hz, 1H), 2.38 (dd, $J$ = 12.0 Hz, 6.0 Hz, 1H), 2.26 (d, $J$ = 12.0 Hz, 1H). $^{13}$C NMR (150 MHz, $CDCl_3$) δ 195.4, 180.5, 135.9, 135.0, 134.6, 132.5, 132.2, 131.3, 131.2, 131.1, 130.7, 130.1, 129.9, 129.0, 128.8, 128.6, 127.9, 127.1, 126.6, 123.8, 115.4, 111.0, 45.1, 43.6, 37.6, 33.0, 28.1. HRMS (ESI) calcd. for $C_{27}H_{20}Cl_2NO_2$ $[M+H]^+$ 460.0871, found 460.0875.

**(1S,2R,3S)-2-(3,4-Dichlorophenyl)-3-(thiophene-2-carbonyl)-5',6'-dihydro-8'*H*-spiro[cyclopropane-1,7'-indolizin]-8'-one (3oo)**

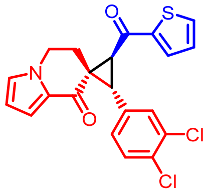


Yellow solid, 66.6 mg, 80% yield, m.p. 173-175 ºC. $^1$H NMR (600 MHz, $CDCl_3$) δ 7.88 (d, $J$ = 6.0 Hz, 1H), 7.62 (d, $J$ = 6.0 Hz, 1H), 7.20 (s, 1H), 7.18 (d, $J$ = 6.0 Hz, 1H), 7.09-7.08 (m, 1H), 6.91 (d, $J$ = 12.0 Hz, 1H), 6.84 (d, $J$ = 6.0 Hz, 1H), 6.81 (s, 1H), 6.16 (d, $J$ = 6.0 Hz, 1H), 4.29 (d, $J$ = 6.0 Hz, 1H), 4.24-4.22 (m, 2H), 3.33 (d, $J$ = 6.0 Hz, 1H), 2.34-2.28 (m, 1H), 2.25 (d, $J$ = 12.0 Hz, 1H). $^{13}$C NMR (150 MHz, $CDCl_3$) δ 188.0, 180.4, 144.7, 134.9, 134.8, 133.6, 132.2, 131.3, 131.2, 131.0, 130.2, 128.7, 128.5, 126.6, 115.4, 111.0, 45.1, 43.4, 37.9, 33.4, 28.0. HRMS (ESI) calcd. for

$C_{21}H_{15}Cl_2NNaO_2S$ $[M+Na]^+$ 438.0098, found 438.0100.

**(1S,2S,3R)-2-Acetyl-3-(3,4-dichlorophenyl)-5',6'-dihydro-8'*H*-spiro[cyclopropane-1,7'-indolizin]-8'-one (3op)**

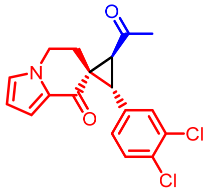


Yellow solid, 59.9 mg, 86% yield, m.p. 126-128 °C. $^1$H NMR (600 MHz, $CDCl_3$) δ 7.22 (d, *J* = 6.0 Hz, 1H), 7.21 (s, 1H), 6.91 (d, *J* = 12.0 Hz, 1H), 6.87 (br.s, 2H), 6.21 (d, *J* = 6.0 Hz, 1H), 4.31-4.23 (m, 2H), 3.79 (d, *J* = 6.0 Hz, 1H), 3.16 (d, *J* = 12.0 Hz, 1H), 2.41 (s, 3H), 2.34-2.32 (m, 2H). $^{13}$C NMR (150 MHz, $CDCl_3$) δ 204.2, 180.4, 134.6, 132.1, 131.2, 131.1, 131.0, 130.1, 128.4, 126.5, 115.3, 111.0, 45.1, 43.5, 38.4, 35.6, 32.4, 27.7. HRMS (ESI) calcd. for $C_{18}H_{16}Cl_2NO_2$ $[M+H]^+$ 348.0558, found 348.0562.

**(1S,2S,3R)-2-(3-Bromobenzoyl)-3-(4-methoxyphenyl)-5',6'-dihydro-8'*H*-spiro[cyclopropane-1,7'-indolizin]-8'-one (3lq)**

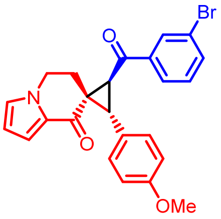


Yellow oil, 59.4 mg, 66% yield. $^1$H NMR (600 MHz, $CDCl_3$) δ 8.20 (s, 1H), 8.03 (d, *J* = 12.0 Hz, 1H), 7.72 (d, *J* = 12.0 Hz, 1H), 7.39-7.36 (m, 1H), 7.07 (d, *J* = 6.0 Hz, 2H), 6.93 (d, *J* = 6.0 Hz, 1H), 6.88 (s, 1H), 6.75 (d, *J* = 12.0 Hz, 2H), 6.24 (d, *J* = 6.0 Hz, 1H), 4.46 (d, *J* = 6.0 Hz, 1H), 4.39-4.29 (m, 2H), 3.73 (s, 3H), 3.49 (d, *J* = 6.0 Hz, 1H), 2.35 (dd, *J* = 12.0 Hz, 6.0 Hz, 1H), 2.23 (d, *J* = 12.0 Hz, 1H). $^{13}$C NMR (150 MHz, $CDCl_3$) δ 195.3, 180.9, 158.7, 139.4, 136.5, 131.3, 131.3, 130.5, 130.2, 127.5, 126.3, 126.2, 123.3, 115.0, 113.8, 110.9, 55.3, 44.2, 39.0, 33.1, 28.3. HRMS (ESI) calcd. for $C_{24}H_{20}BrNNaO_3$ $[M+Na]^+$ 472.0524, found 472.0528.

**(1S,2S,3R)-2-(3-Methoxybenzoyl)-3-(4-methoxyphenyl)-5',6'-dihydro-8'*H*-spiro[cyclopropane-1,7'-indolizin]-8'-one (3lr)**

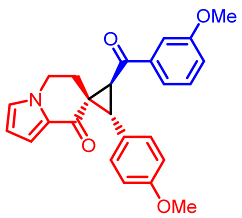


Yellow oil, 75.5 mg, 94% yield. $^{1}H$ NMR (600 MHz, $CDCl_3$) δ 7.72 (d, $J$ = 6.0 Hz, 1H), 7.59 (s, 1H), 7.41-7.39 (m, 1H), 7.14 (d, $J$ = 6.0 Hz, 1H), 7.09 (d, $J$ = 6.0 Hz, 2H), 6.92 (d, $J$ = 6.0 Hz, 1H), 6.87 (s, 1H), 6.75 (d, $J$ = 12.0 Hz, 2H), 6.23 (d, $J$ = 6.0 Hz, 1H), 4.51 (d, $J$ = 6.0 Hz, 1H), 4.36 (dd, $J$ = 12.0 Hz, 6.0 Hz, 1H), 4.29 (d, $J$ = 12.0 Hz, 1H), 3.85 (s, 3H), 3.72 (s, 3H), 3.49 (d, $J$ = 6.0 Hz, 1H), 2.35 (dd, $J$ = 12.0 Hz, 6.0 Hz, 1H), 2.21 (d, $J$ = 12.0 Hz, 1H). $^{13}C$ NMR (150 MHz, $CDCl_3$) δ 196.2, 181.1, 160.0, 158.6, 139.0, 131.3, 130.2, 129.9, 126.4, 126.1, 121.5, 120.2, 114.8, 113.7, 112.3, 110.7, 55.5, 55.2, 45.3, 43.7, 38.7, 33.3, 28.3. HRMS (ESI) calcd. for $C_{25}H_{24}NO_4$ $[M+H]^+$ 402.1705, found 402.1708.

**7-(3-(4-Methoxyphenyl)-3-oxo-1-phenylpropyl)-6,7-dihydroindolizin-8(5*H*)-one (4-major and 4-minor)**

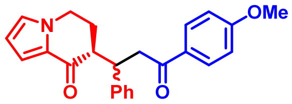


**4-major** and **4-minor**

**4-major**: white solid, 19.4 mg, 52% yield, m.p. 164-166 ºC. $^{1}H$ NMR (600 MHz, $CDCl_3$) δ 7.87 (d, $J$ = 6.0 Hz, 2H), 7.31-7.27 (m, 4H), 7.20-7.17 (m, 1H), 6.99 (d, $J$ = 6.0 Hz, 1H), 6.87 (d, $J$ = 6.0 Hz, 2H), 6.81 (s, 1H), 6.25 (d, $J$ = 6.0 Hz, 1H), 4.24-4.19 (m, 1H), 3.99-3.95 (m, 2H), 3.83 (s, 3H), 3.65 (dd, $J$ = 18.0 Hz, 6.0 Hz, 1H), 3.46 (dd, $J$ = 12.0 Hz, 6.0 Hz, 1H), 2.87-2.84 (m, 1H), 2.23-2.17 (m, 1H), 1.83-1.78 (m, 1H). $^{13}C$ NMR (150 MHz, $CDCl_3$) δ 197.1, 188.6, 163.5, 142.3, 130.5, 130.4, 130.2, 128.8, 128.5, 126.9, 125.7, 114.7, 113.7, 111.0, 55.6, 49.3, 43.1, 42.7, 39.7, 26.5. HRMS (ESI) calcd. for $C_{24}H_{24}NO_3$ $[M+H]^+$ 374.1756, found 374.1759.

**4-minor**: white solid, 9.0 mg, 24% yield, m.p. 145-147 ºC. $^{1}H$ NMR (600 MHz, $CDCl_3$) δ 8.02 (d, $J$ = 12.0 Hz, 2H), 7.32 (d, $J$ = 6.0 Hz, 2H), 7.27-7.25 (m, 2H), 7.19-7.17 (m, 1H), 7.03 (d, $J$ = 12.0 Hz, 1H), 6.92 (d, $J$ = 12.0 Hz, 2H), 6.77 (s, 1H), 6.24 (s, 1H), 4.23-4.20 (m, 1H), 4.10 (d, $J$ = 12.0 Hz, 1H), 3.96-3.92 (m, 1H), 3.85 (s, 3H), 3.74 (dd, $J$ = 18.0 Hz, 6.0 Hz, 1H), 3.42 (dd, $J$ = 18.0 Hz, 6.0 Hz, 1H), 2.89-2.86 (m, 1H), 2.24-2.17 (m, 2H). $^{13}C$ NMR (150 MHz, $CDCl_3$) δ 197.8, 188.1, 163.5, 141.8, 131.3, 130.8, 130.2, 128.9, 128.6, 126.8, 125.6, 114.4, 113.8, 110.9, 55.6, 50.4, 45.1, 41.6,

39.9, 26.4. HRMS (ESI) calcd. for $C_{24}H_{24}NO_3$ $[M+H]^+$ 374.1756, found 374.1758.

**2-(4-Methoxyphenyl)-4-phenyl-5,6-dihydropyrrolo[1,2-*h*][1,7]naphthyridine (5)**

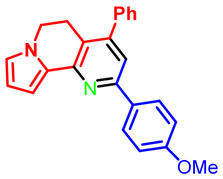


Yellow oil, 43.0 mg, 68% yield. $^1$H NMR (600 MHz, $CDCl_3$) δ 8.08 (d, *J* = 6.0 Hz, 2H), 7.50-7.48 (m, 2H), 7.45-7.42 (m, 1H), 7.40-7.38 (m, 3H), 7.07 (d, *J* = 6.0 Hz, 1H), 6.99 (d, *J* = 6.0 Hz, 2H), 6.75 (s, 1H), 6.31 (d, *J* = 6.0 Hz, 1H), 4.05 (t, *J* = 6.0 Hz, 2H), 3.87 (s, 3H), 3.09 (t, *J* = 6.0 Hz, 2H). $^{13}$C NMR (150 MHz, $CDCl_3$) δ 160.5, 155.0, 149.1, 148.5, 139.4, 132.2, 131.2, 128.8, 128.7, 128.3, 128.2, 122.0, 120.9, 117.6, 114.1, 109.6, 107.8, 55.5, 43.9, 26.3. HRMS (ESI) calcd. for $C_{24}H_{20}N_2NaO$ $[M+Na]^+$ 375.1473, found 375.1475.

**(1S,2S,3R)-3'-(1*H*-Benzo[*d*][1,2,3]triazol-1-yl)-2-(4-methoxybenzoyl)-3-phenyl-5',6'-dihydro-8'*H*-spiro[cyclopropane-1,7'-indolizin]-8'-one (6)**

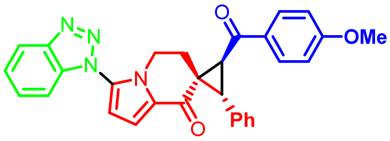


Yellow oil, 26.9 mg, 55% yield. $^1$H NMR (600 MHz, $CDCl_3$) δ 8.18 (d, *J* = 12.0 Hz, 1H), 8.10 (d, *J* = 12.0 Hz, 2H), 7.62-7.59 (m, 1H), 7.56 (d, *J* = 6.0 Hz, 1H), 7.50-7.48 (m, 1H), 7.28-7.26 (overlapped m, 2H), 7.23-7.19 (m, 3H), 7.08 (d, *J* = 6.0 Hz, 1H), 6.98 (d, *J* = 12.0 Hz, 2H), 6.50 (d, *J* = 6.0 Hz, 1H), 4.56 (d, *J* = 6.0 Hz, 1H), 4.38 (dd, *J* = 12.0 Hz, 6.0 Hz, 1H), 4.26-4.23 (m, 1H), 3.89 (s, 3H), 3.59 (d, *J* = 12.0 Hz, 1H), 2.38 (dd, *J* = 18.0 Hz, 6.0 Hz, 1H), 2.28 (d, *J* = 18.0 Hz, 1H). $^{13}$C NMR (150 MHz, $CDCl_3$) δ 194.2, 182.0, 164.2, 145.6, 134.3, 134.0, 131.1, 130.7, 130.5, 129.4, 129.3, 128.4, 127.4, 127.4, 125.2, 120.6, 114.1, 114.1, 110.1, 106.5, 55.7, 43.3, 43.2, 39.5, 32.7, 27.7. HRMS (ESI) calcd. for $C_{30}H_{25}N_4O_3$ $[M+H]^+$ 489.1927, found 489.1931.

## IV. References


[1] Gracia, S.; Cazorla, C.; Métay, E.; Pellet-Rostaing, S; Lemaire, M. Synthesis of 3-Aryl-8-oxo-5,6,7,8-tetrahydroindolizines via a Palladium-catalyzed Arylation and Heteroarylation *J. Org. Chem.* **2009**, *74*, 3160–3163.

[2] Liu, B.; Li, X.; Zhang, J.; Du, L.; Zeng, R. Synthesis of Novel Spiro[cyclopropane-indolizine] Derivatives via Magnesium-mediated Conjugate Addition of Bromoform. *J. Chem. Res.* **2013**, *37*, 681–683.

[3] Nambu, H.; Onuki, Y.; Ono, N.; Tsuge, K.; Yakura, T. Ring-opening Cyclization of Spirocyclopropanes with Stabilized Sulfonium Ylides for the Construction of a Chromane Skeleton. *Chem. Commun.* **2019**, *55*, 6539–6542.

# V. Copies of NMR spectra

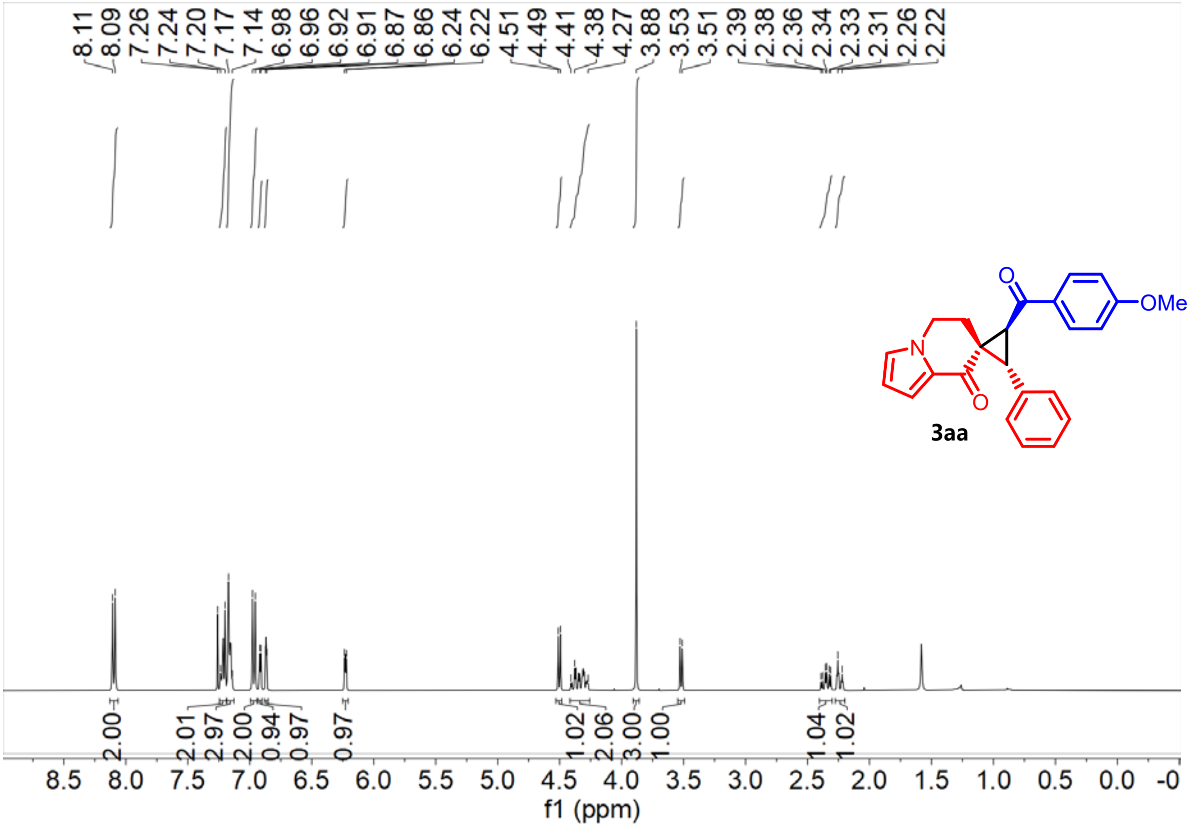


**Figure S1.** $^{1}$H-NMR of compound **3aa**

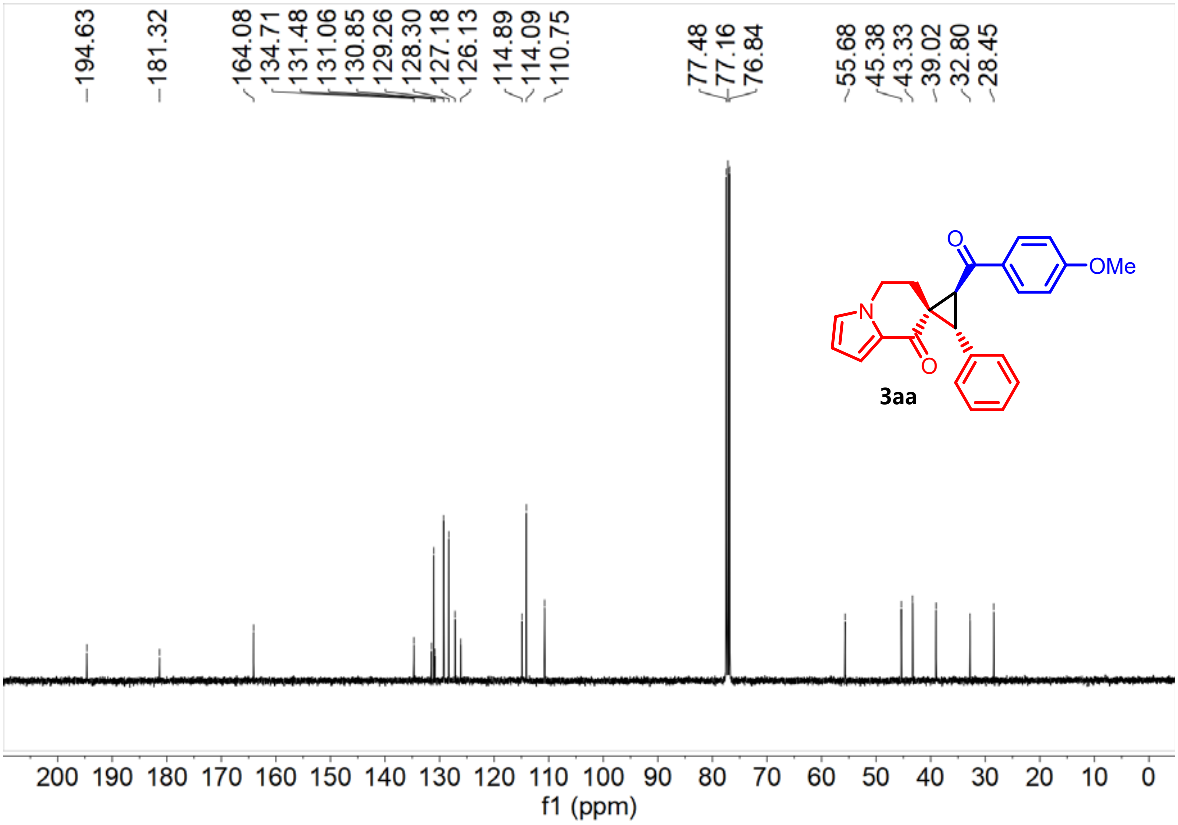


**Figure S2.** $^{13}$C-NMR of compound **3aa**

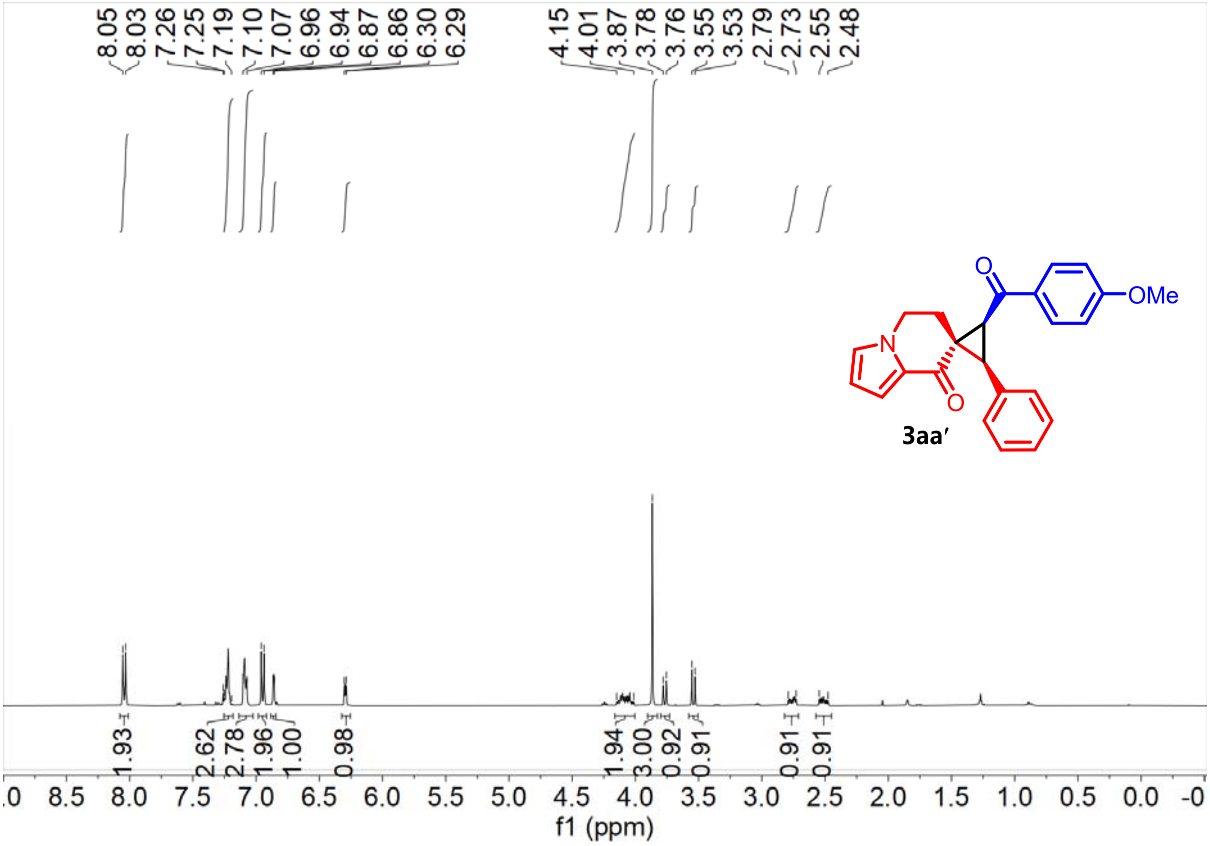


**Figure S3.** $^{1}$H-NMR of compound **3aa’**

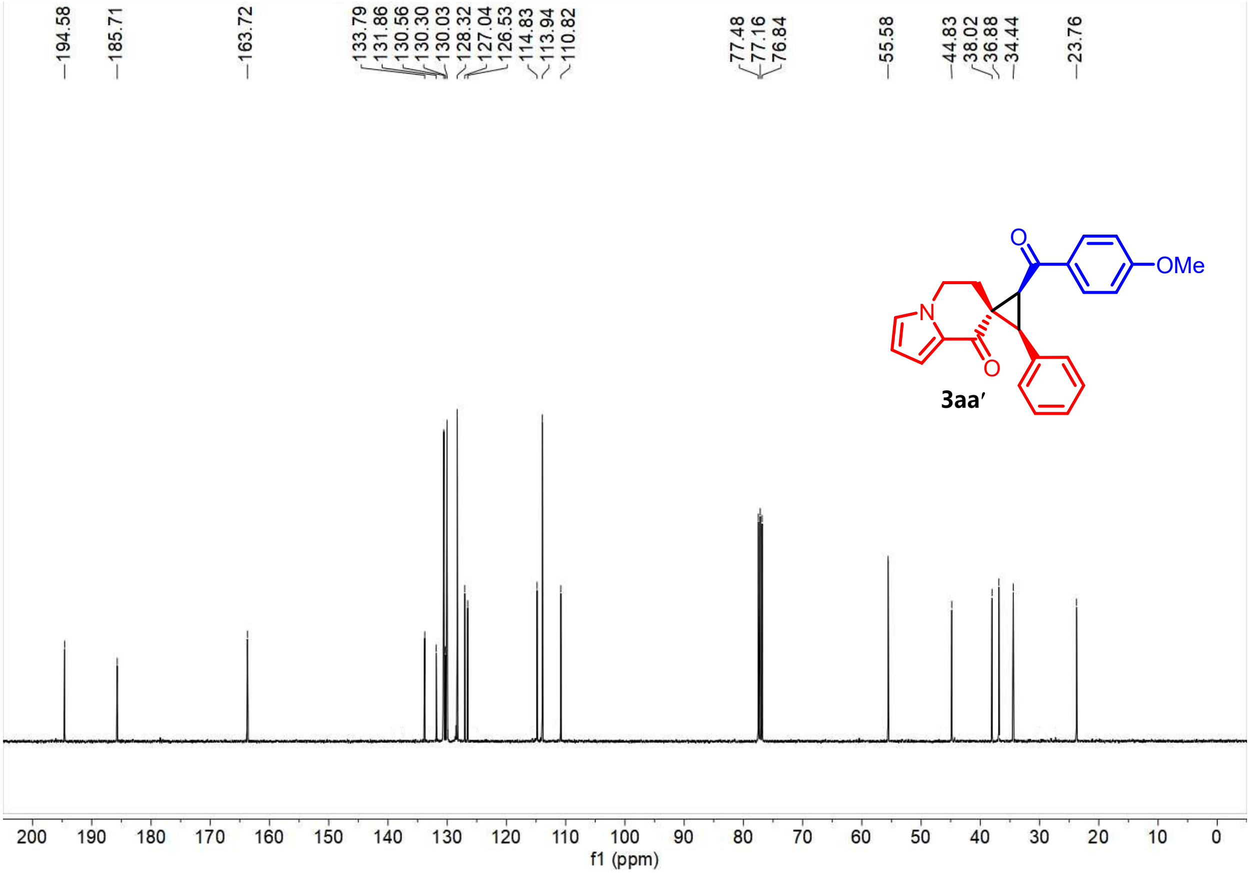


**Figure S4.** $^{13}$C-NMR of compound **3aa’**

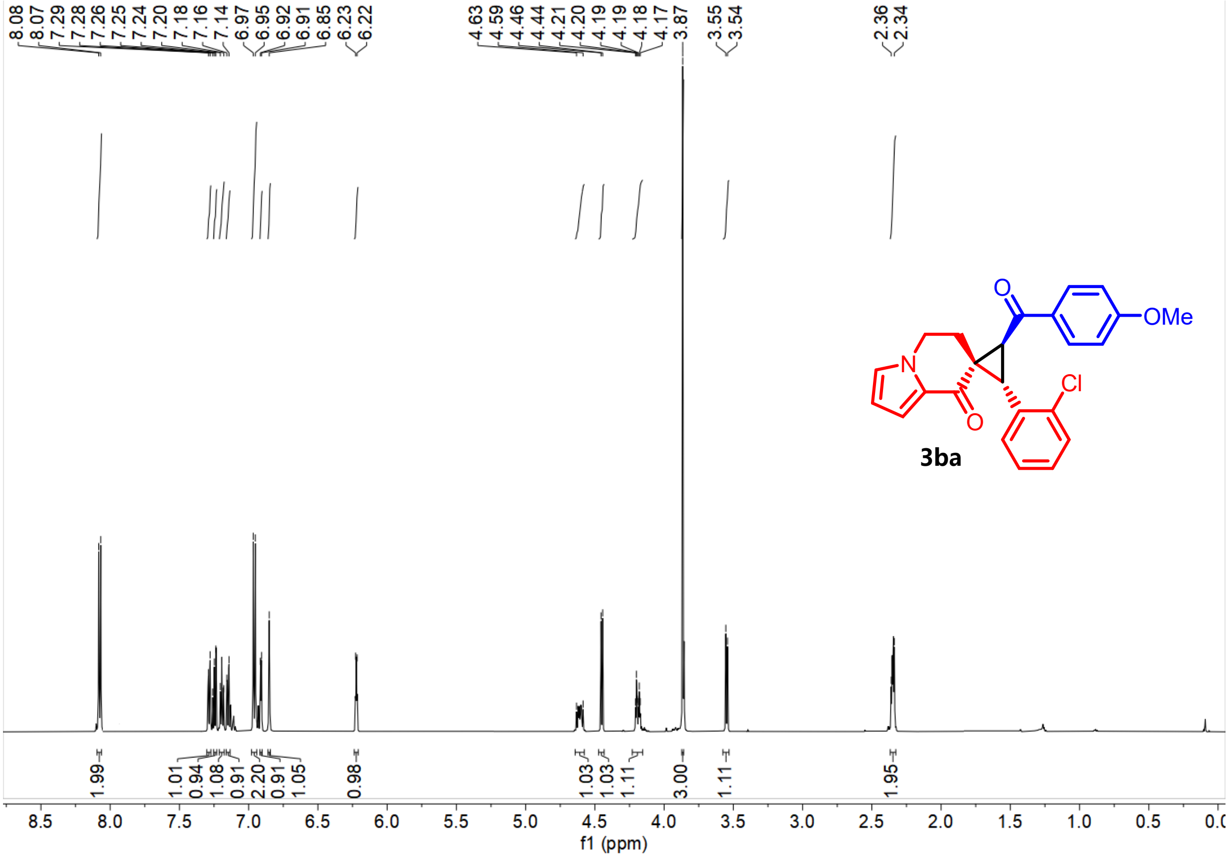


**Figure S5.** $^{1}H$-NMR of compound **3ba**

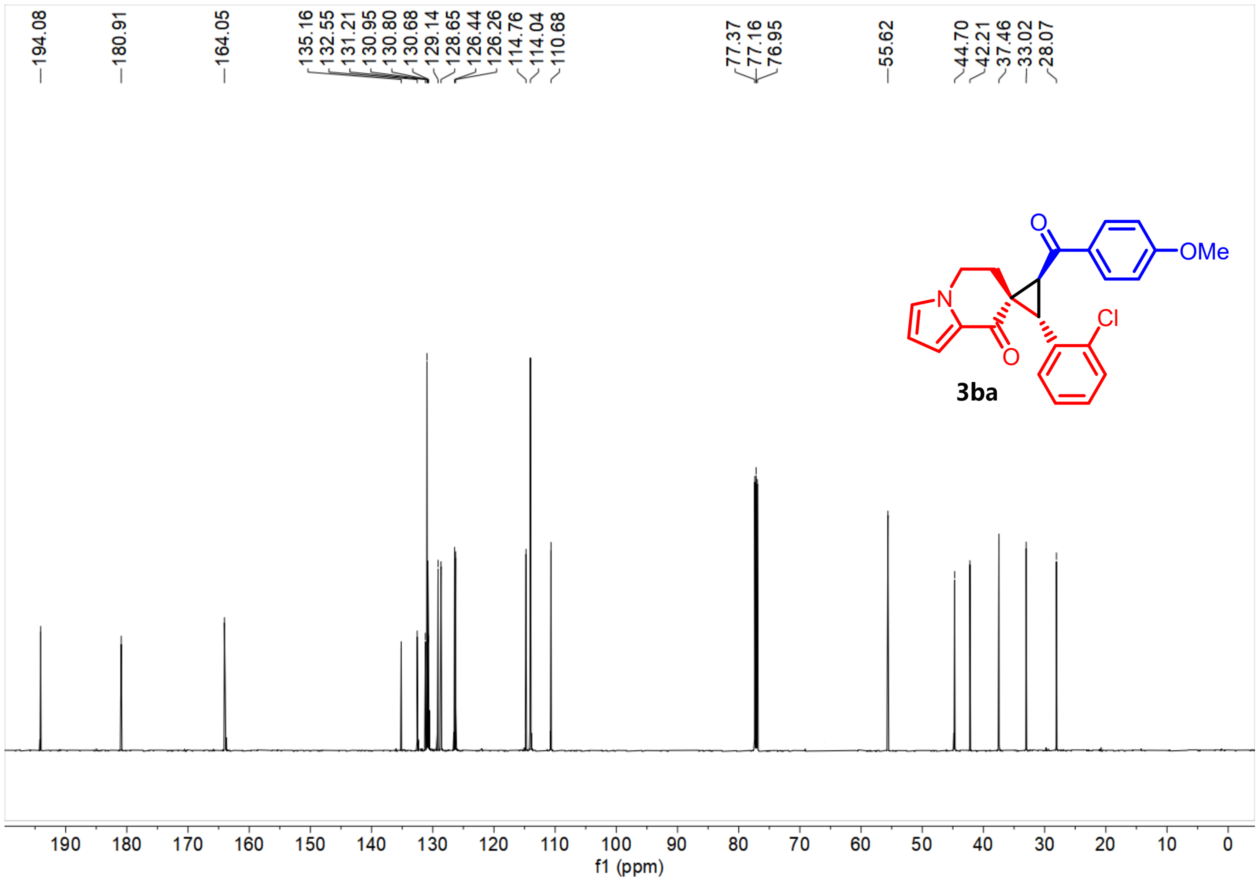


**Figure S6.** $^{13}C$-NMR of compound **3ba**

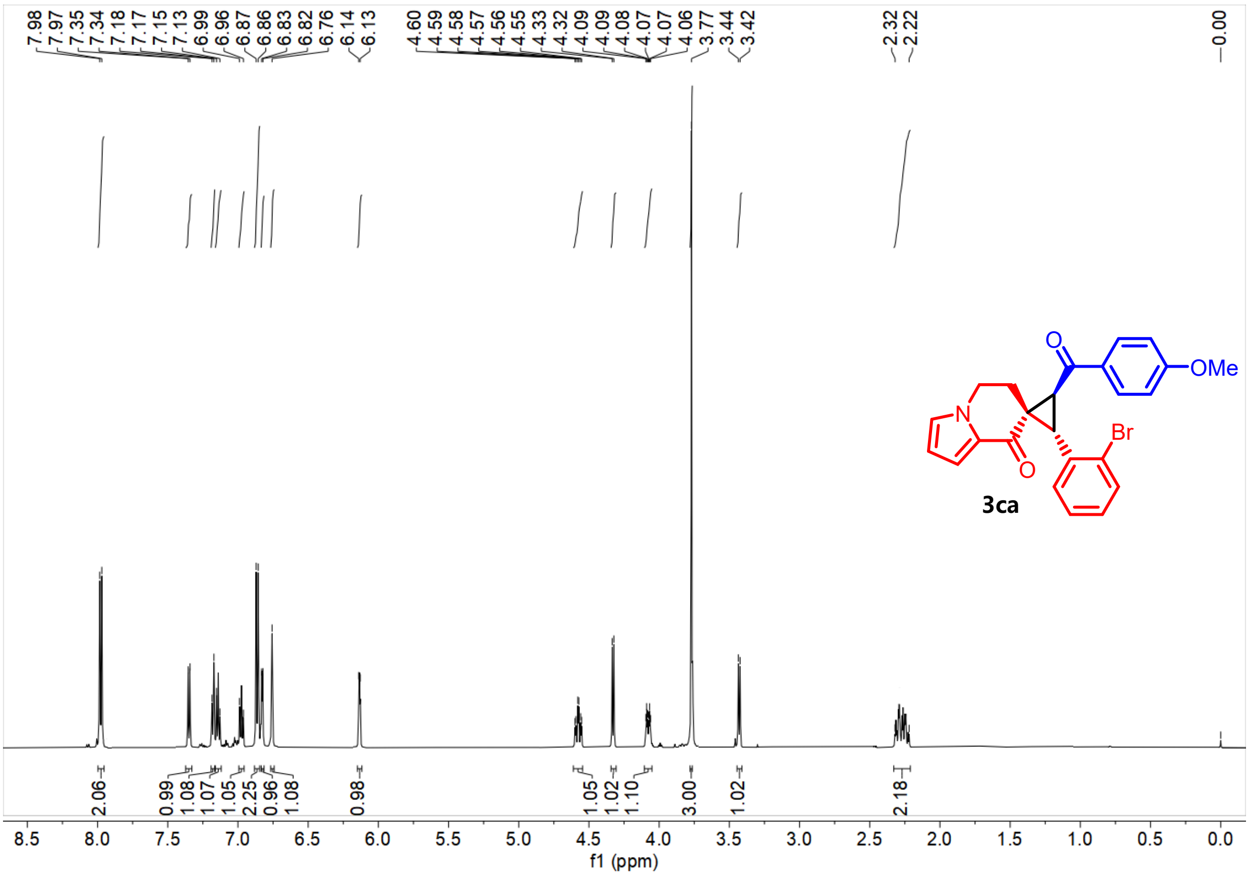

**Figure S7.** $^{1}$H-NMR of compound **3ca**

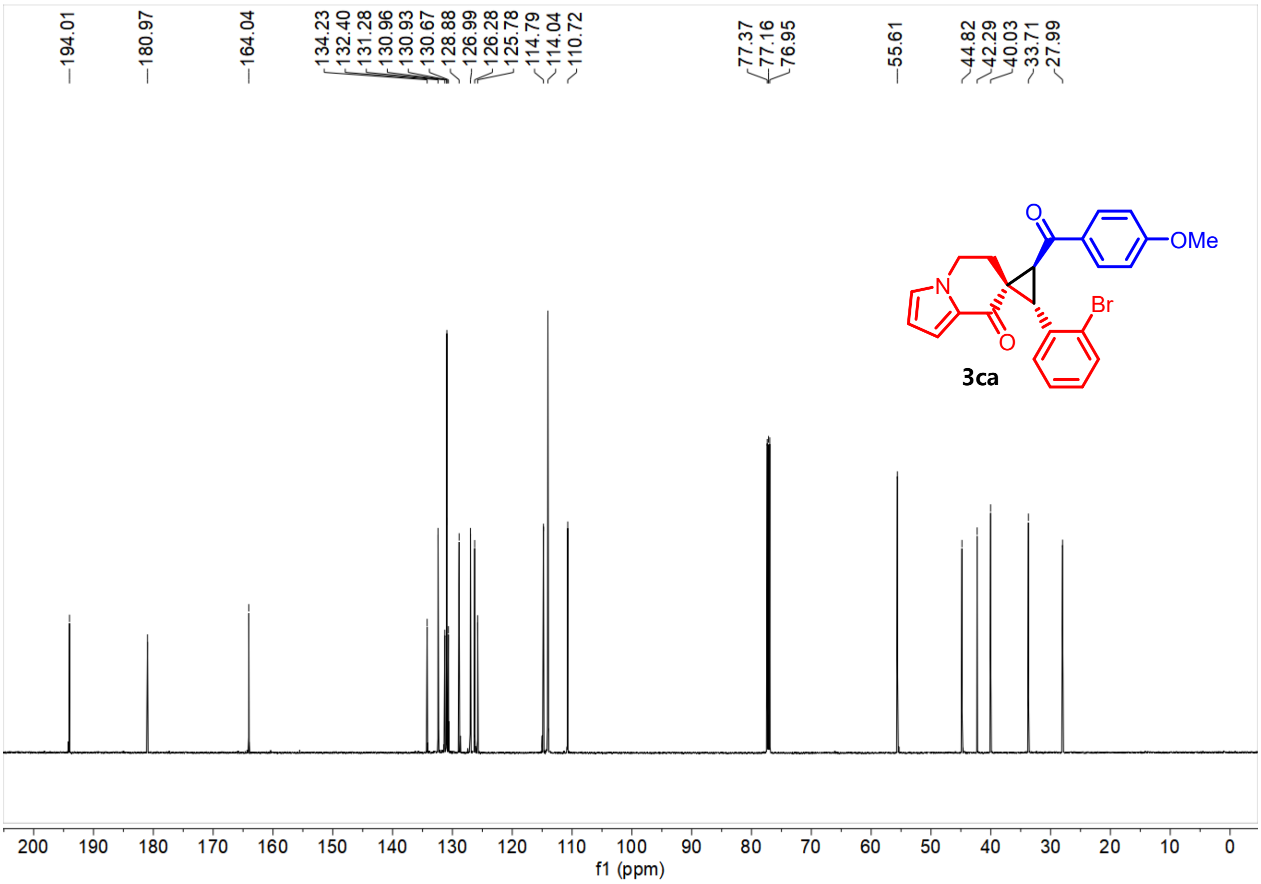

**Figure S8.** $^{13}$C-NMR of compound **3ca**

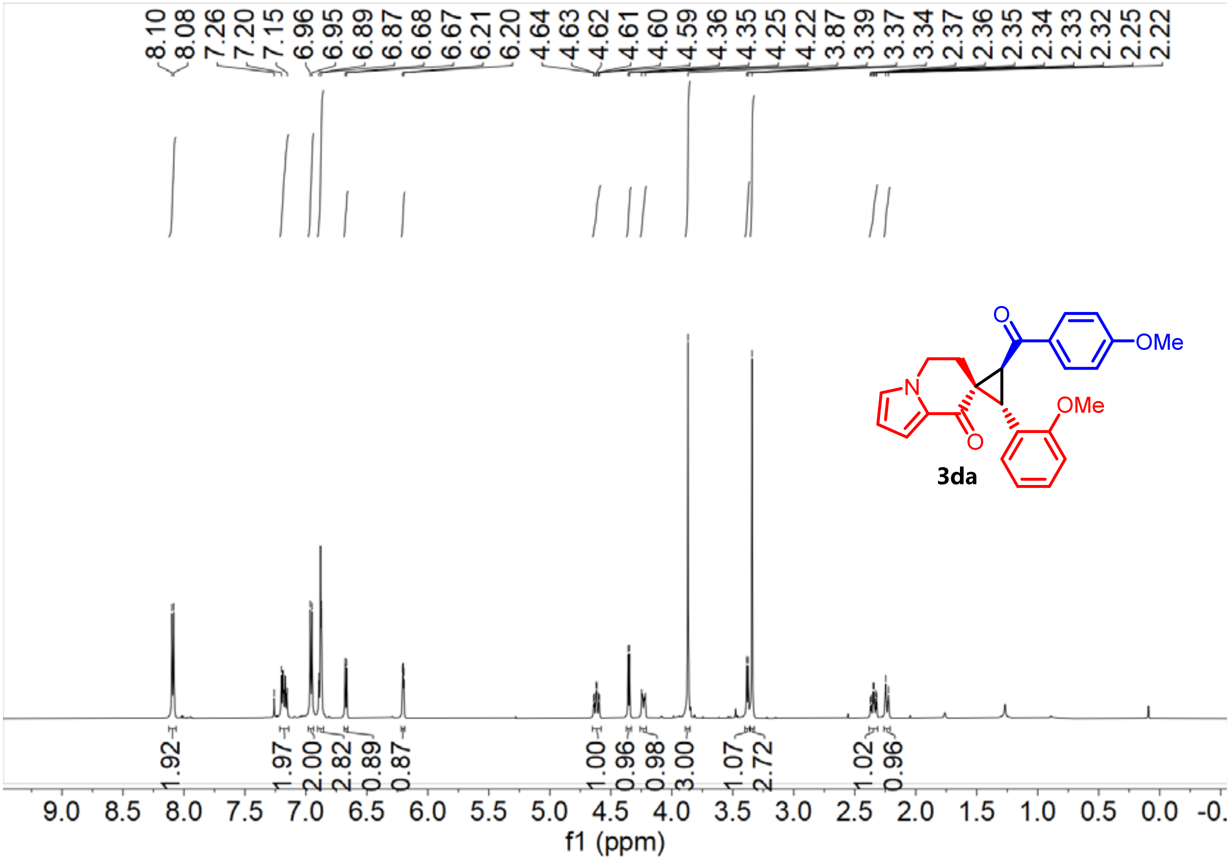


**Figure S9.** $^{1}$H-NMR of compound **3da**

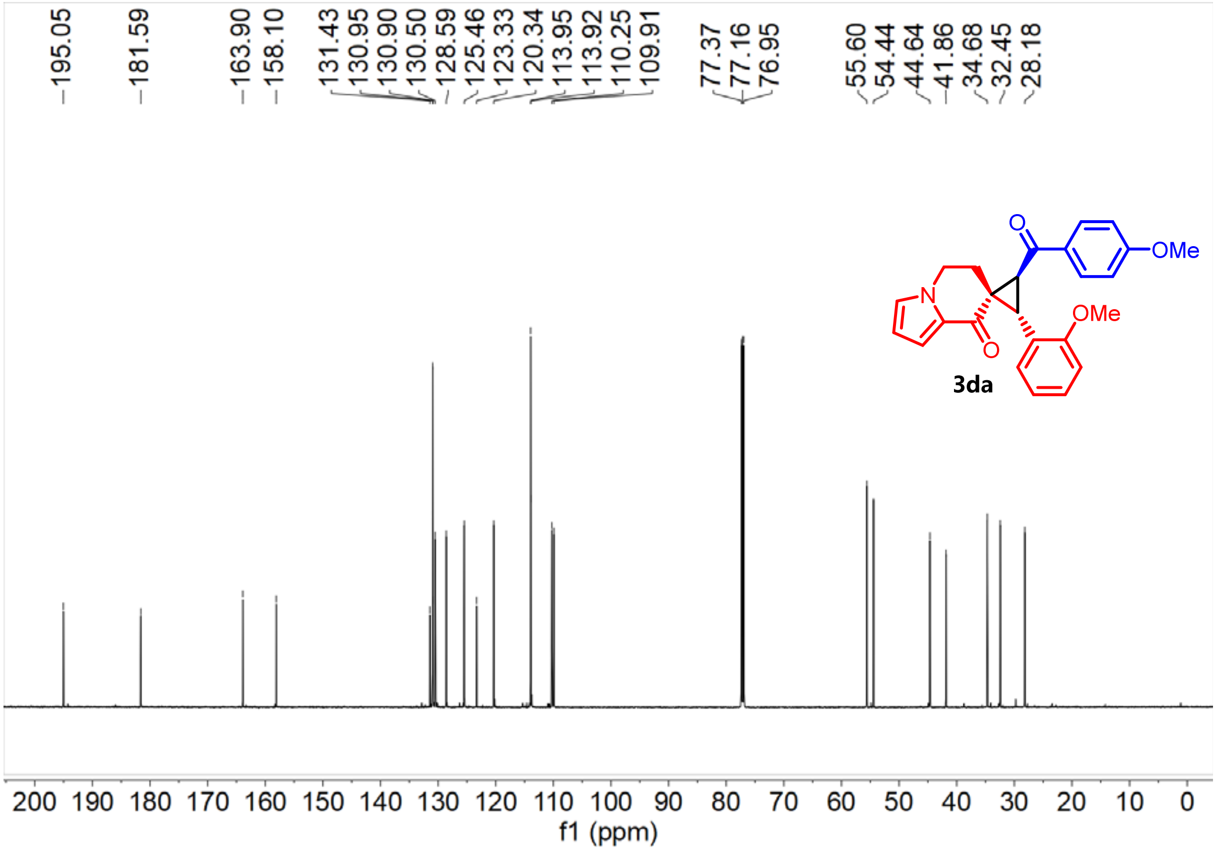


**Figure S10.** $^{13}$C-NMR of compound **3da**

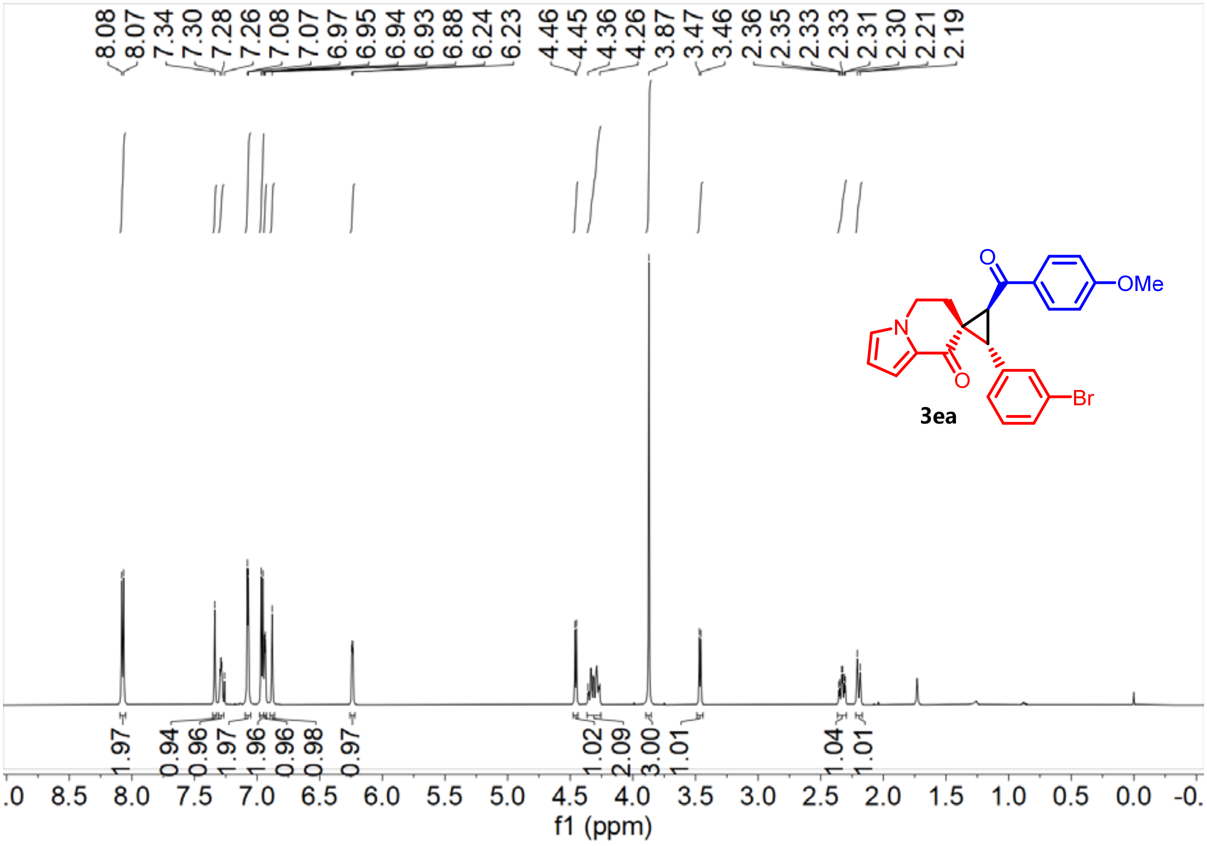

**Figure S11.** $^{1}$H-NMR of compound **3ea**

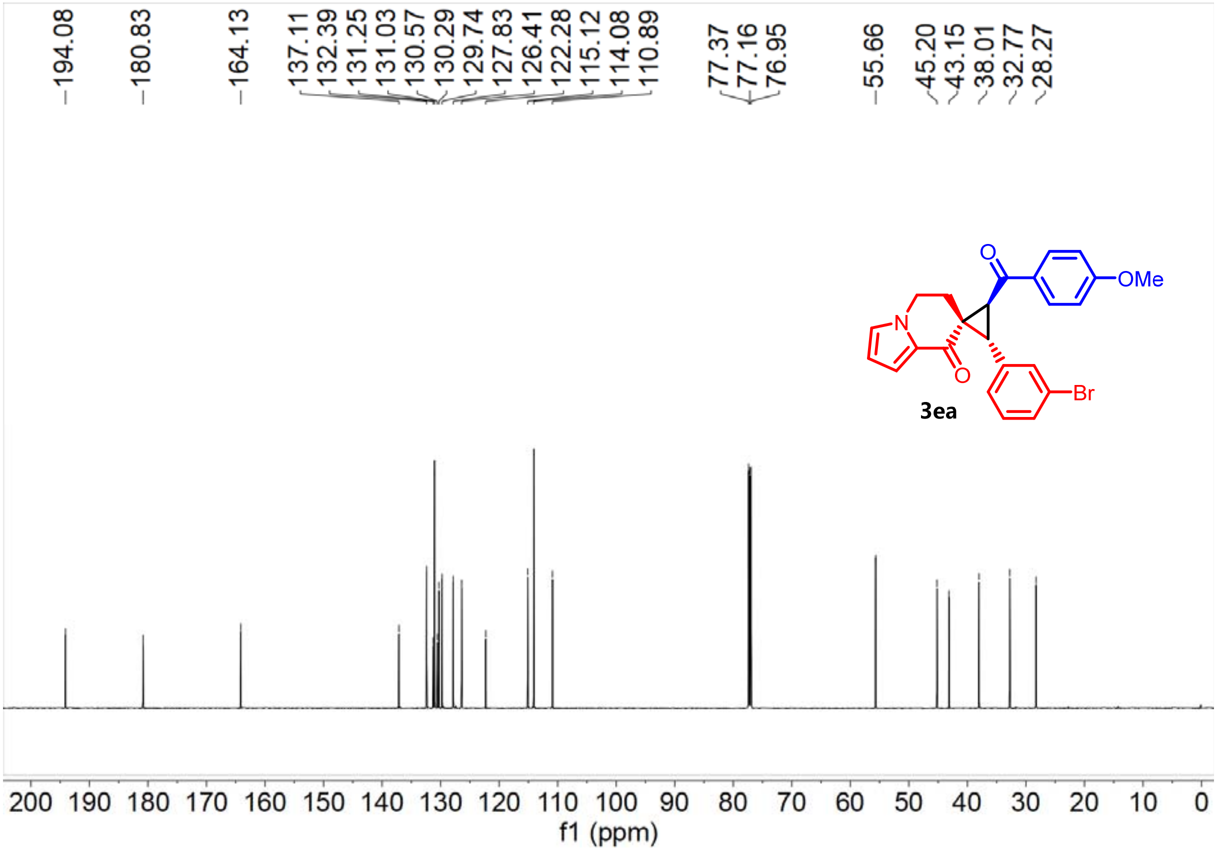

**Figure S12.** $^{13}$C-NMR of compound **3ea**

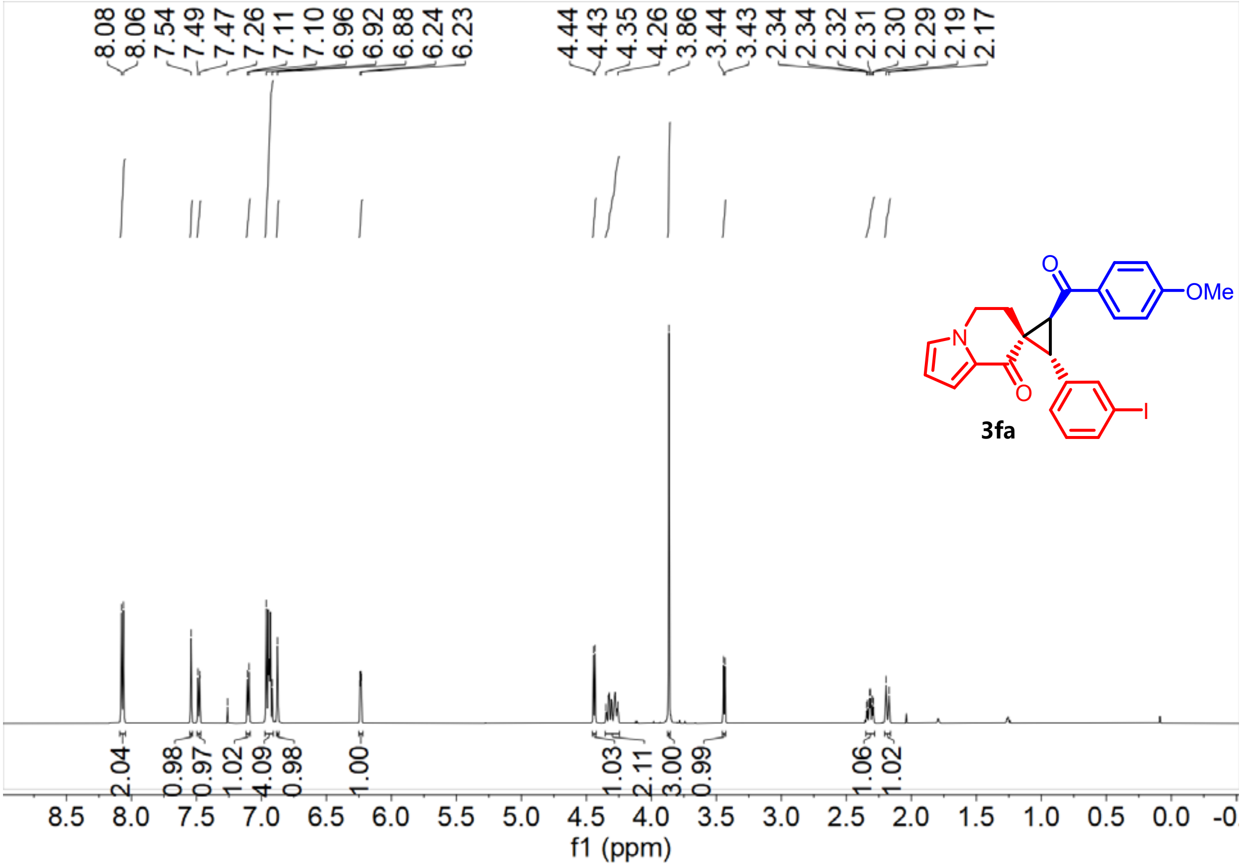


**Figure S13.** $^{1}$H-NMR of compound **3fa**

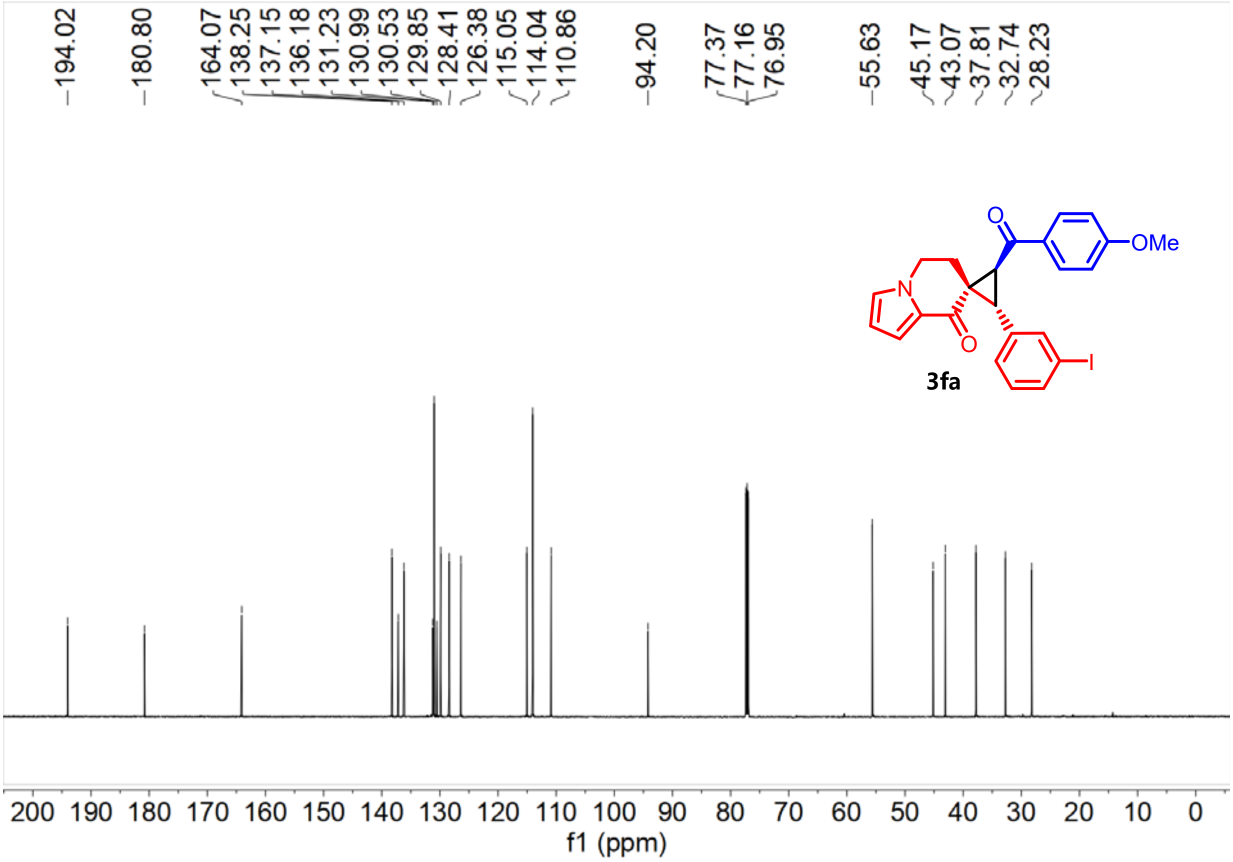


**Figure S14.** $^{13}$C-NMR of compound **3fa**

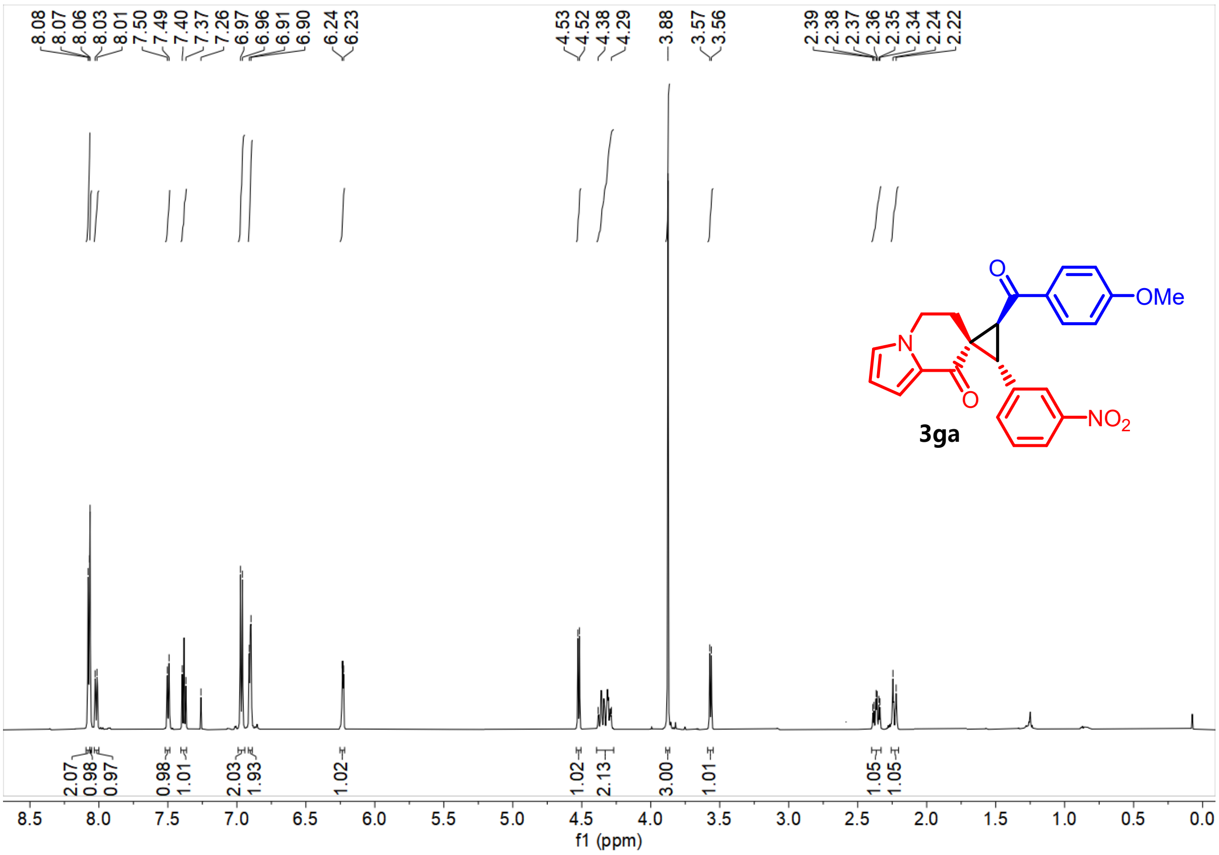


**Figure S15.** $^{1}H$-NMR of compound **3ga**

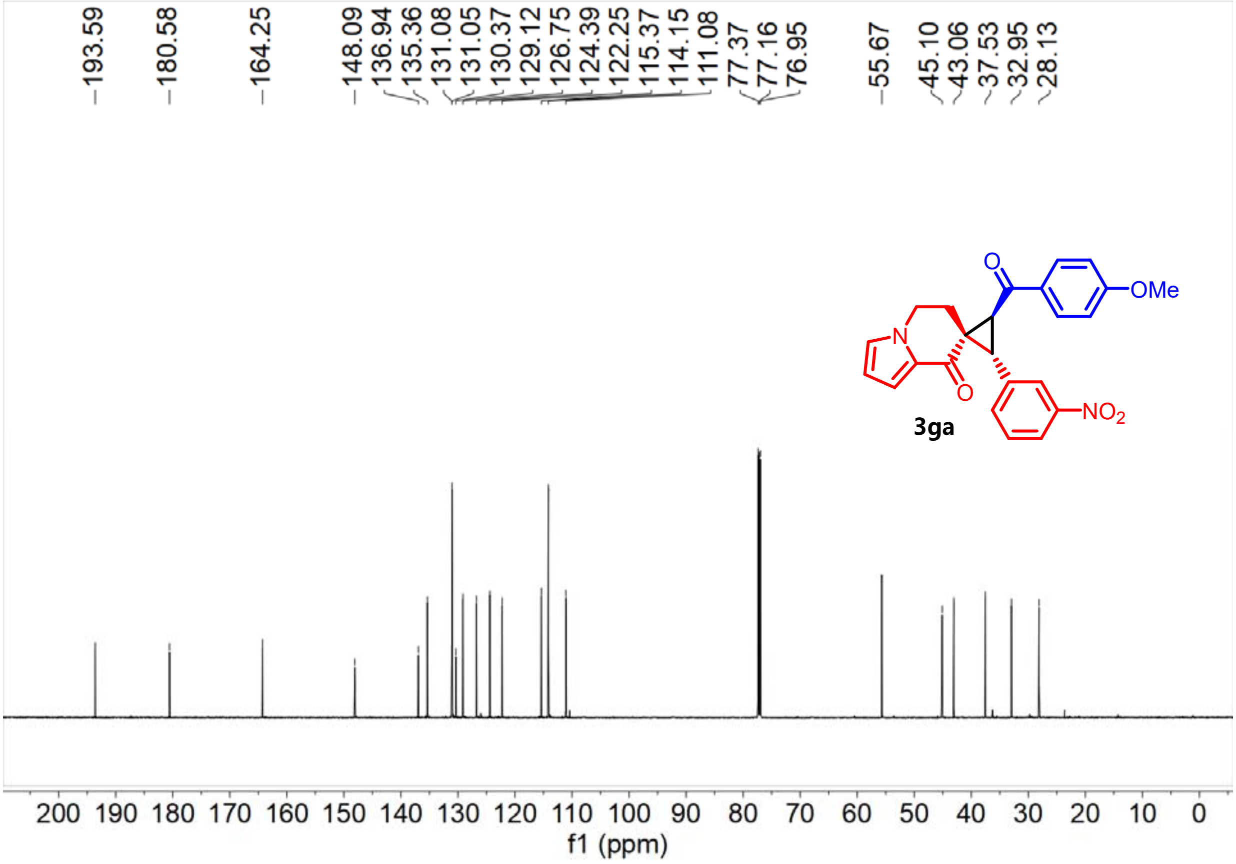


**Figure S16.** $^{13}C$-NMR of compound **3ga**

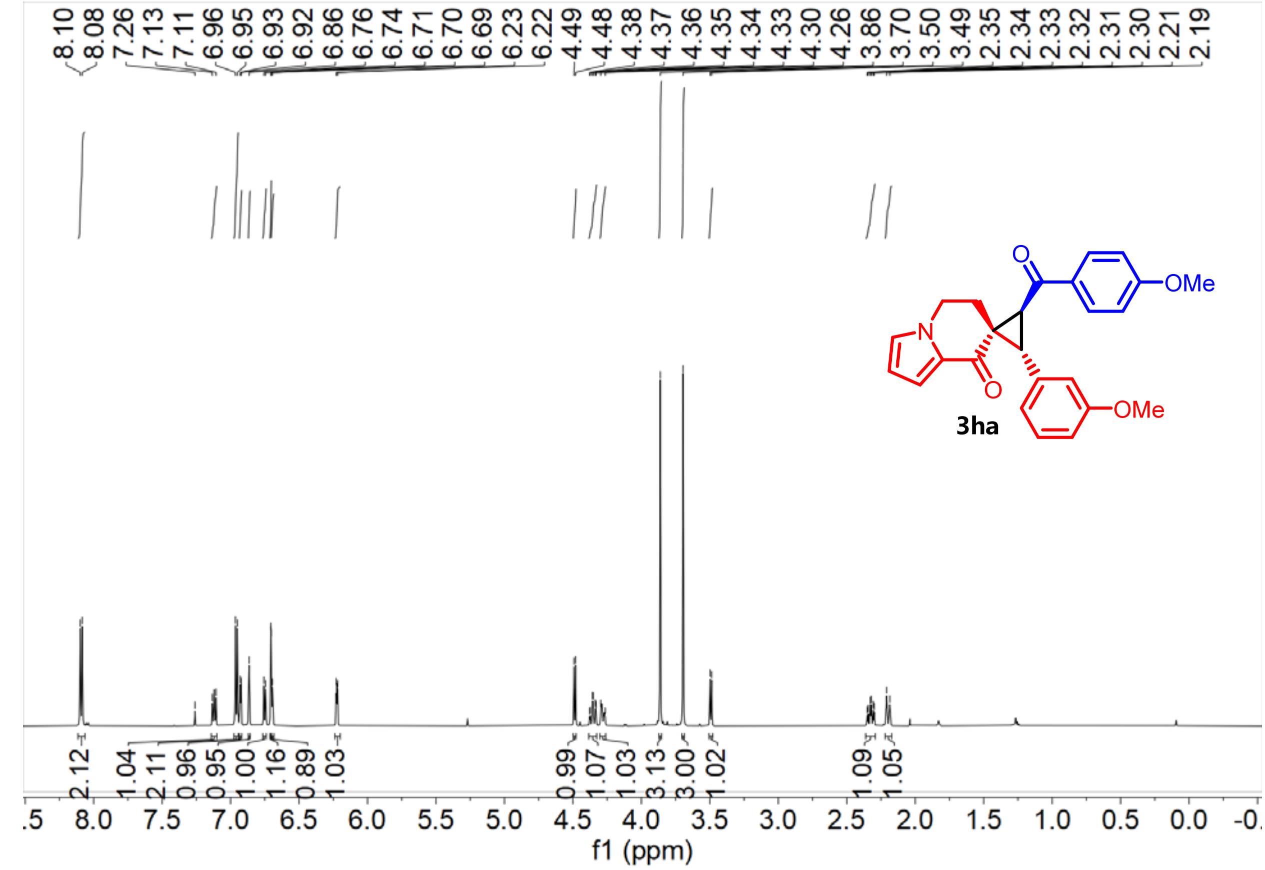


**Figure S17.** $^{1}$H-NMR of compound **3ha**

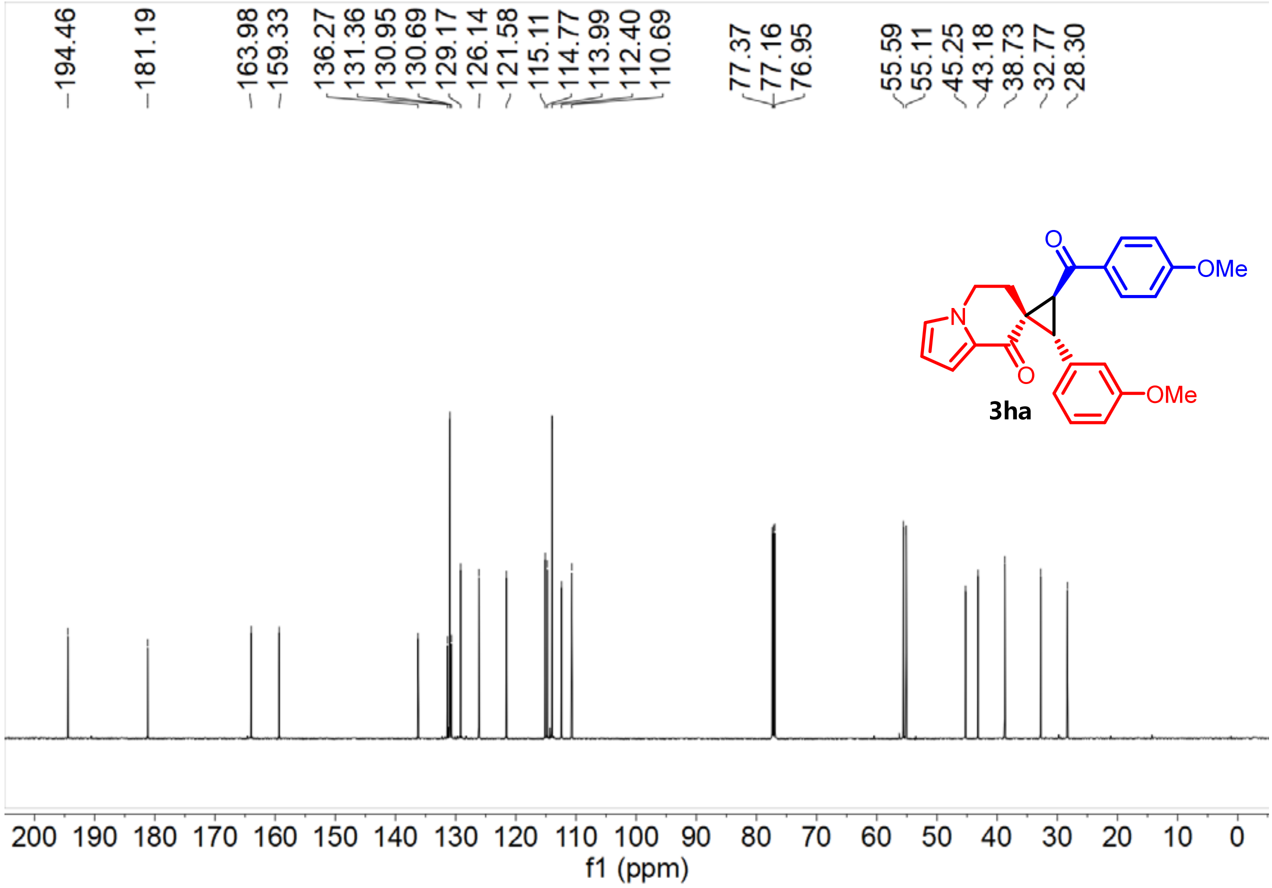


**Figure S18.** $^{13}$C-NMR of compound **3ha**

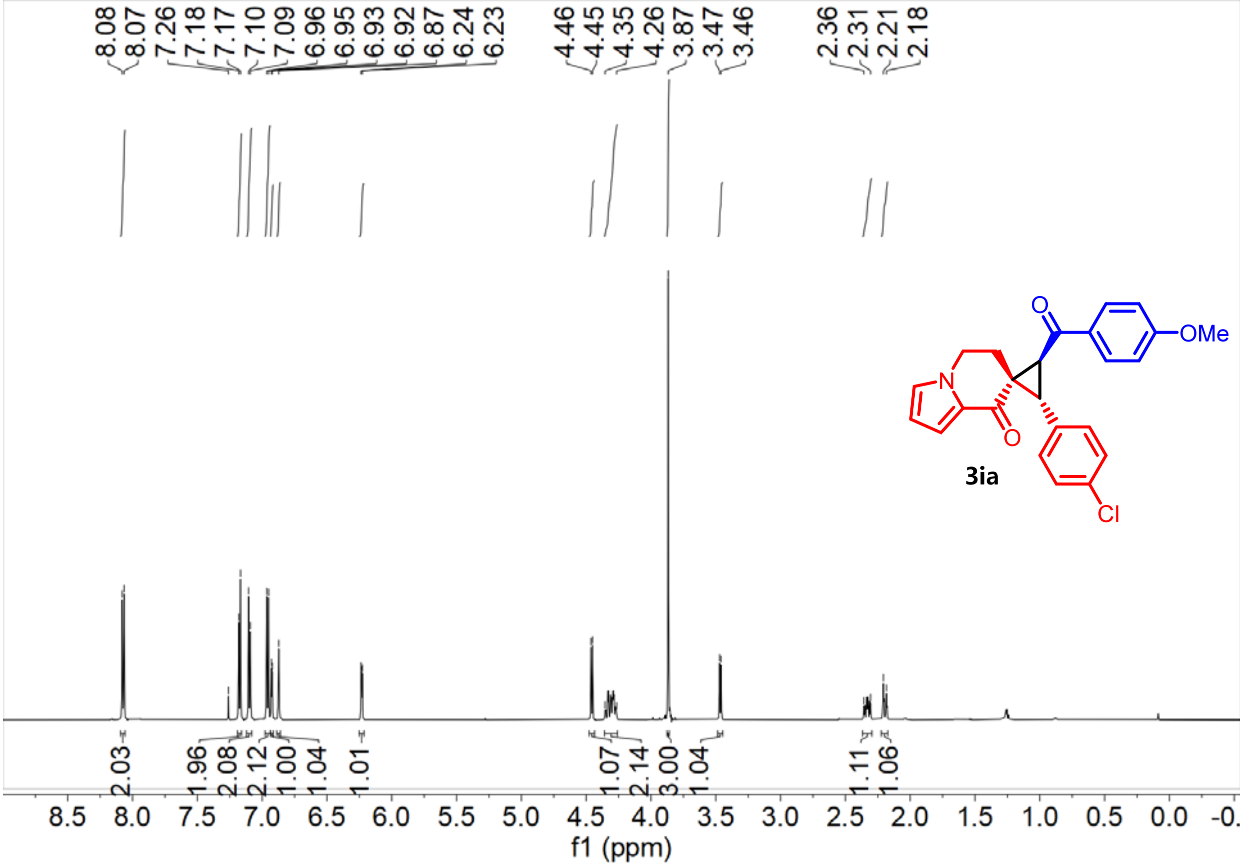


**Figure S19.** $^{1}$H-NMR of compound **3ia**

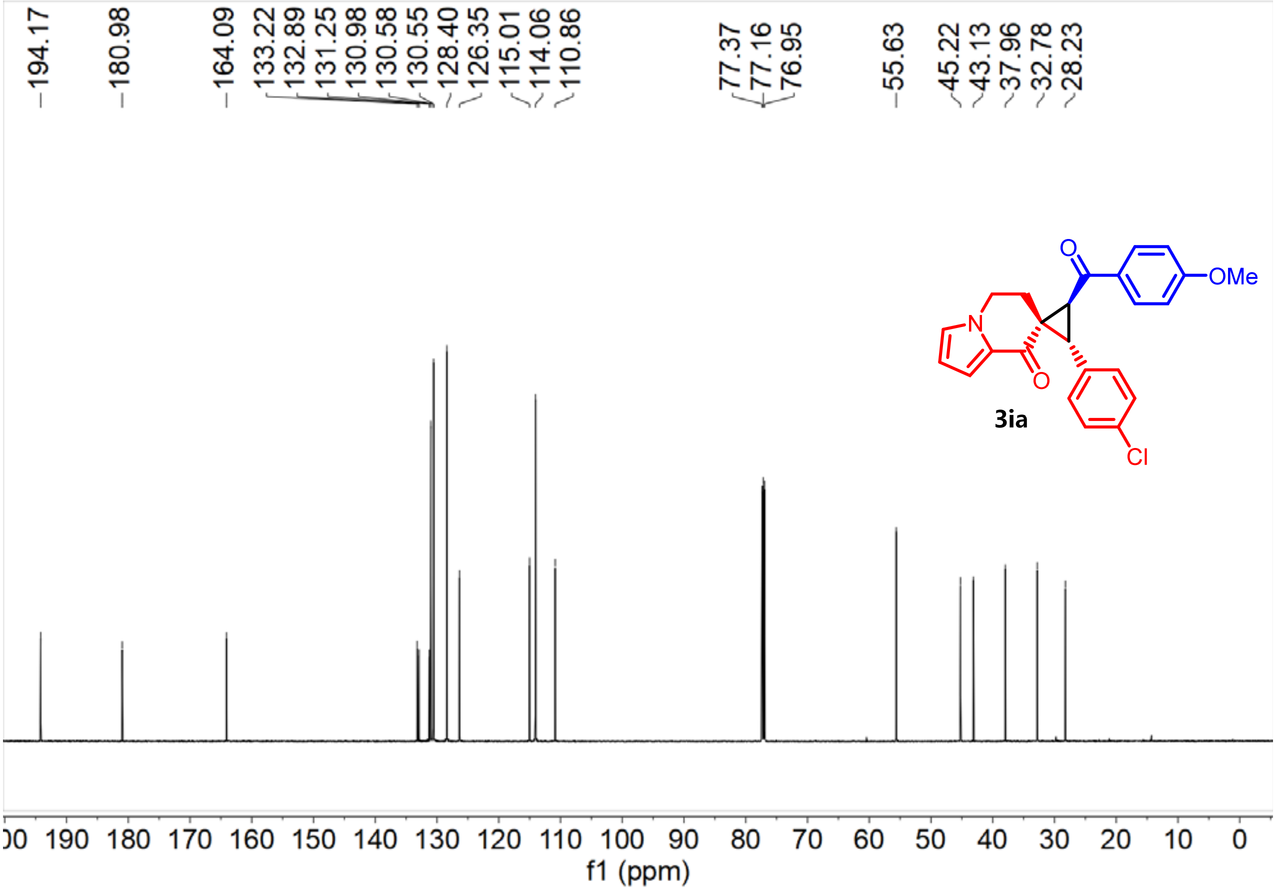


**Figure S20.** $^{13}$C-NMR of compound **3ia**

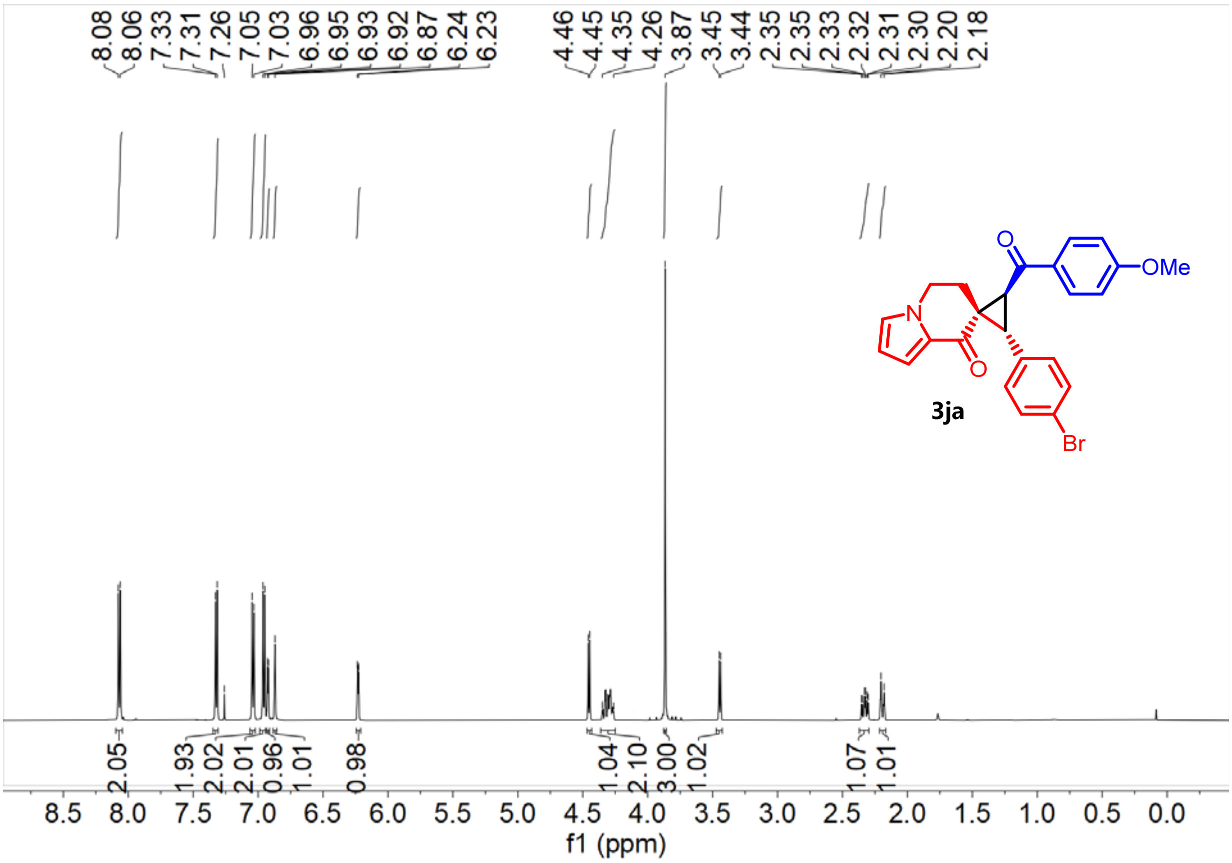


**Figure S21.** $^1$H-NMR of compound **3ja**

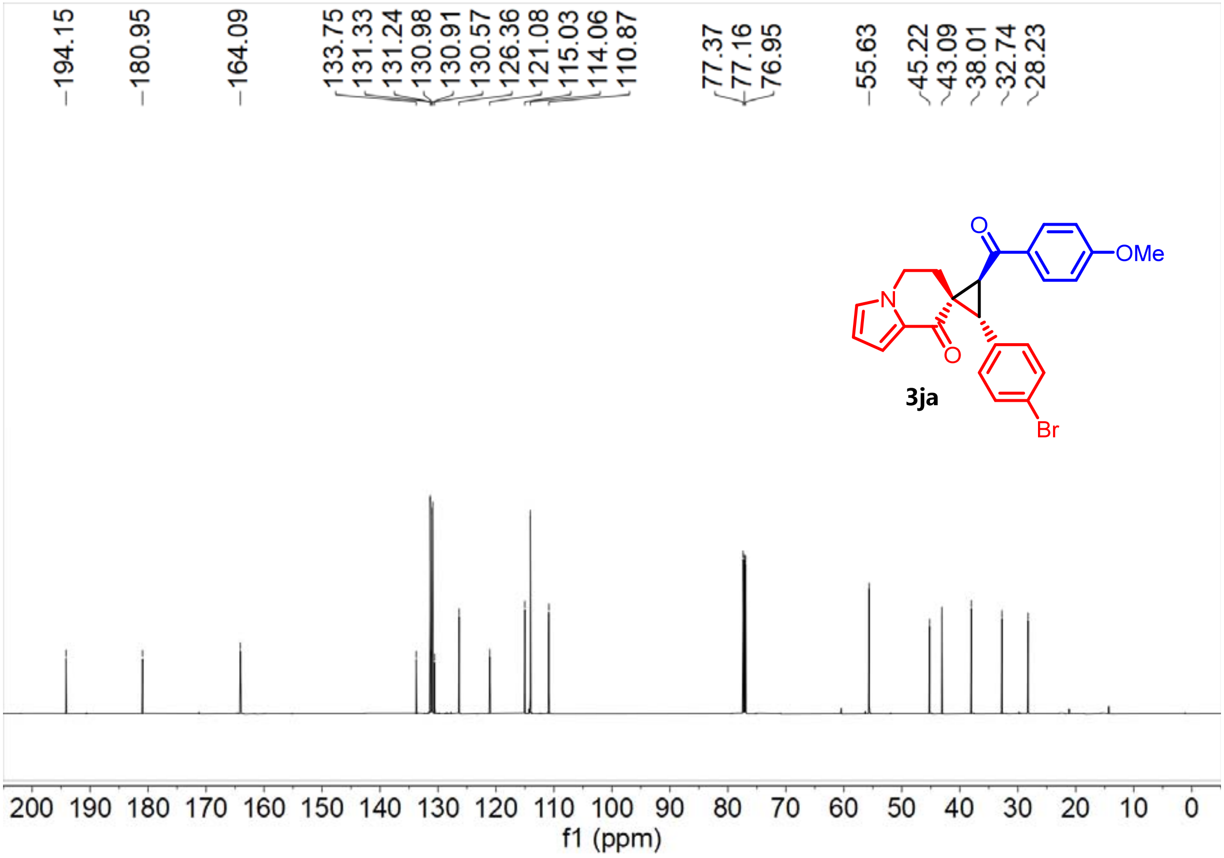


**Figure S22.** $^{13}$C-NMR of compound **3ja**

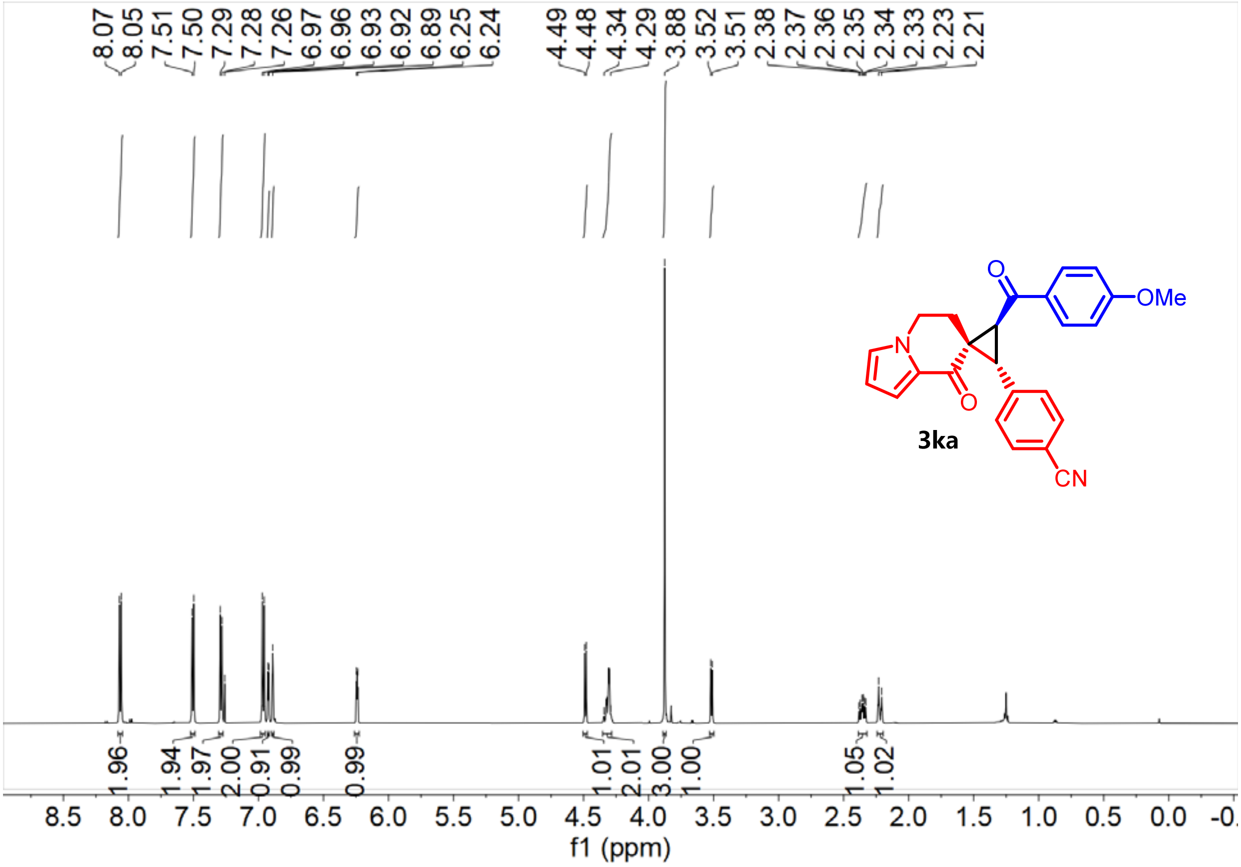


**Figure S23.** $^{1}H$-NMR of compound **3ka**

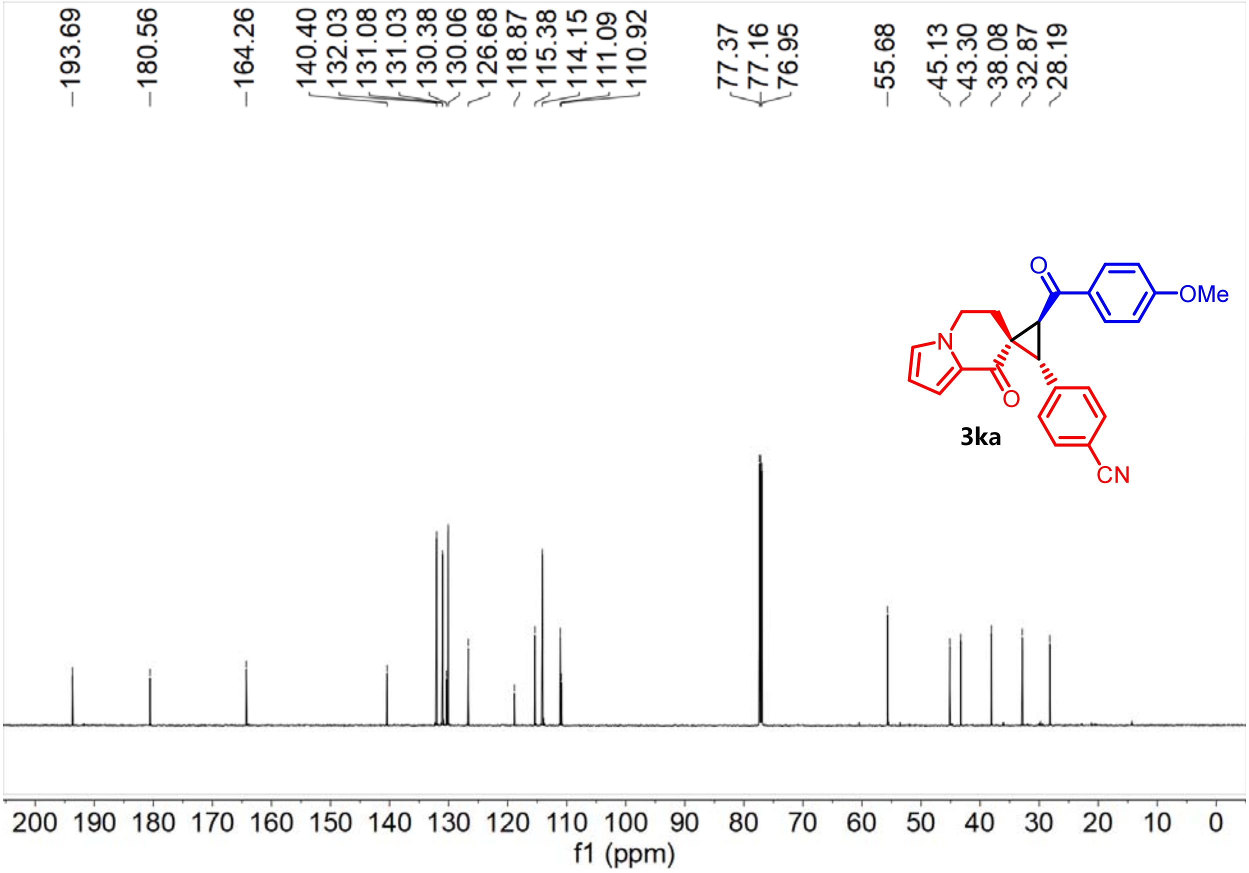


**Figure S24.** $^{13}C$-NMR of compound **3ka**

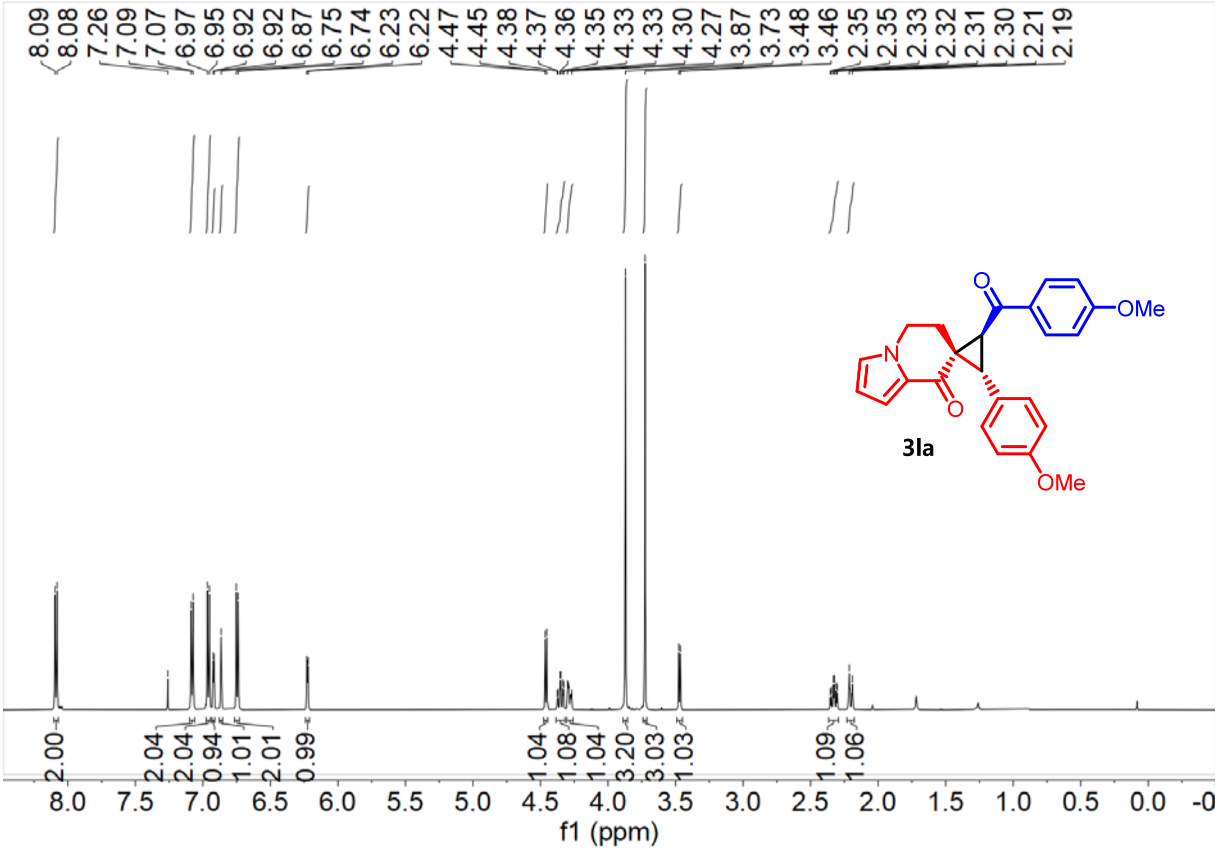

**Figure S25.** $^{1}H$-NMR of compound **3la**

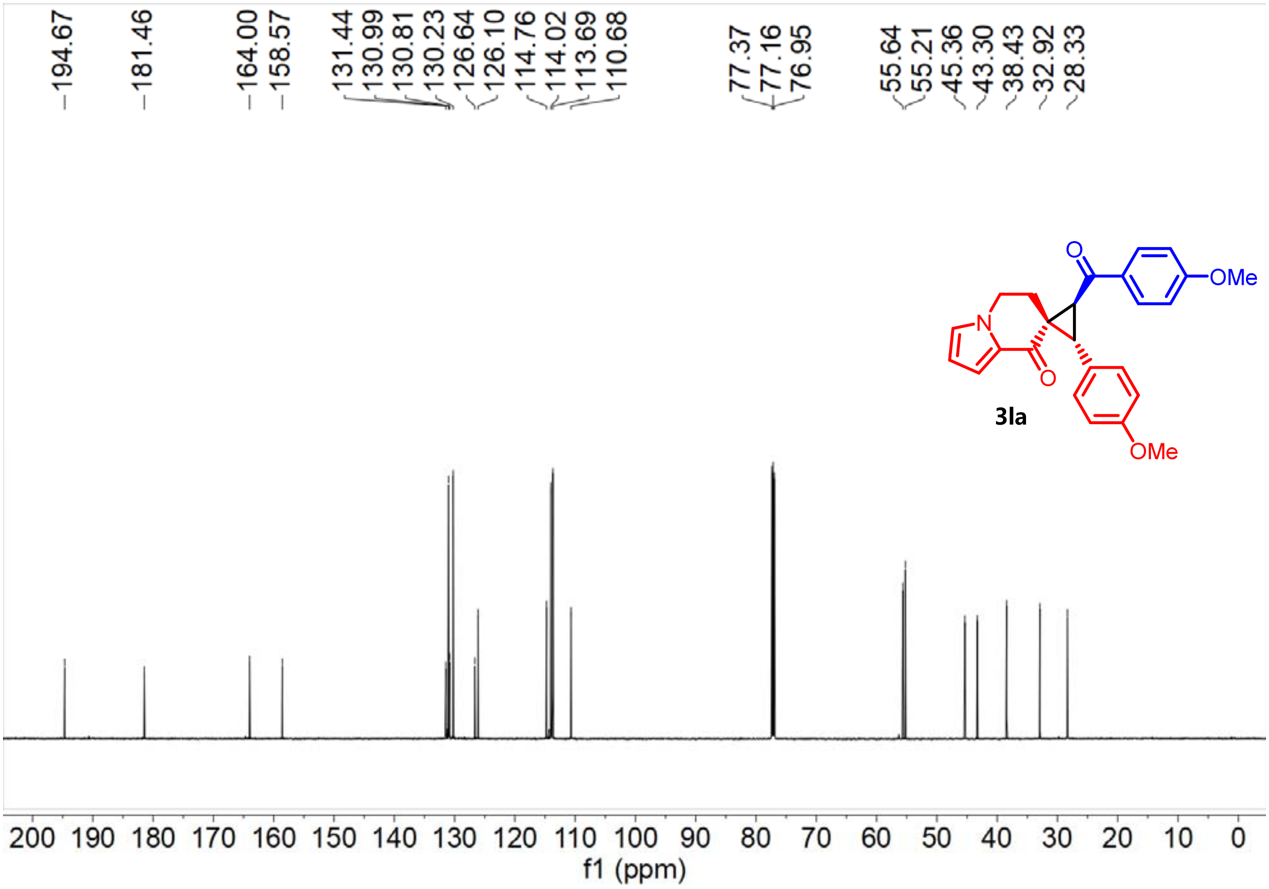

**Figure S26.** $^{13}C$-NMR of compound **3la**

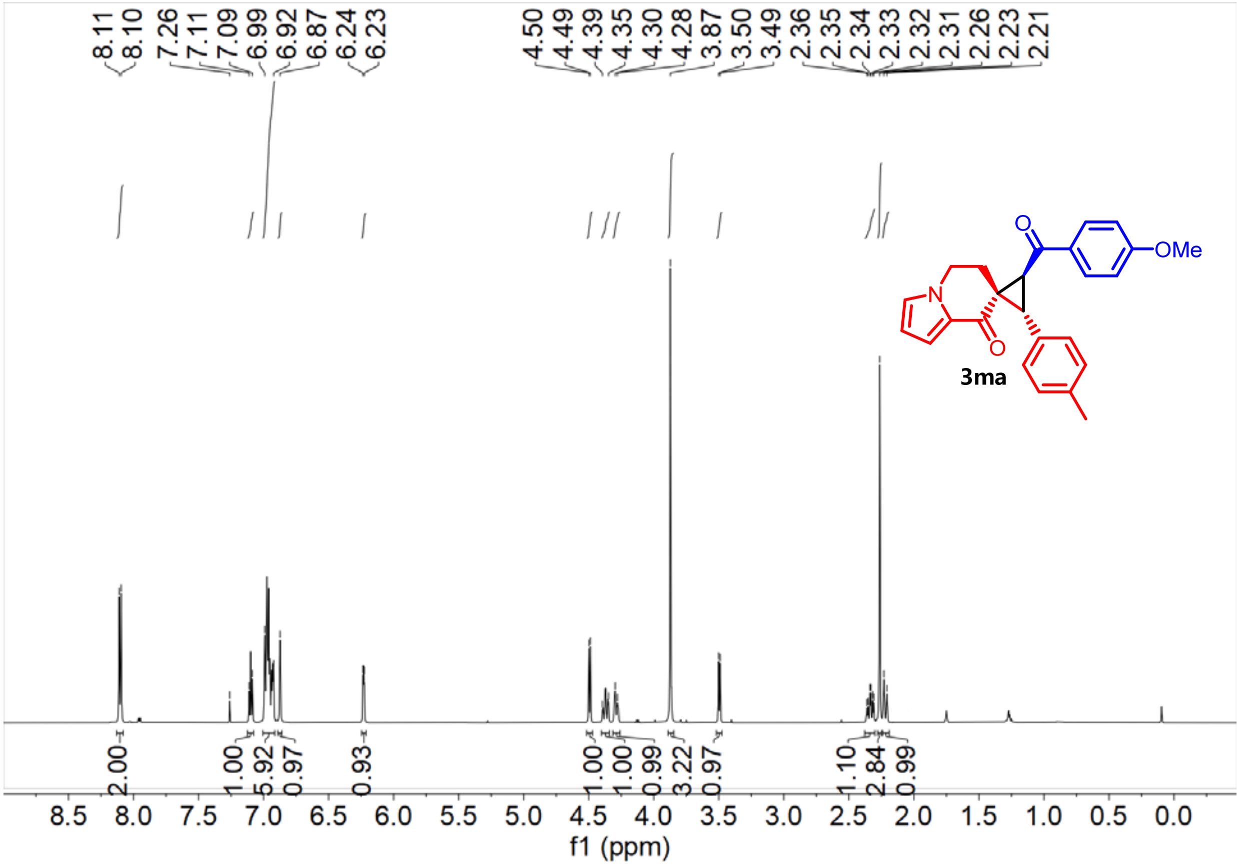

**Figure S27.** $^{1}$H-NMR of compound **3ma**

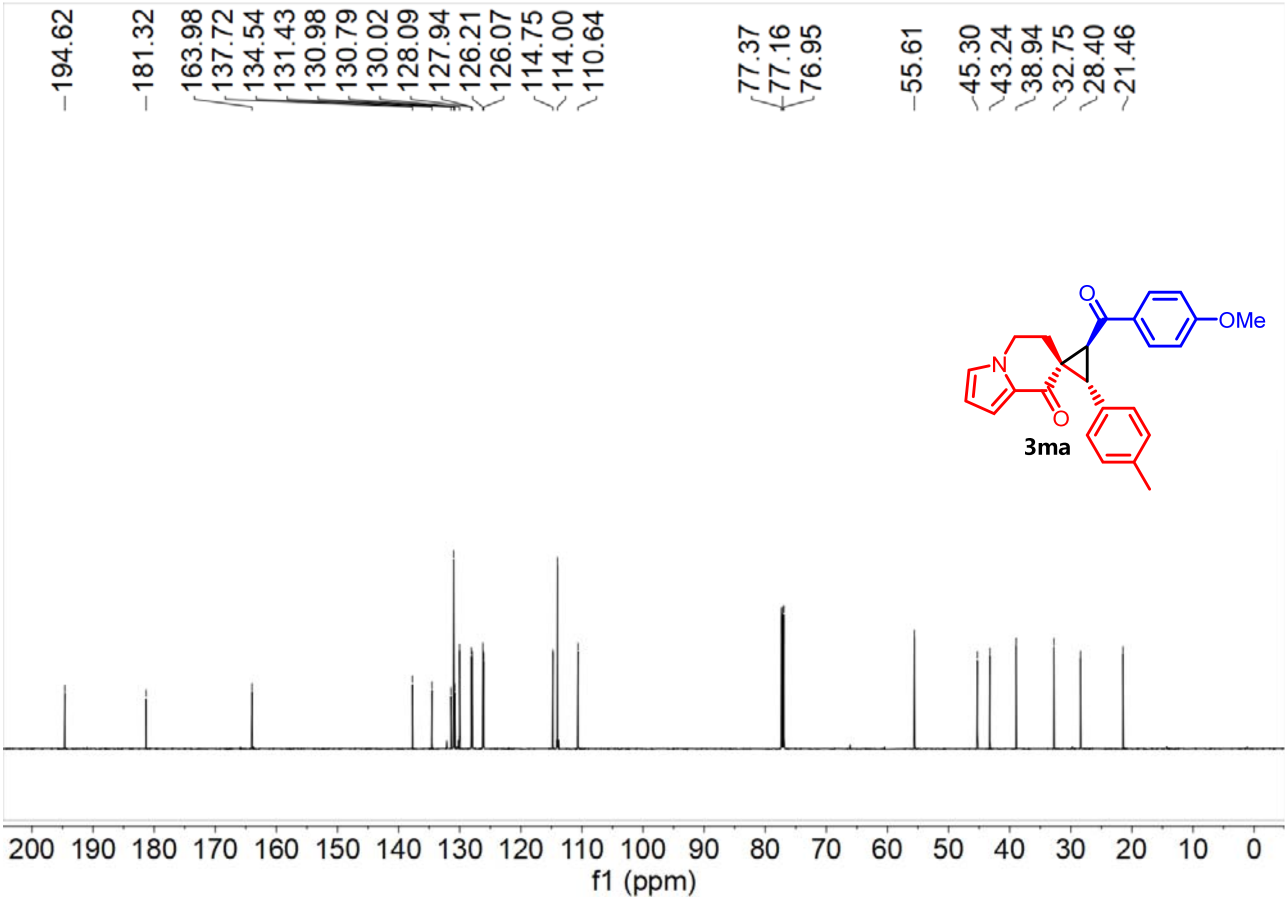

**Figure S28.** $^{13}$C-NMR of compound **3ma**

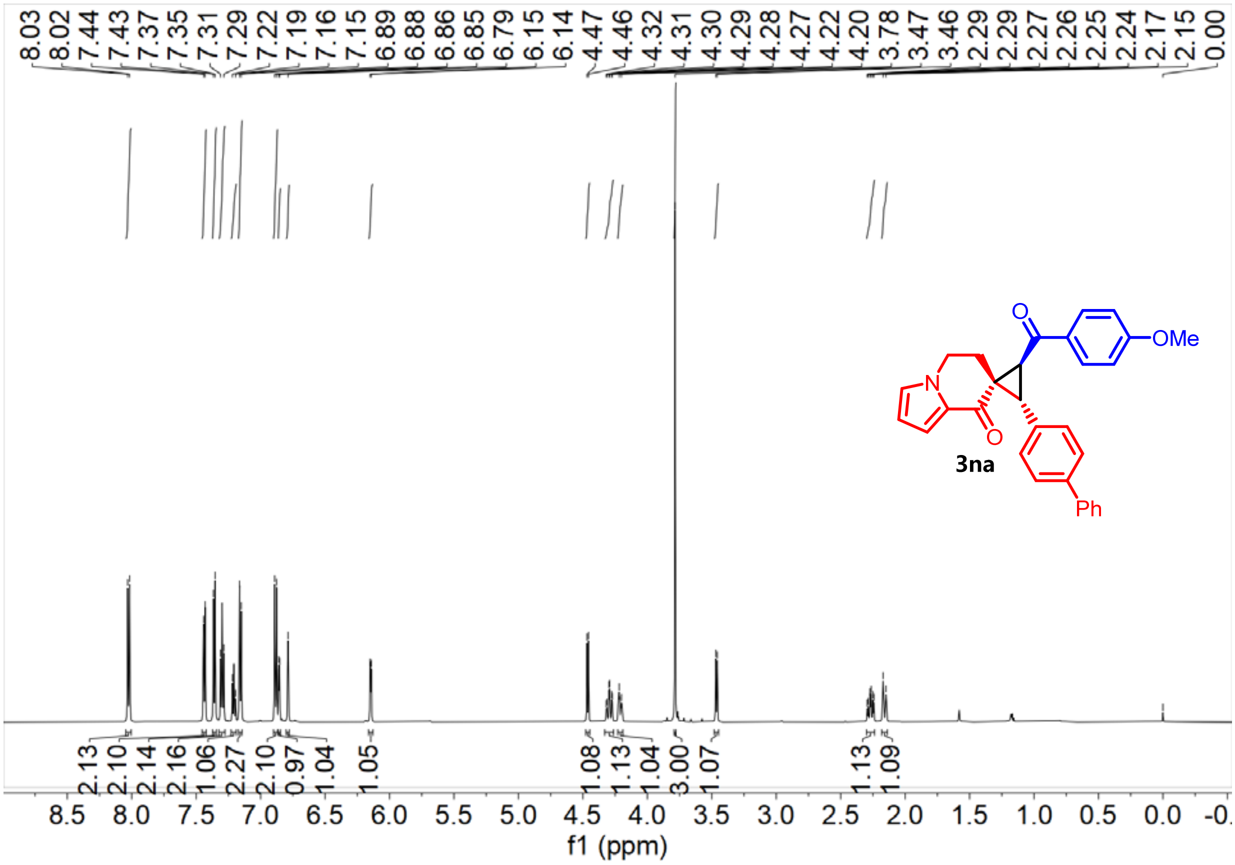

**Figure S29.** $^{1}$H-NMR of compound **3na**

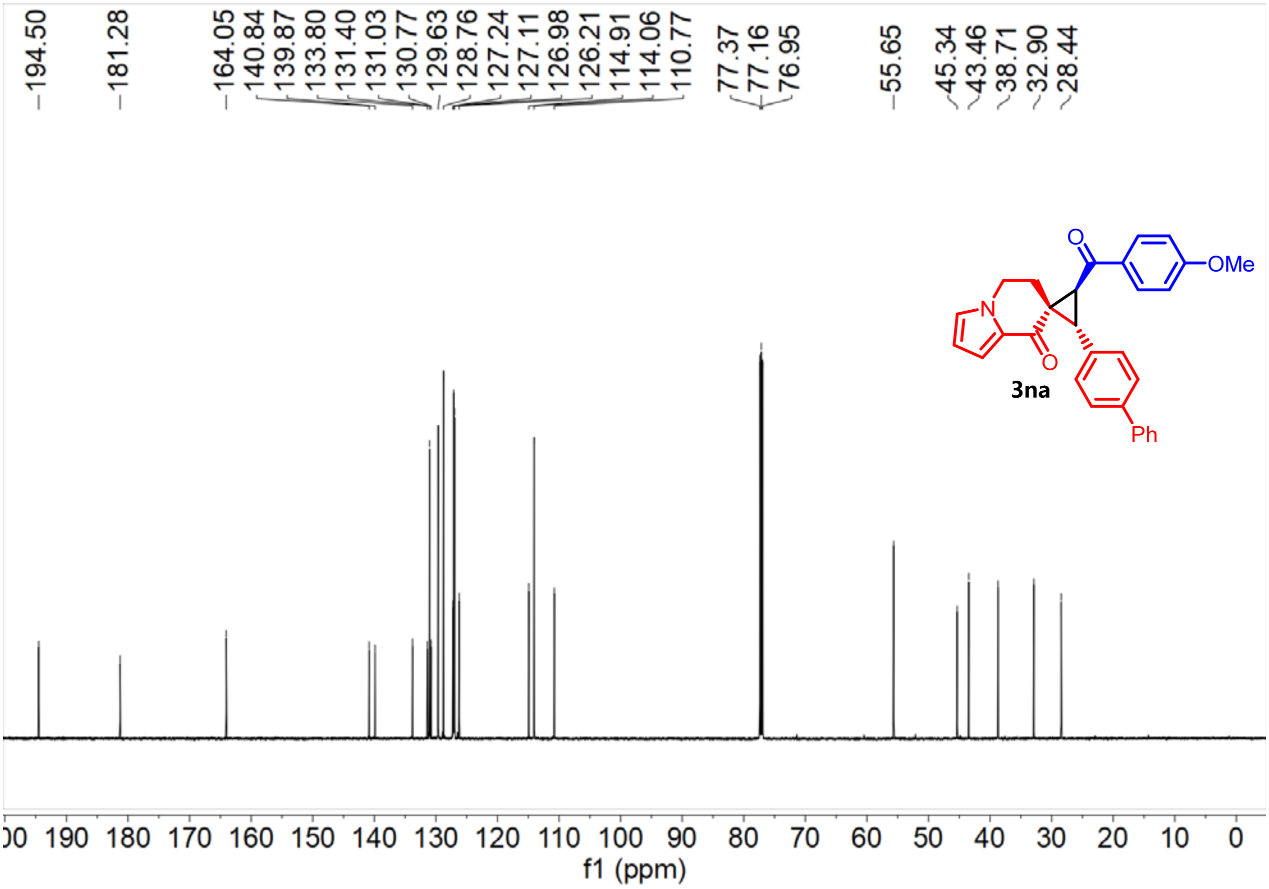

**Figure S30.** $^{13}$C-NMR of compound **3na**

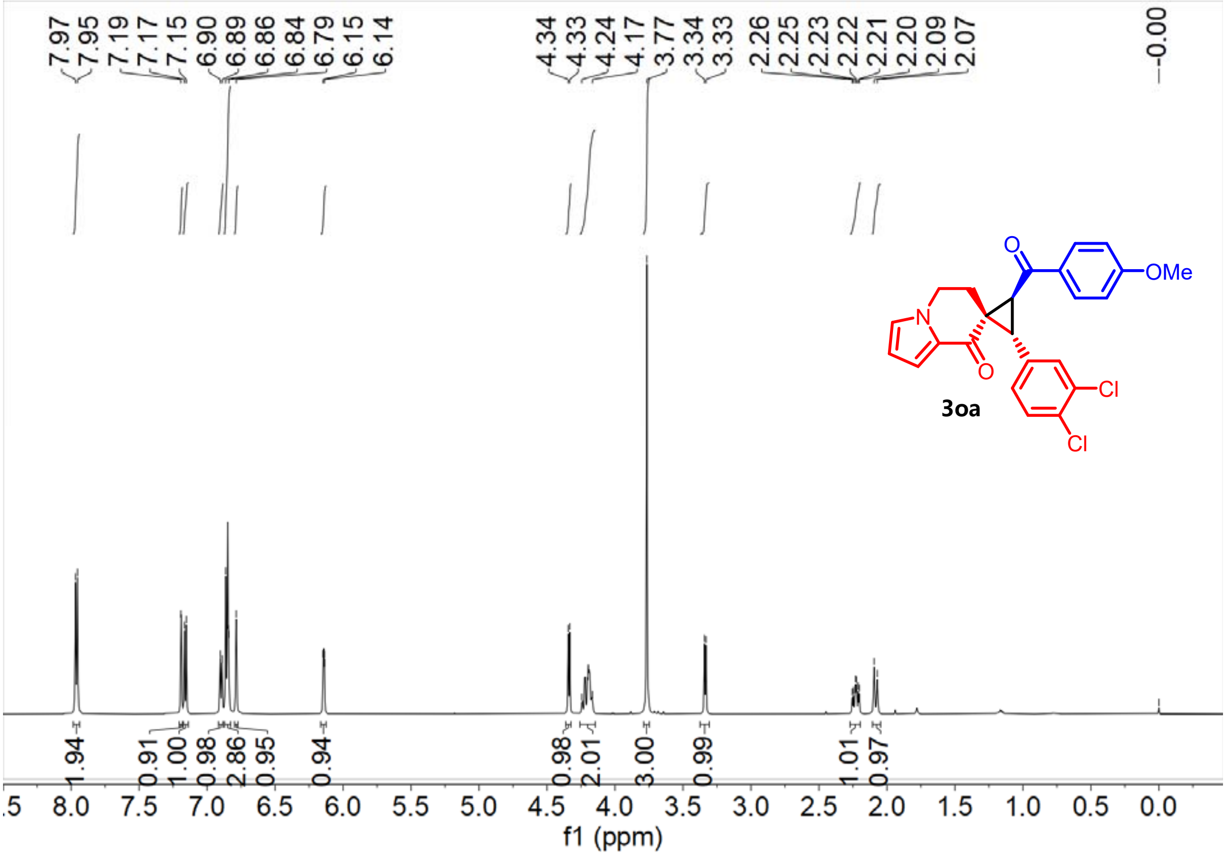


**Figure S31.** $^{1}$H-NMR of compound **3oa**

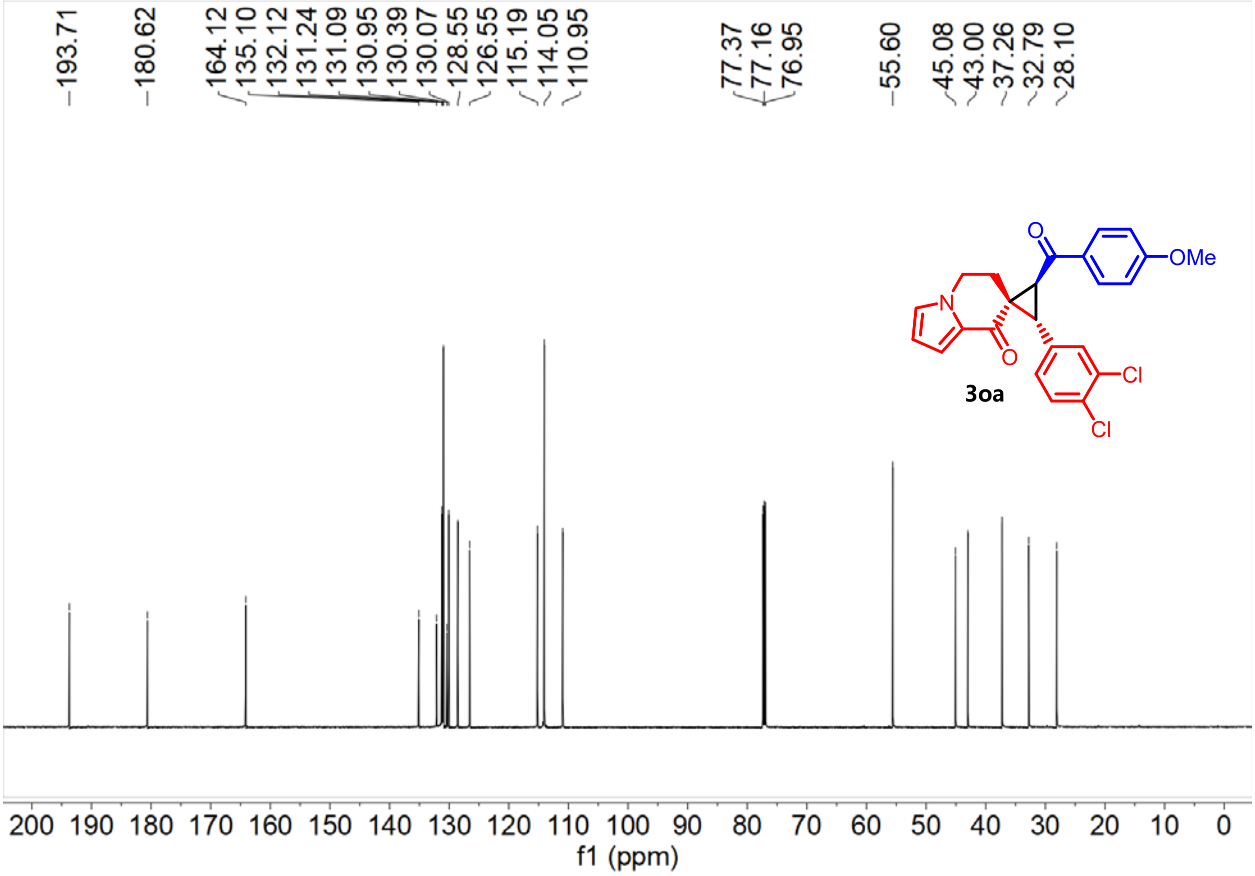


**Figure S32.** $^{13}$C-NMR of compound **3oa**

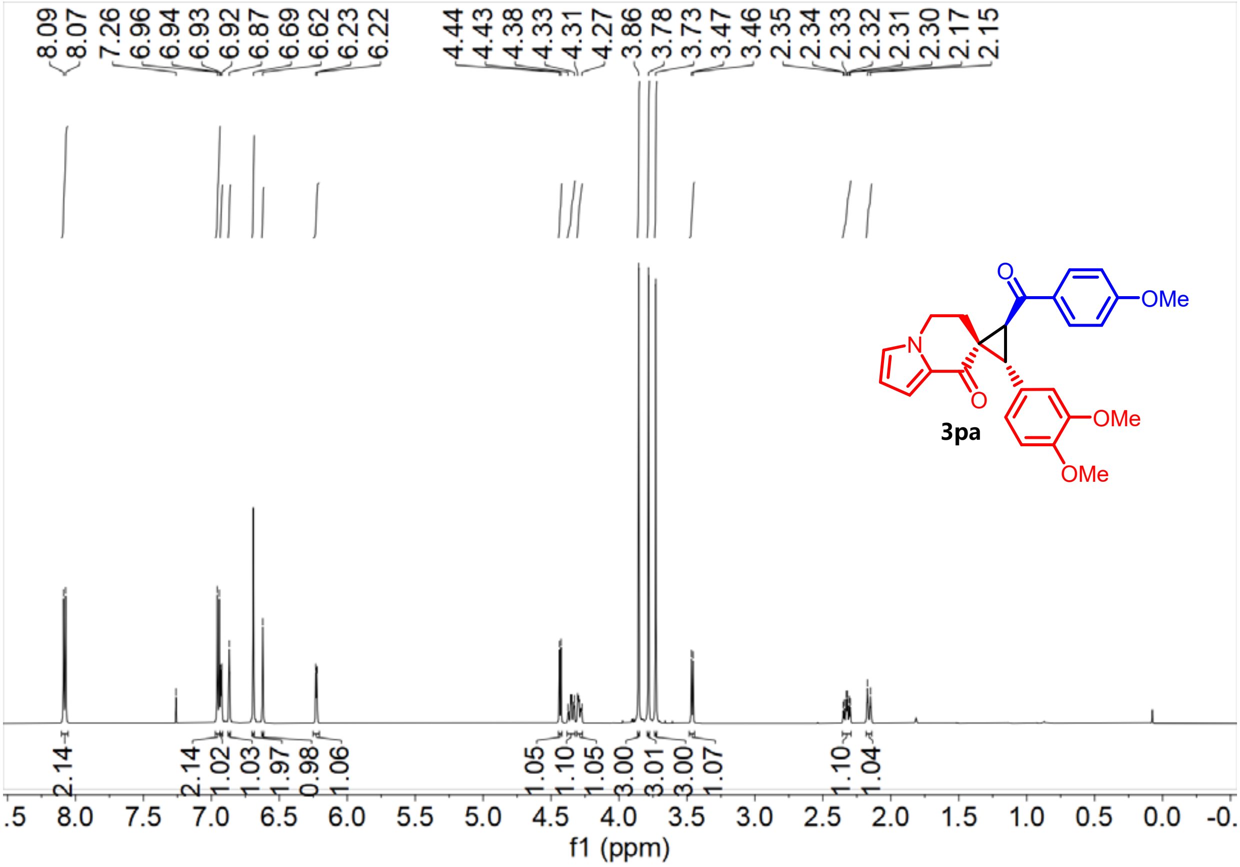


**Figure S33.** $^{1}$H-NMR of compound **3pa**

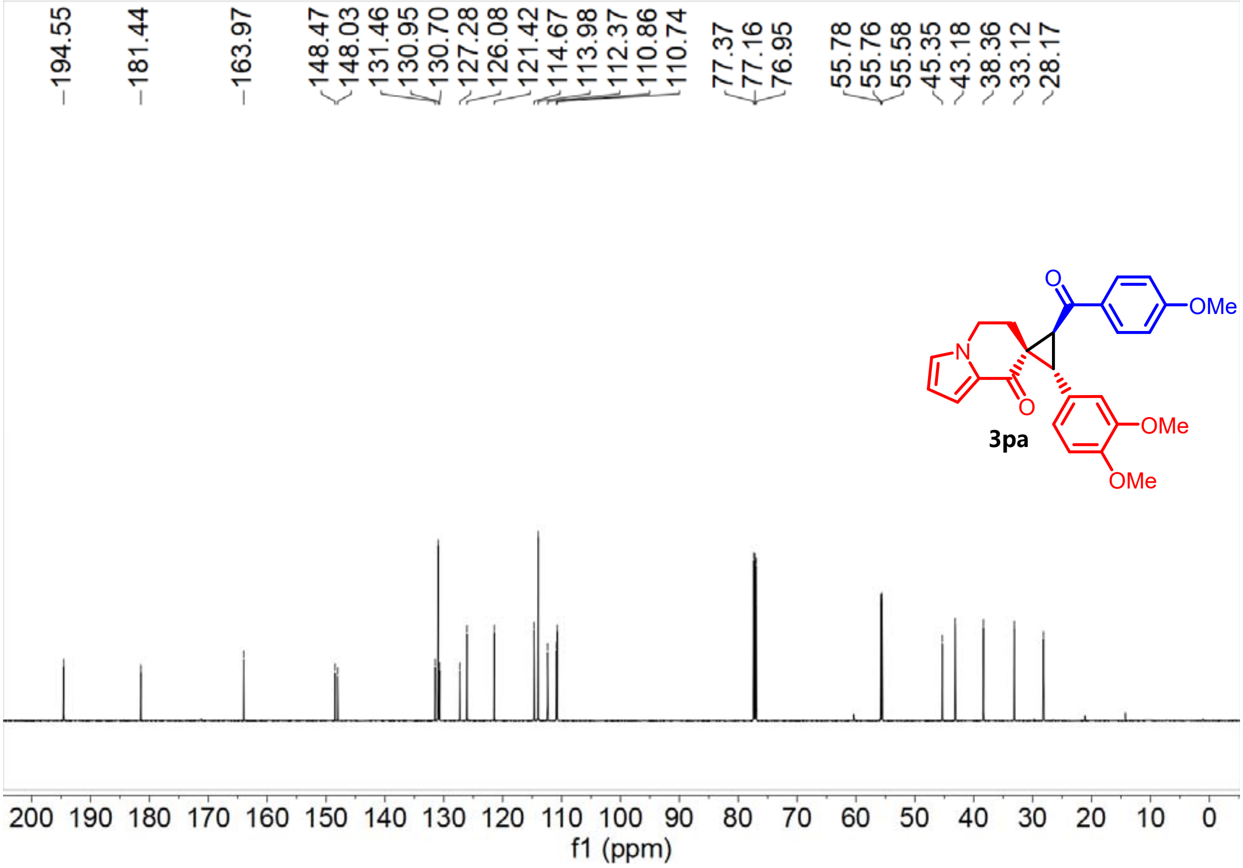


**Figure S34.** $^{13}$C-NMR of compound **3pa**

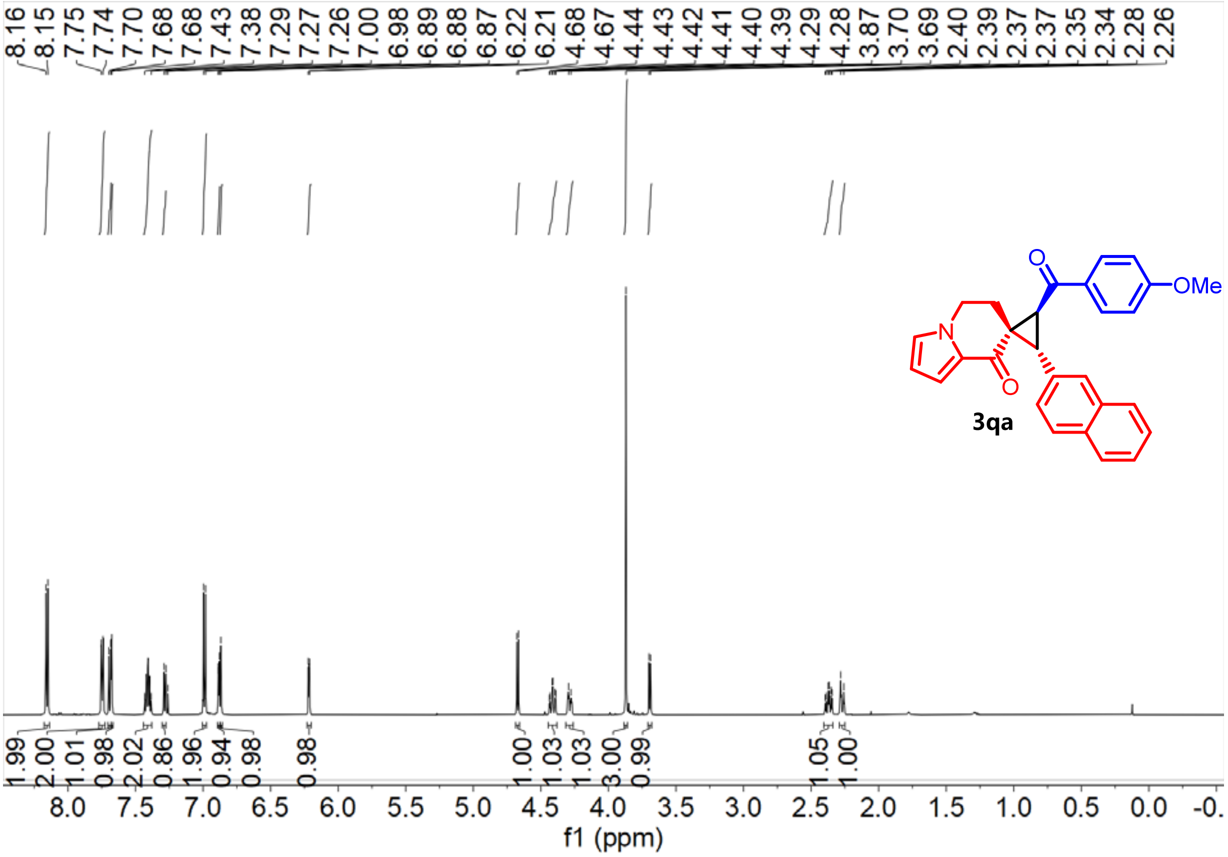

**Figure S35.** $^{1}$H-NMR of compound **3qa**

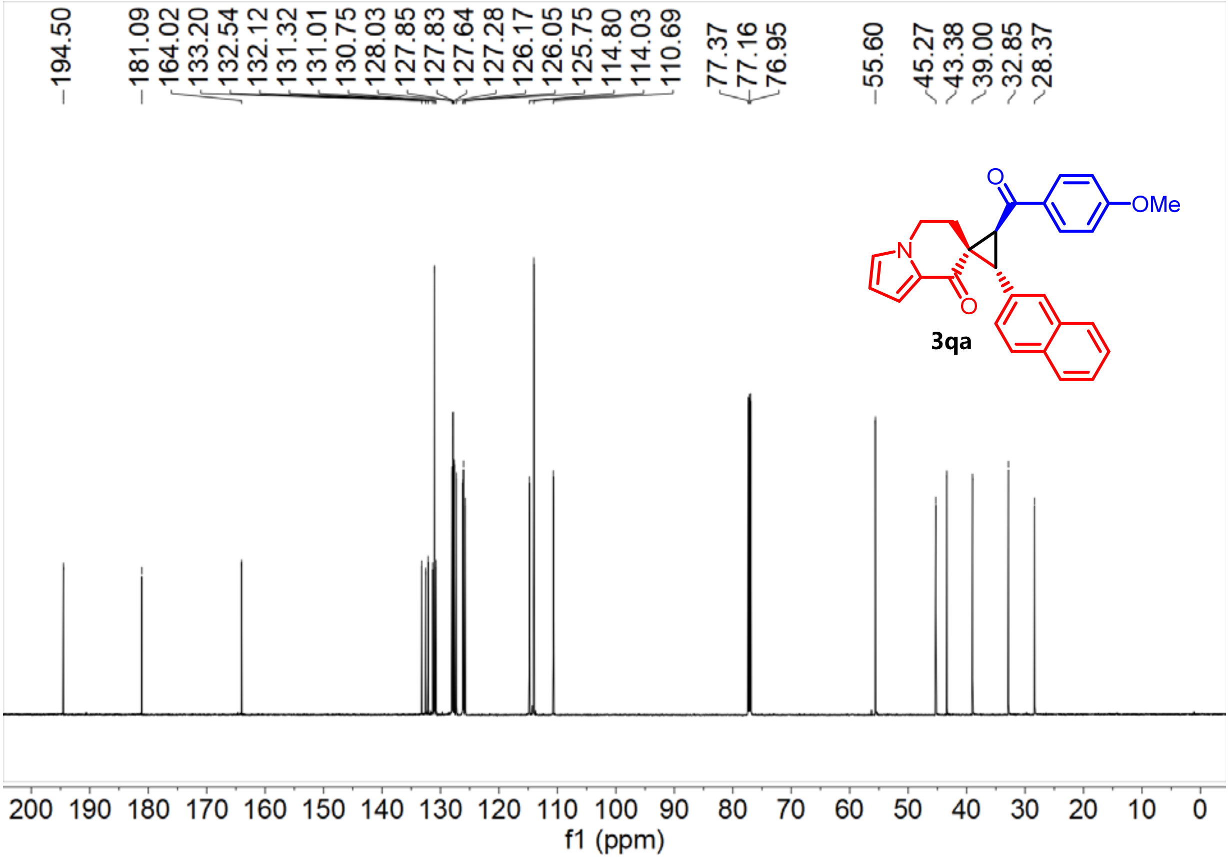

**Figure S36.** $^{13}$C-NMR of compound **3qa**

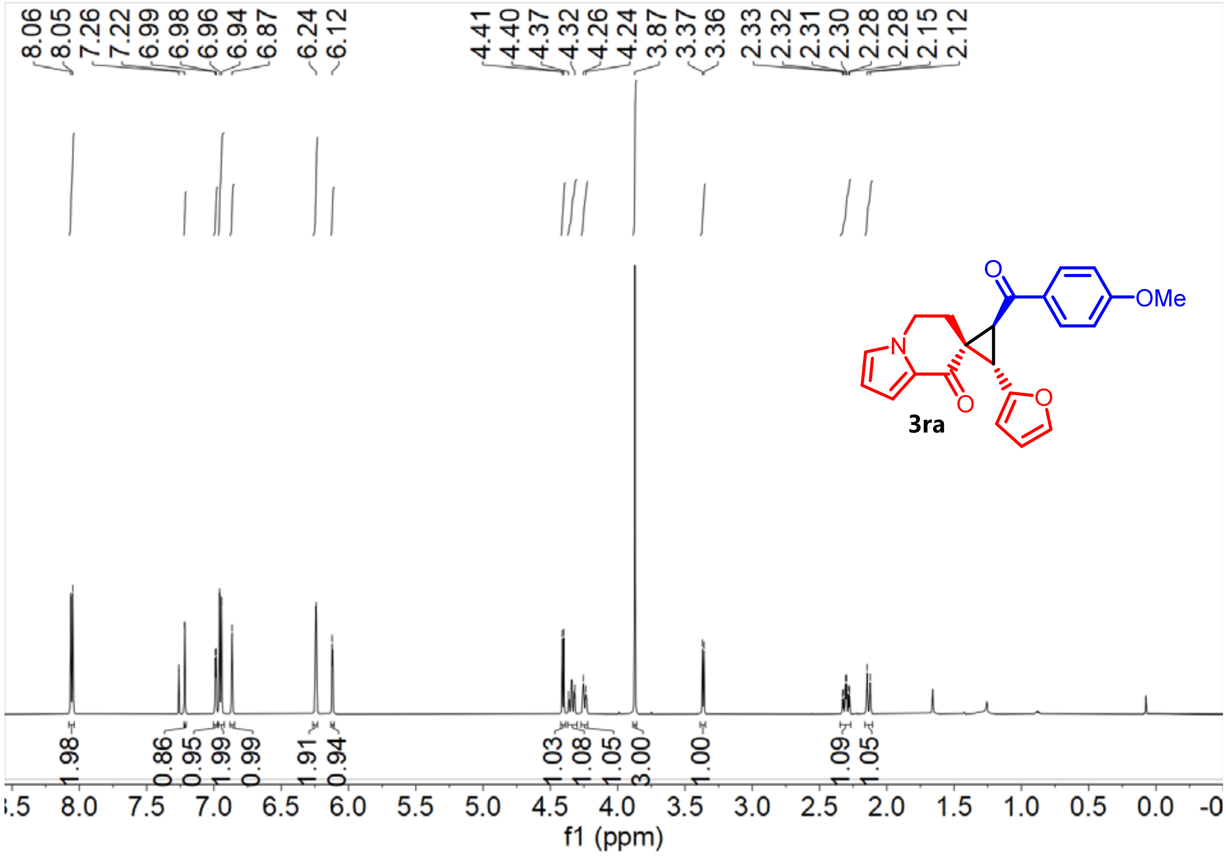

**Figure S37.** $^{1}$H-NMR of compound **3ra**

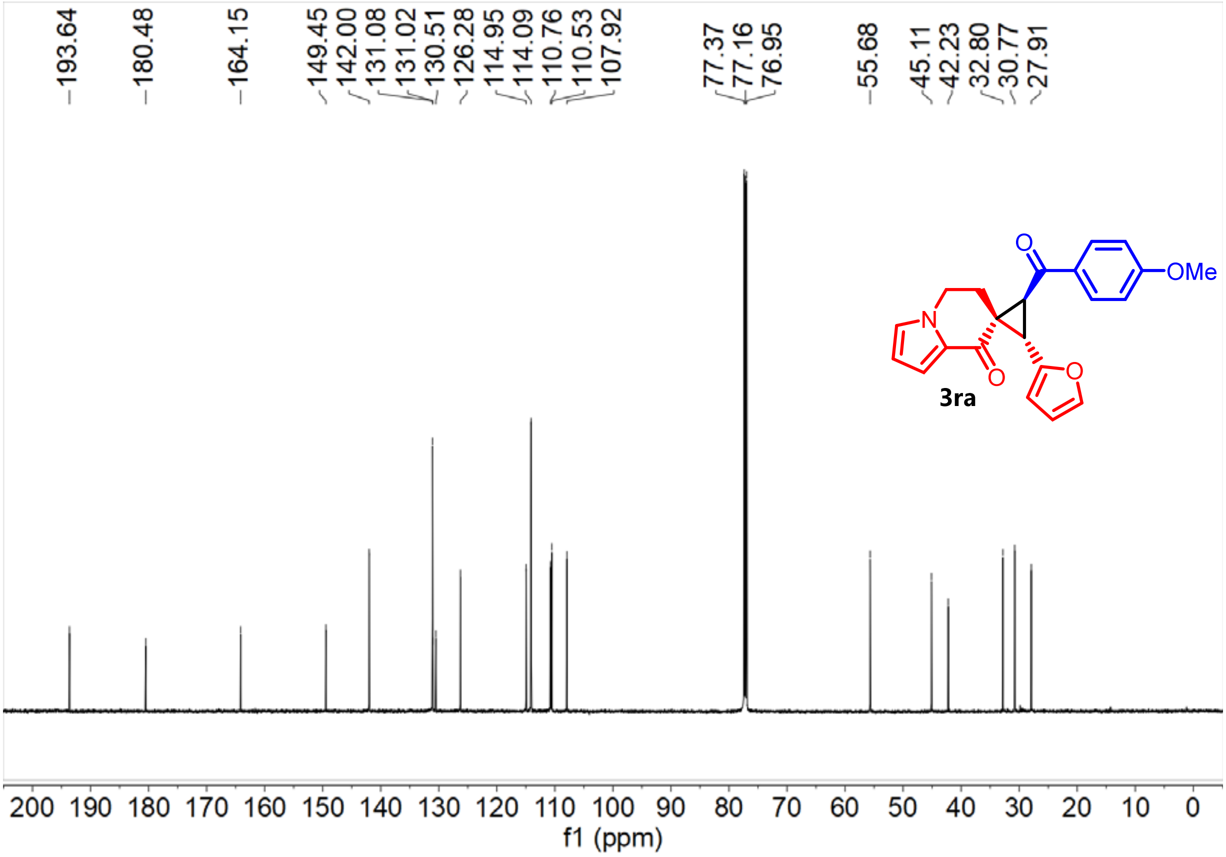

**Figure S38.** $^{13}$C-NMR of compound **3ra**

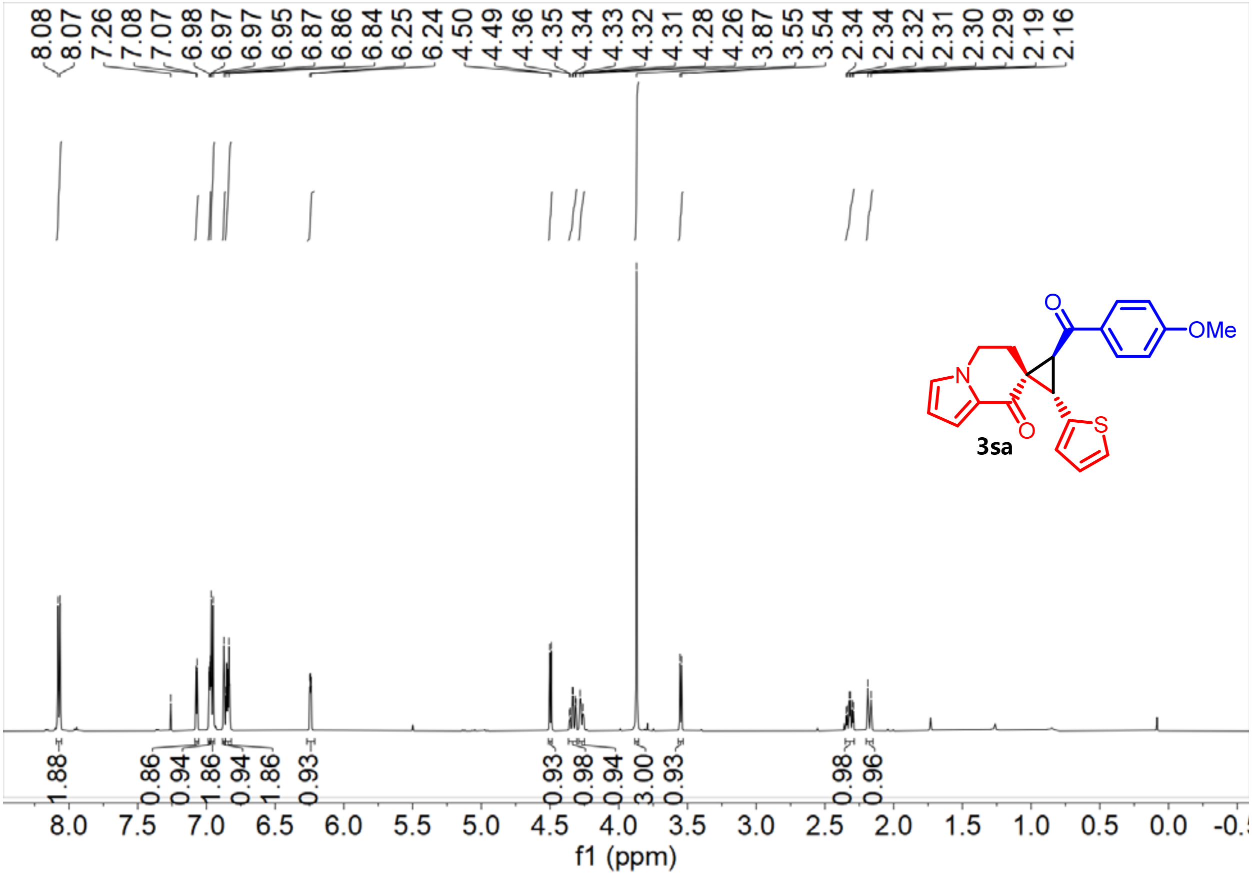


**Figure S39.** $^{1}$H-NMR of compound **3sa**

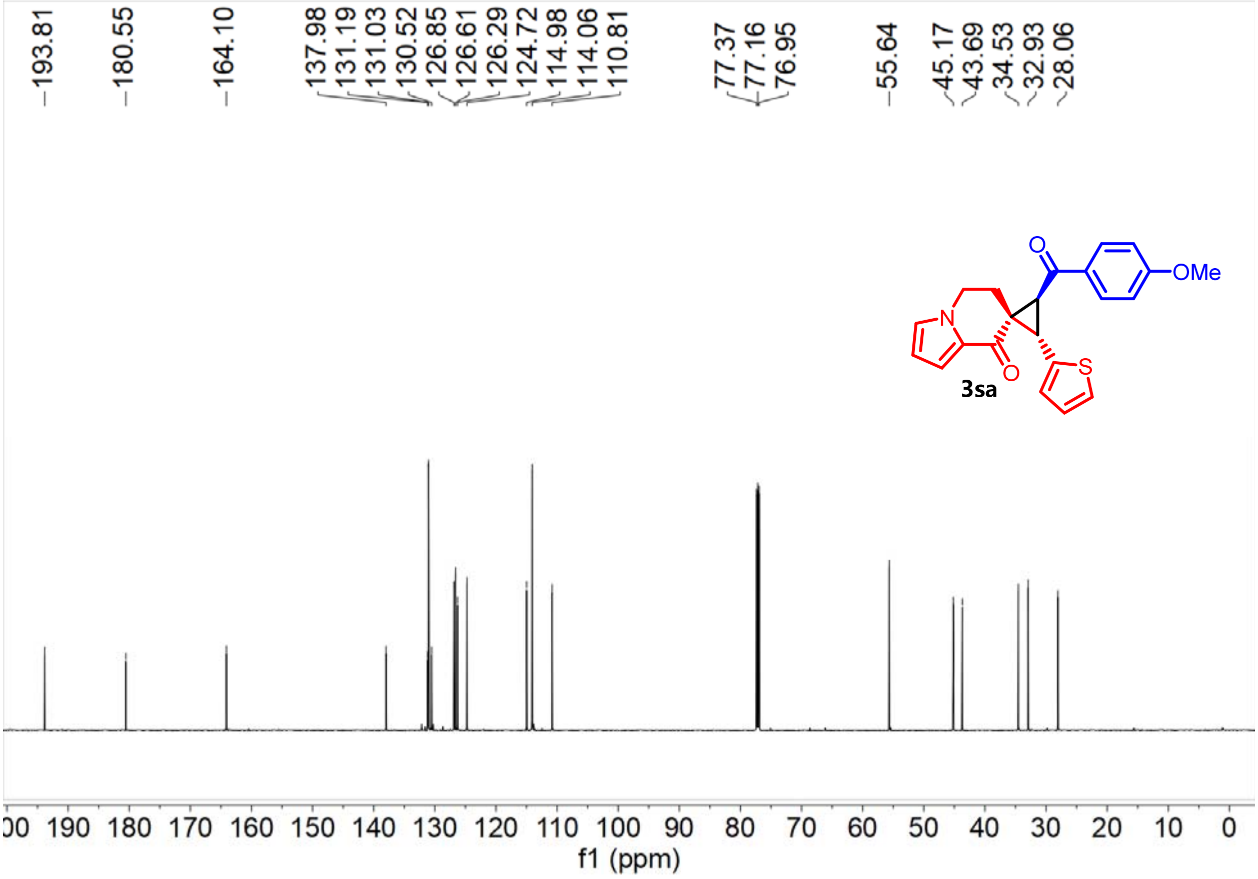


**Figure S40.** $^{13}$C-NMR of compound **3sa**

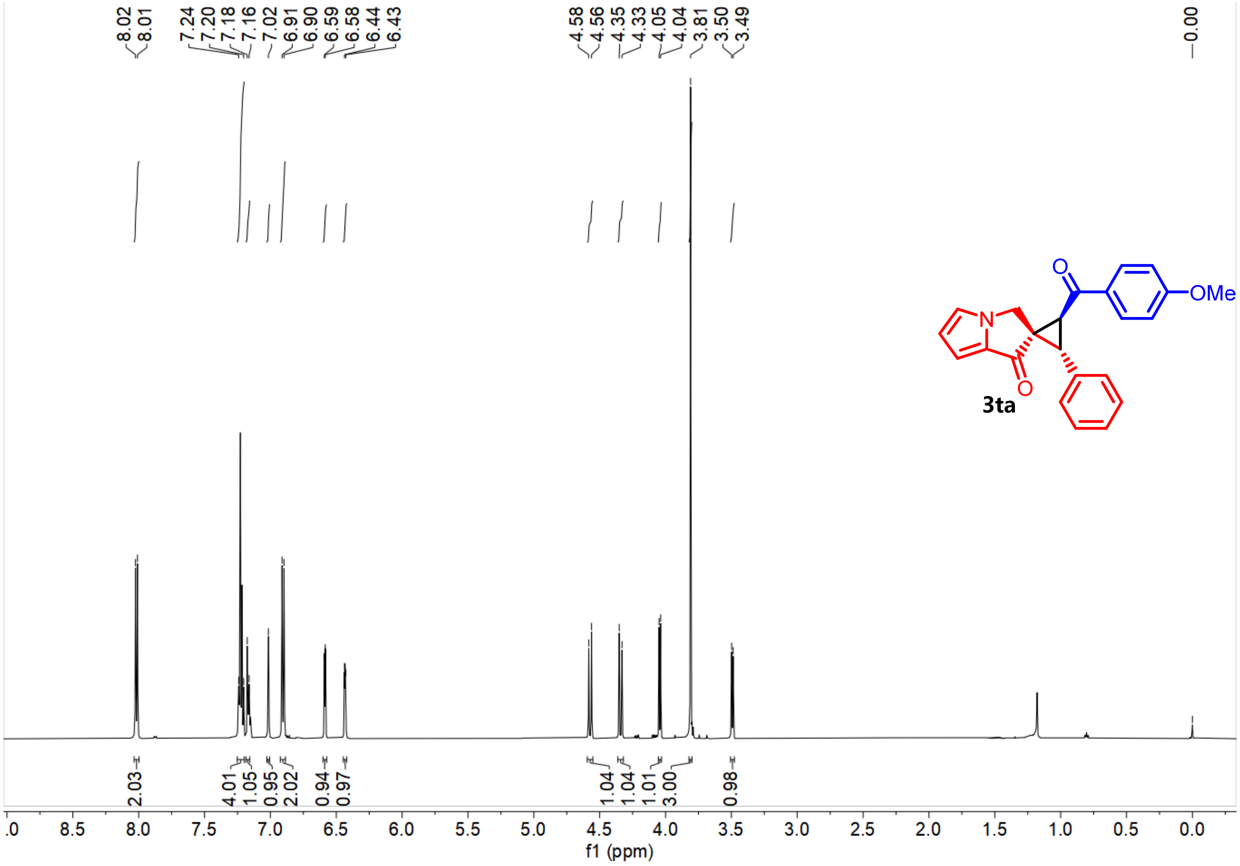


**Figure S41.** $^{1}H$-NMR of compound **3ta**

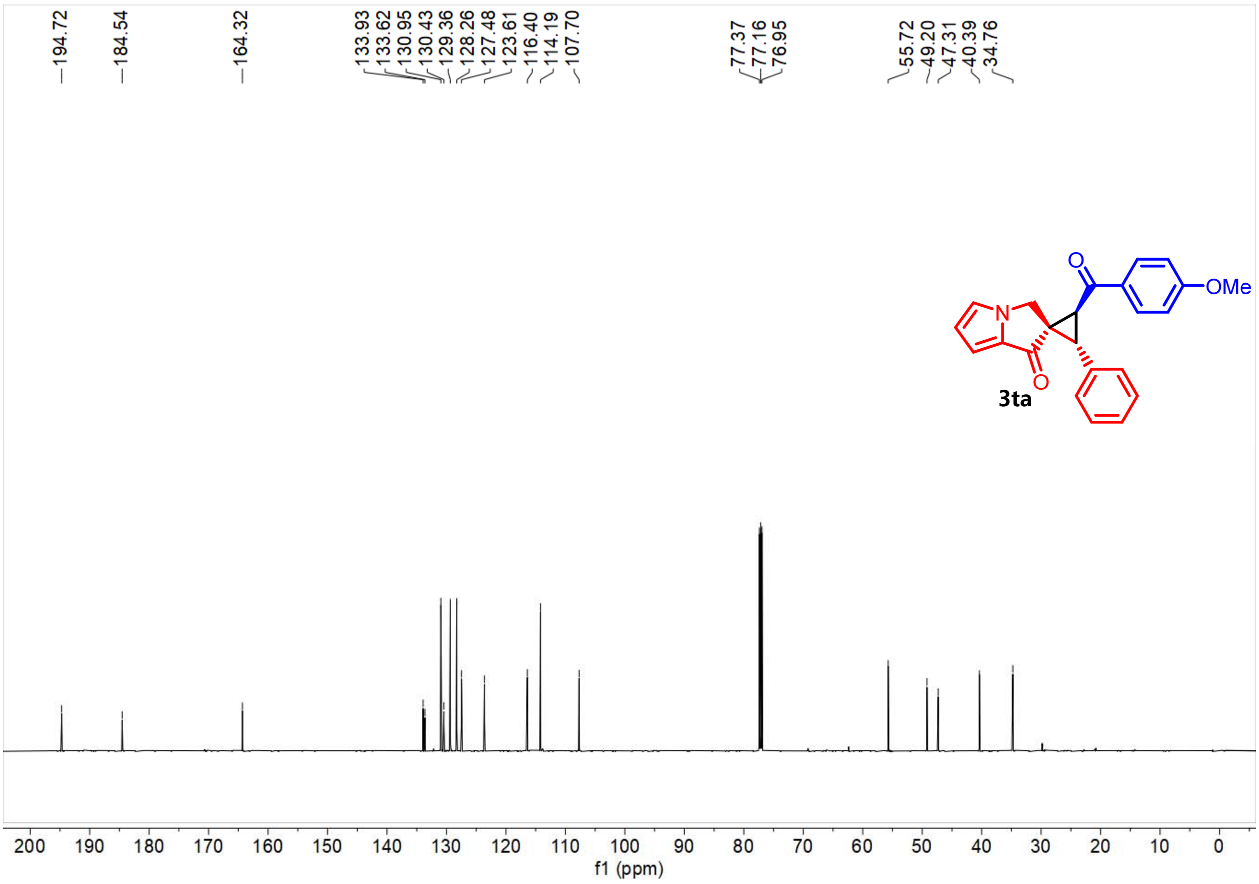


**Figure S42.** $^{13}C$-NMR of compound **3ta**

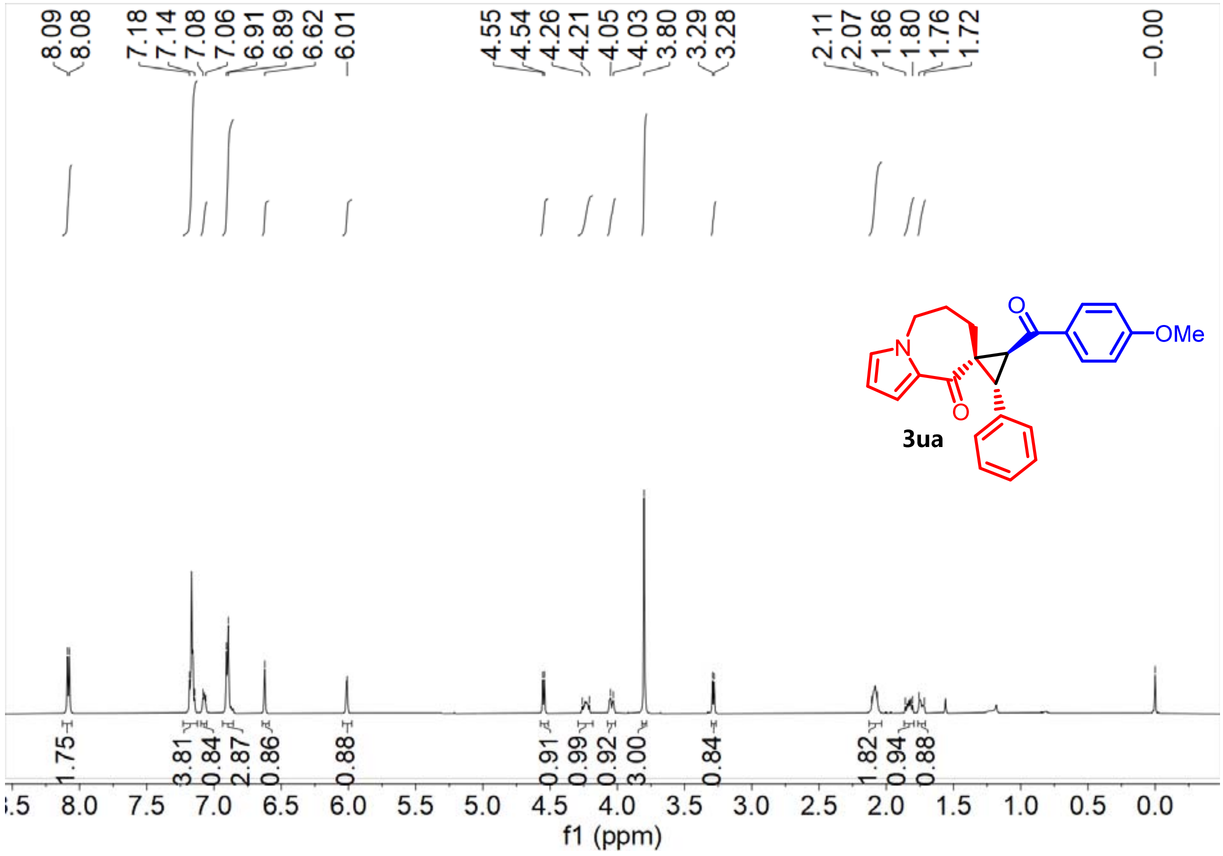

**Figure S43.** $^{1}$H-NMR of compound **3ua**

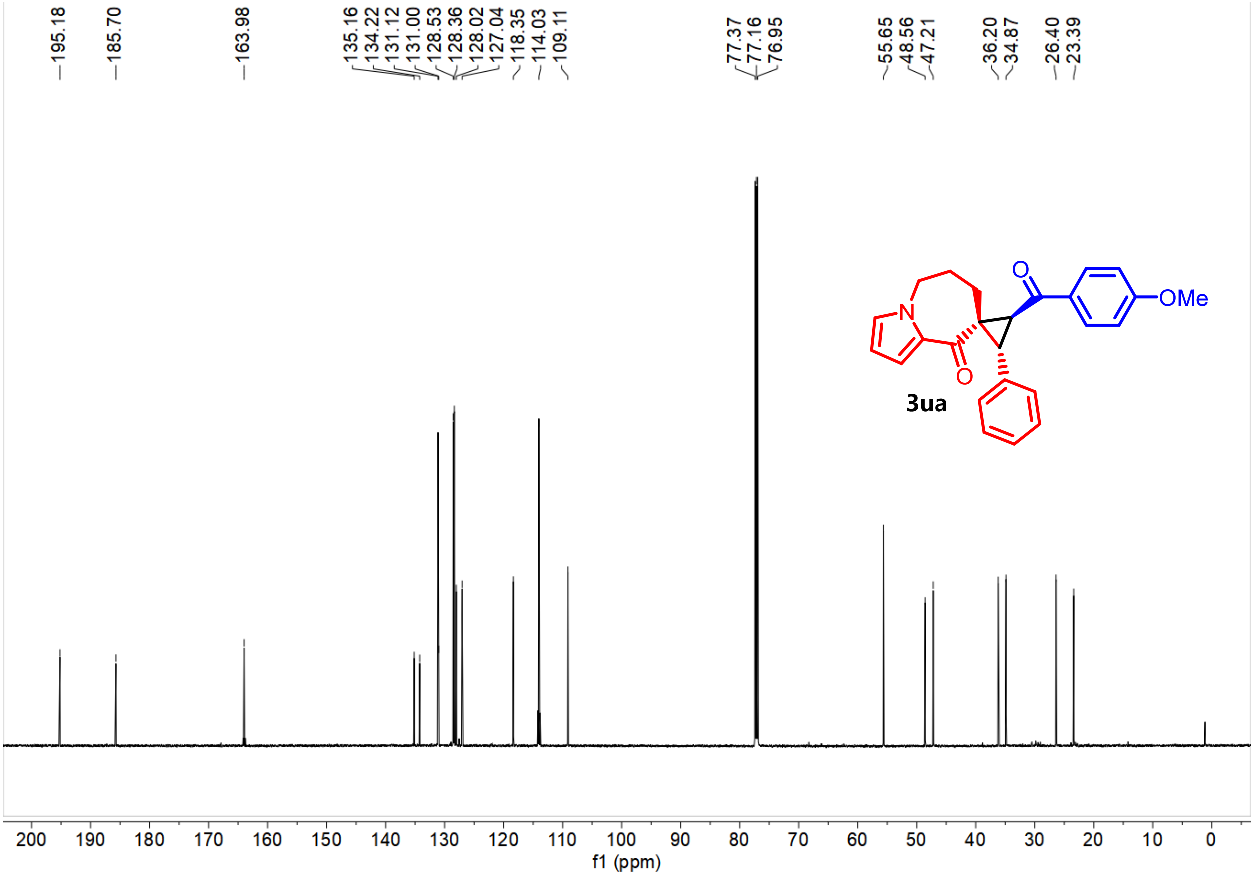

**Figure S44.** $^{13}$C-NMR of compound **3ua**

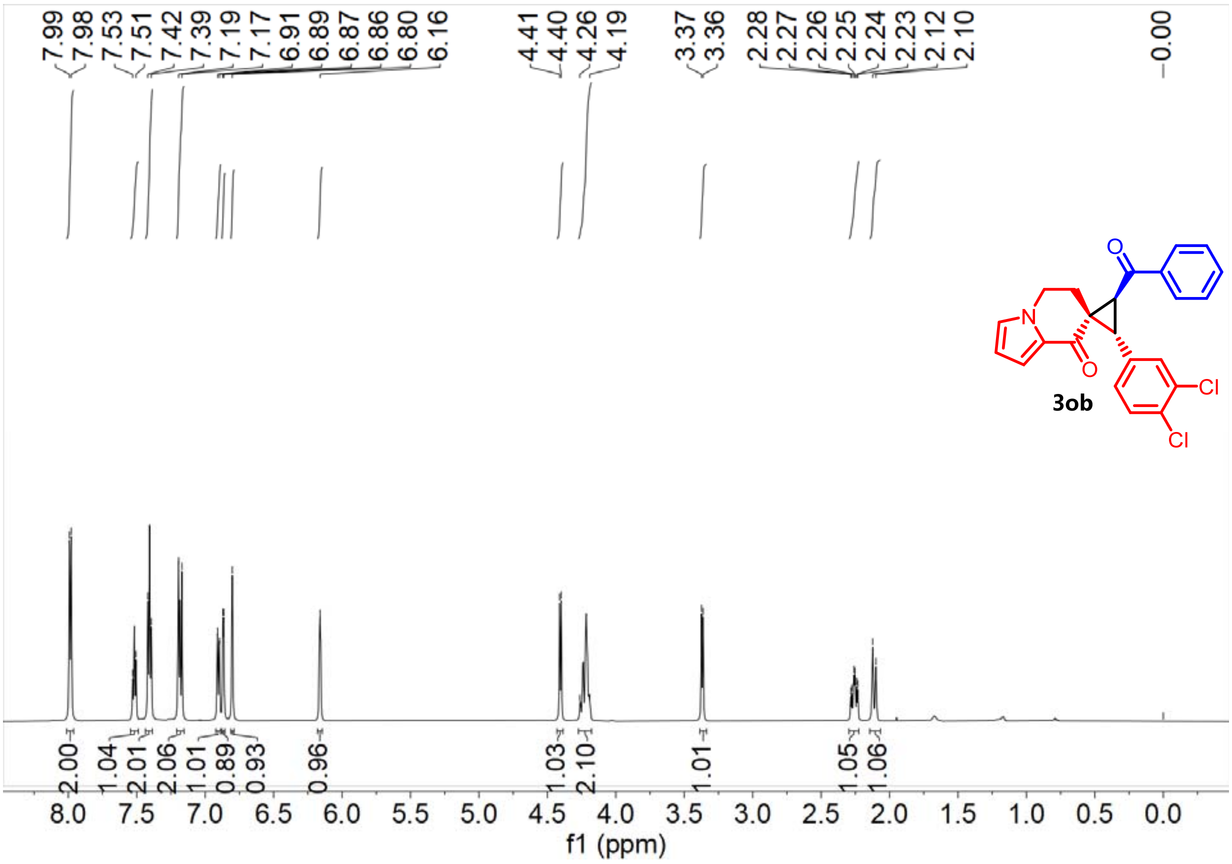


**Figure S45.** $^{1}$H-NMR of compound **3ob**

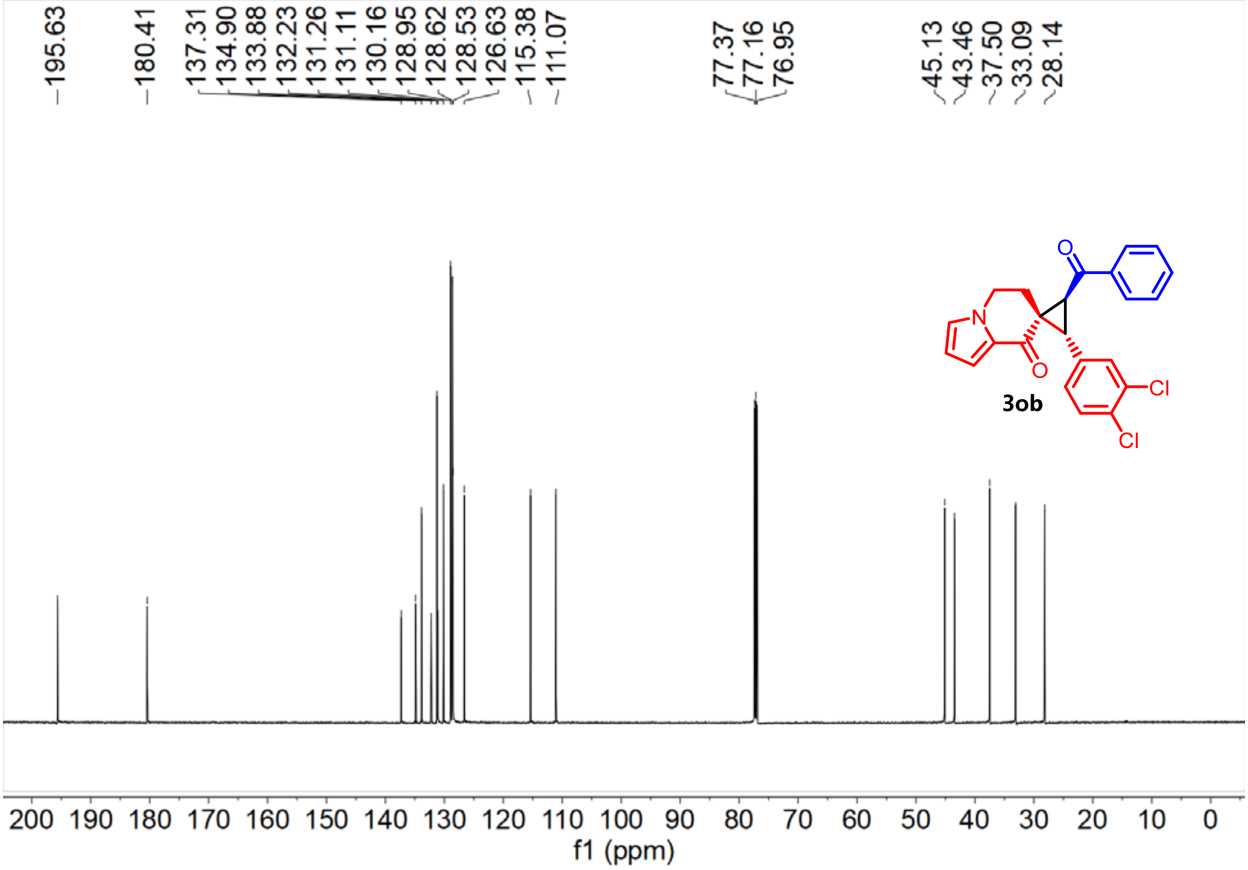


**Figure S46.** $^{13}$C-NMR of compound **3ob**

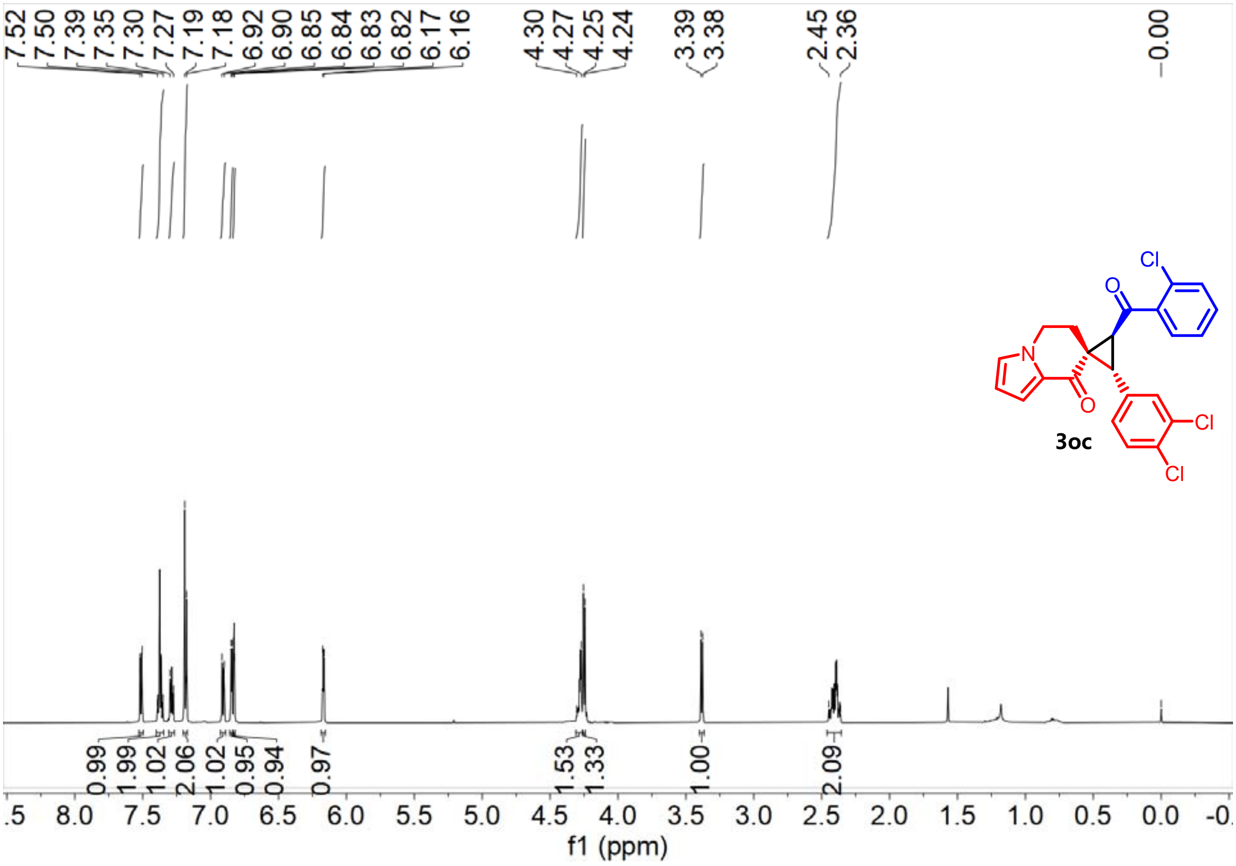


**Figure S47.** $^{1}$H-NMR of compound **3oc**

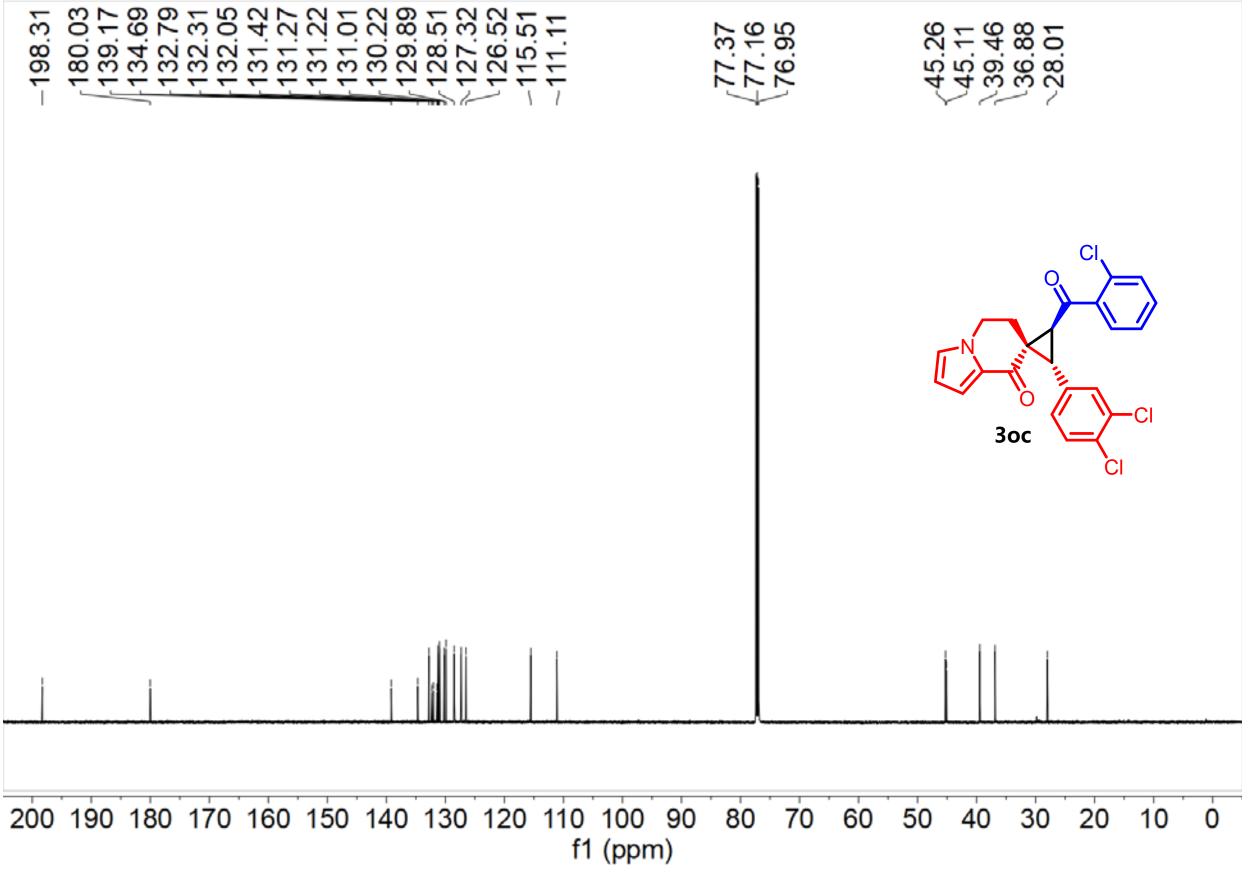


**Figure S48.** $^{13}$C-NMR of compound **3oc**

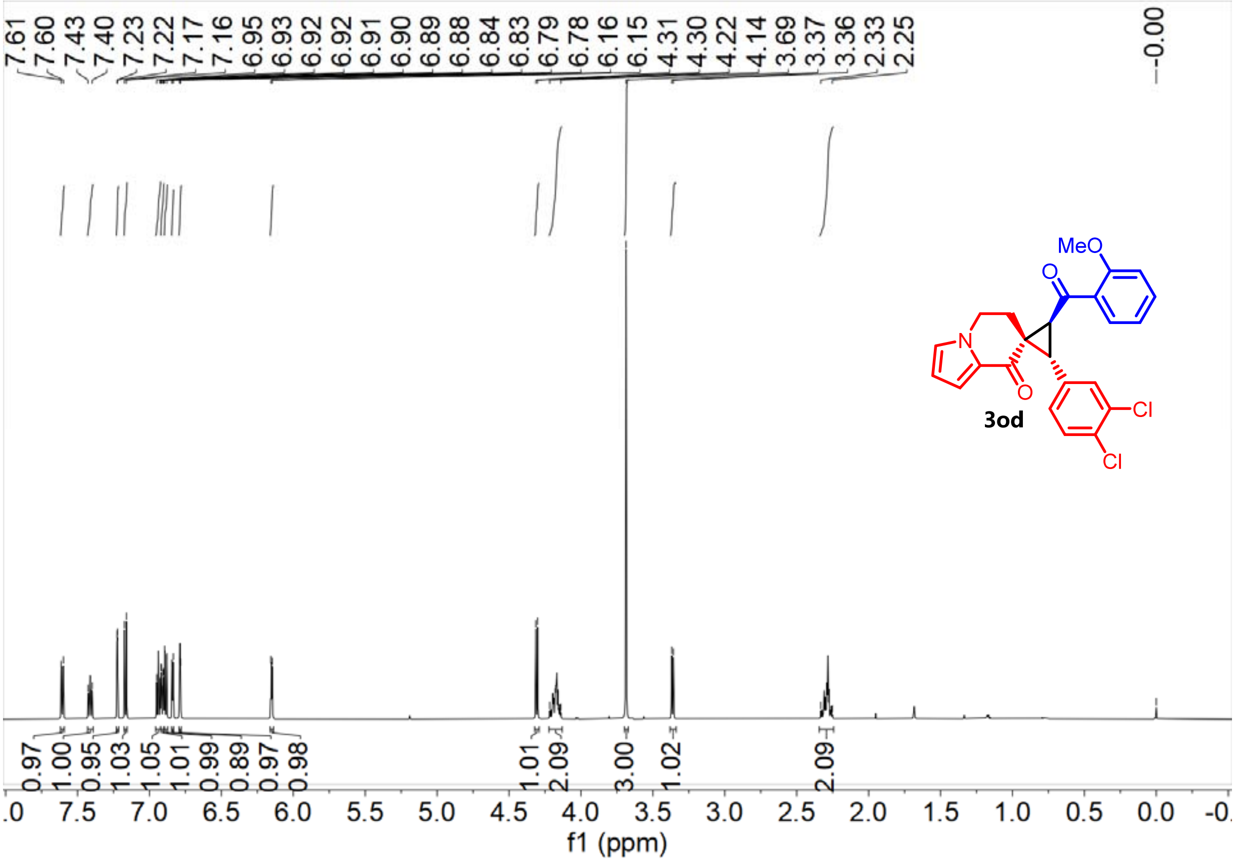


**Figure S49.** $^{1}$H-NMR of compound **3od**

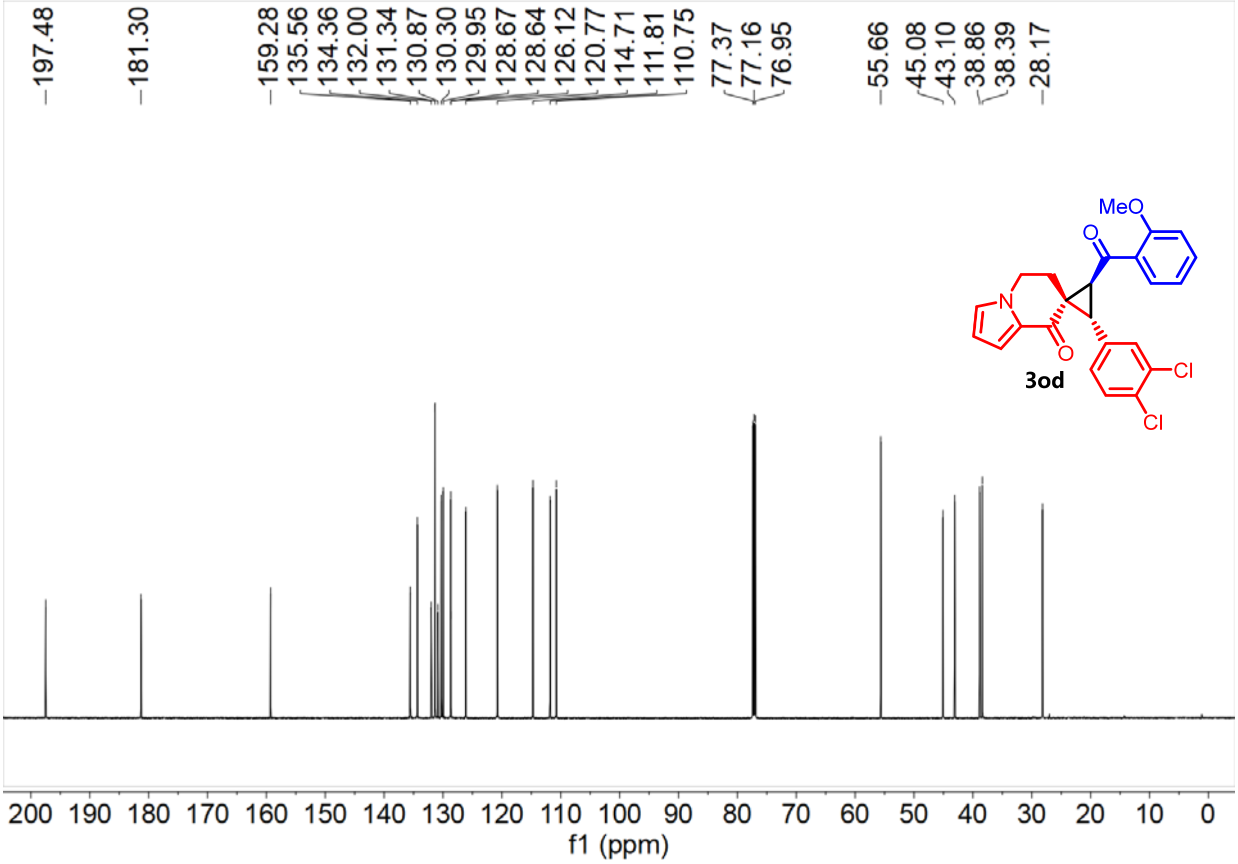


**Figure S50.** $^{13}$C-NMR of compound **3od**

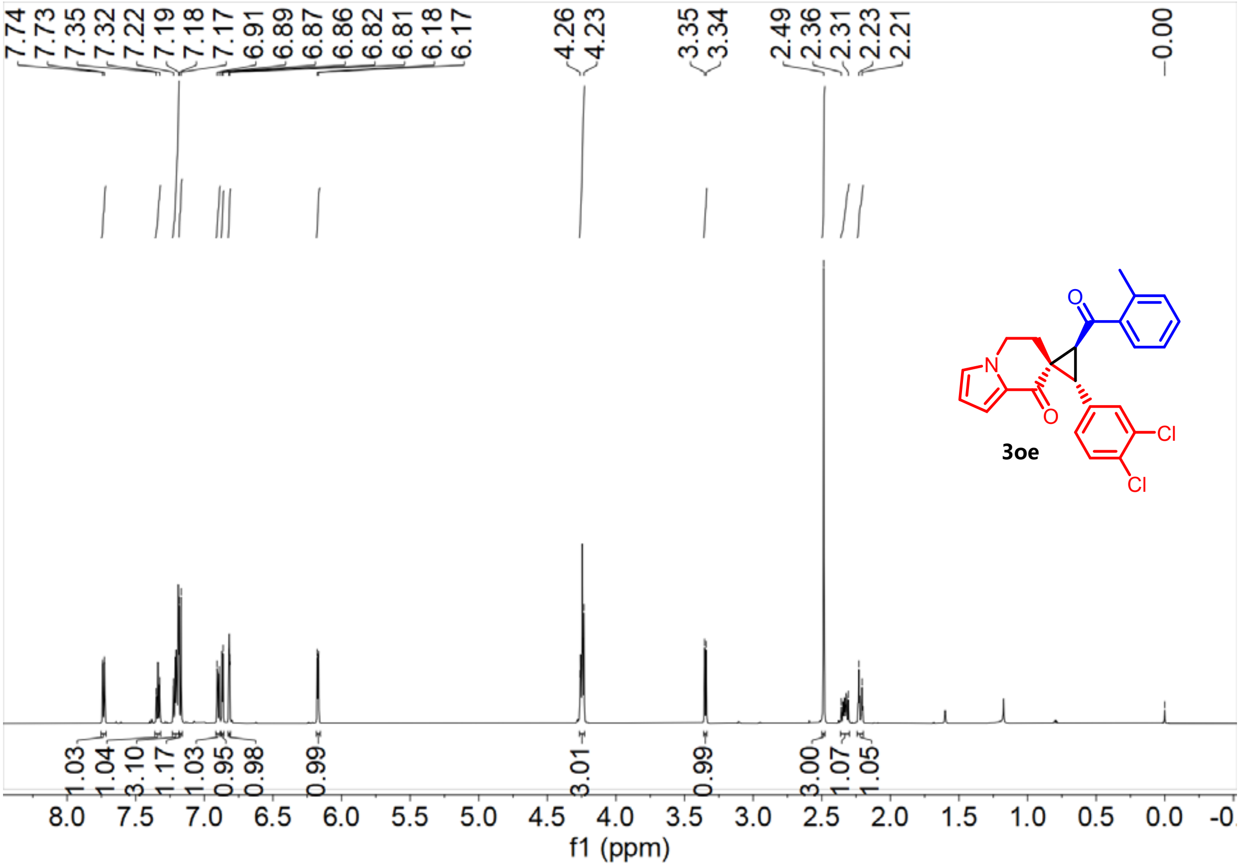


**Figure S51.** $^{1}$H-NMR of compound **3oe**

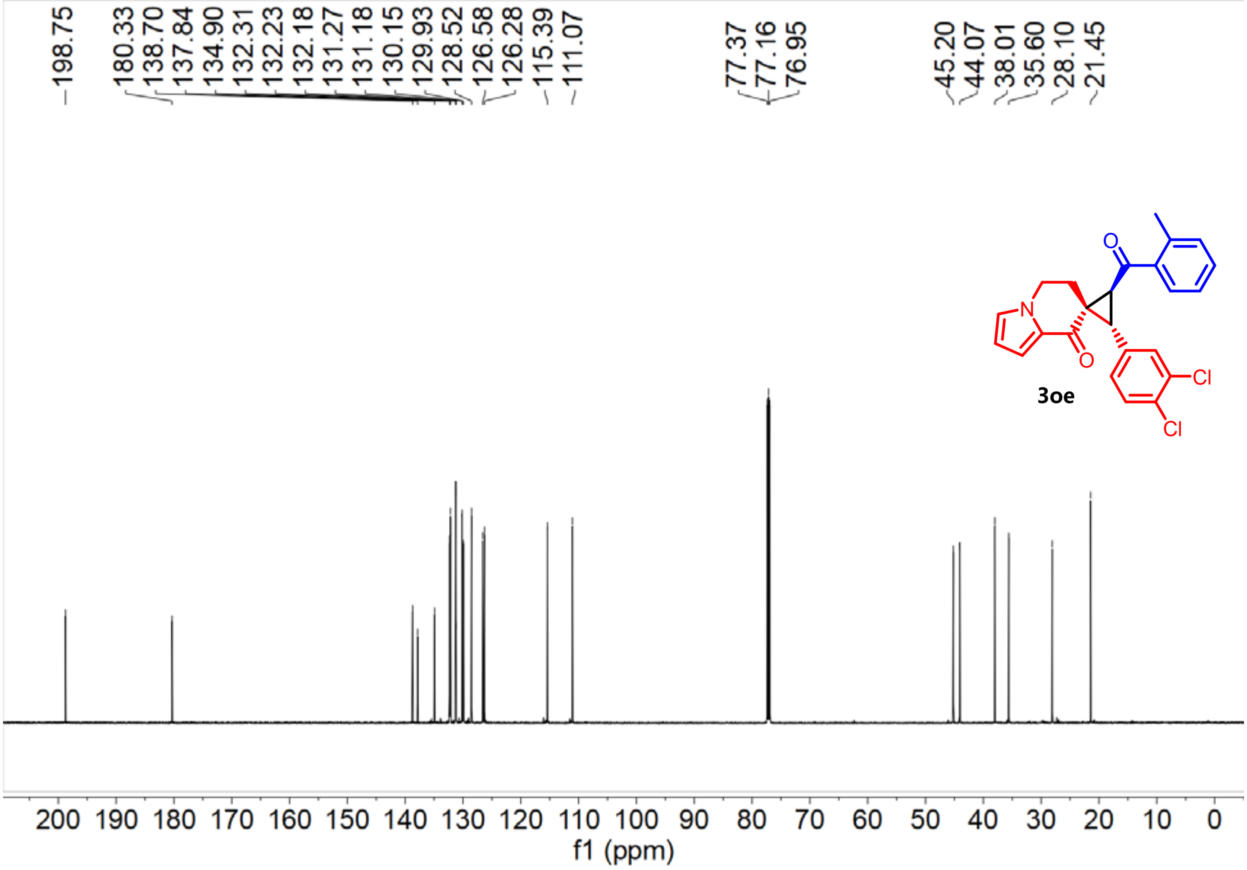


**Figure S52.** $^{13}$C-NMR of compound **3oe**

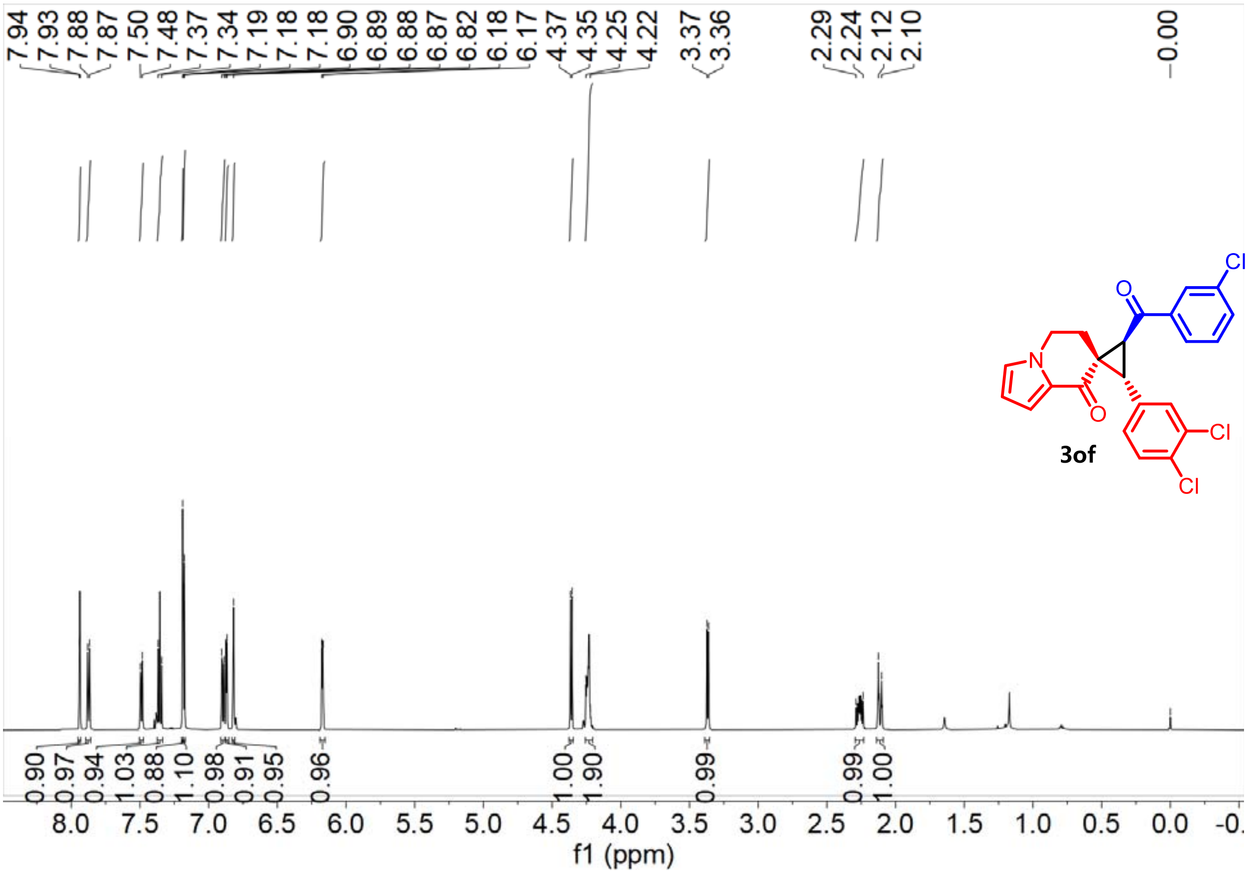


**Figure S53.** $^{1}$H-NMR of compound **3of**

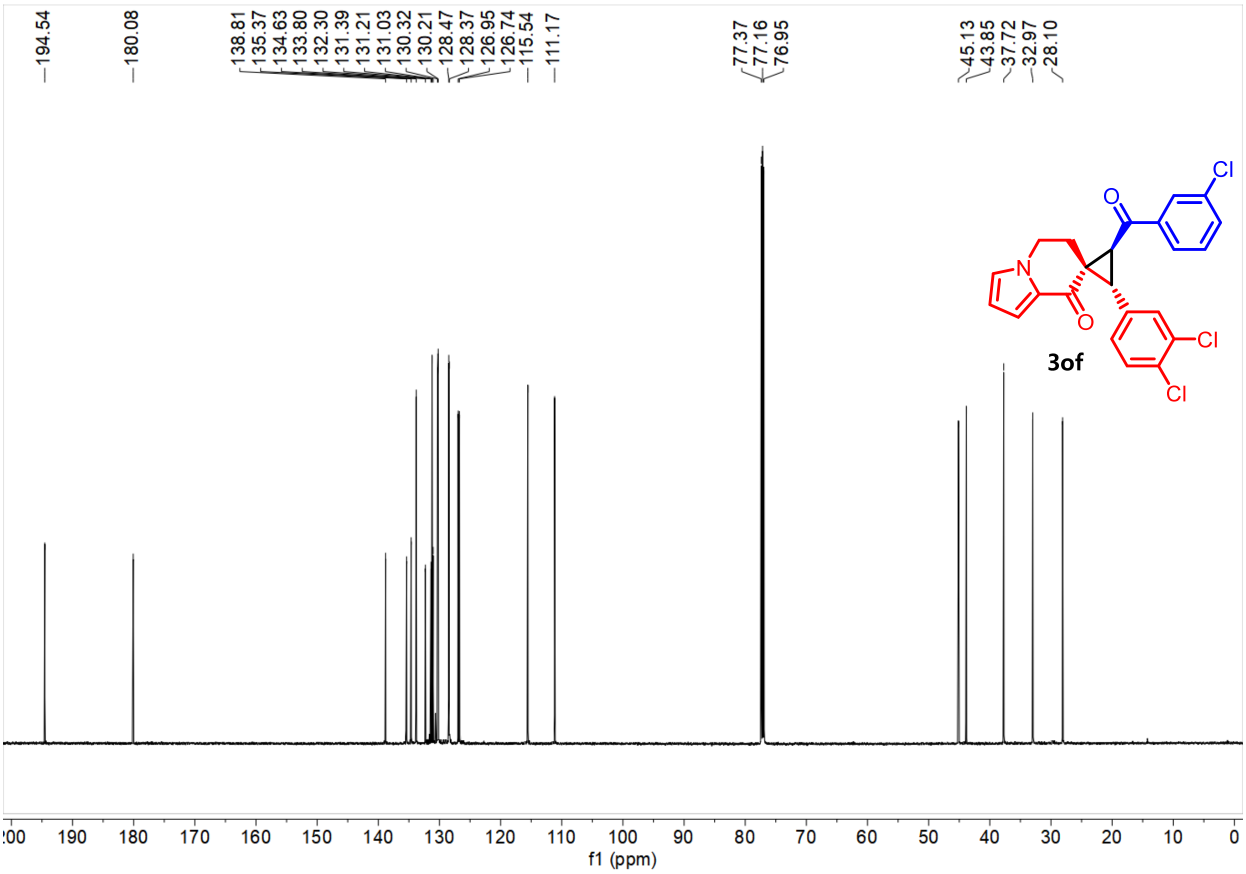


**Figure S54.** $^{13}$C-NMR of compound **3of**

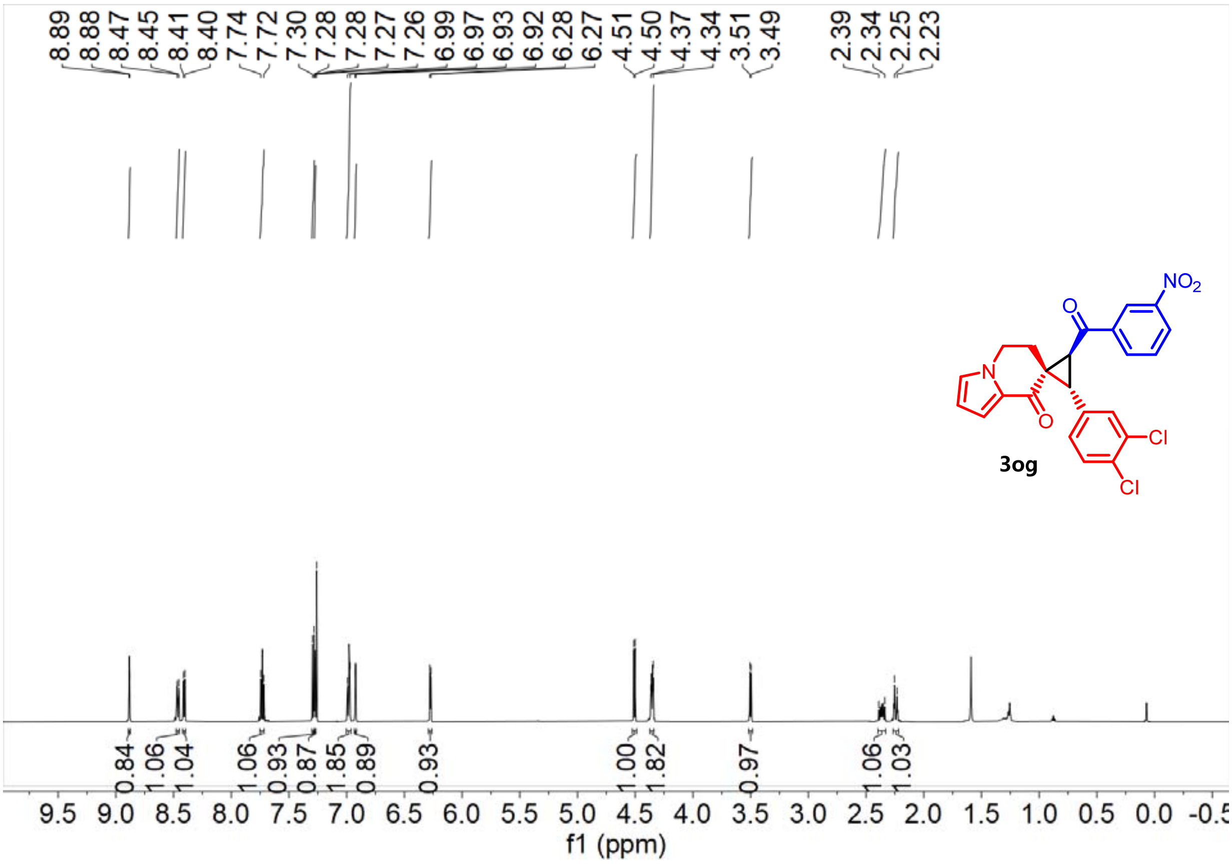

**Figure S55.** $^{1}$H-NMR of compound **3og**

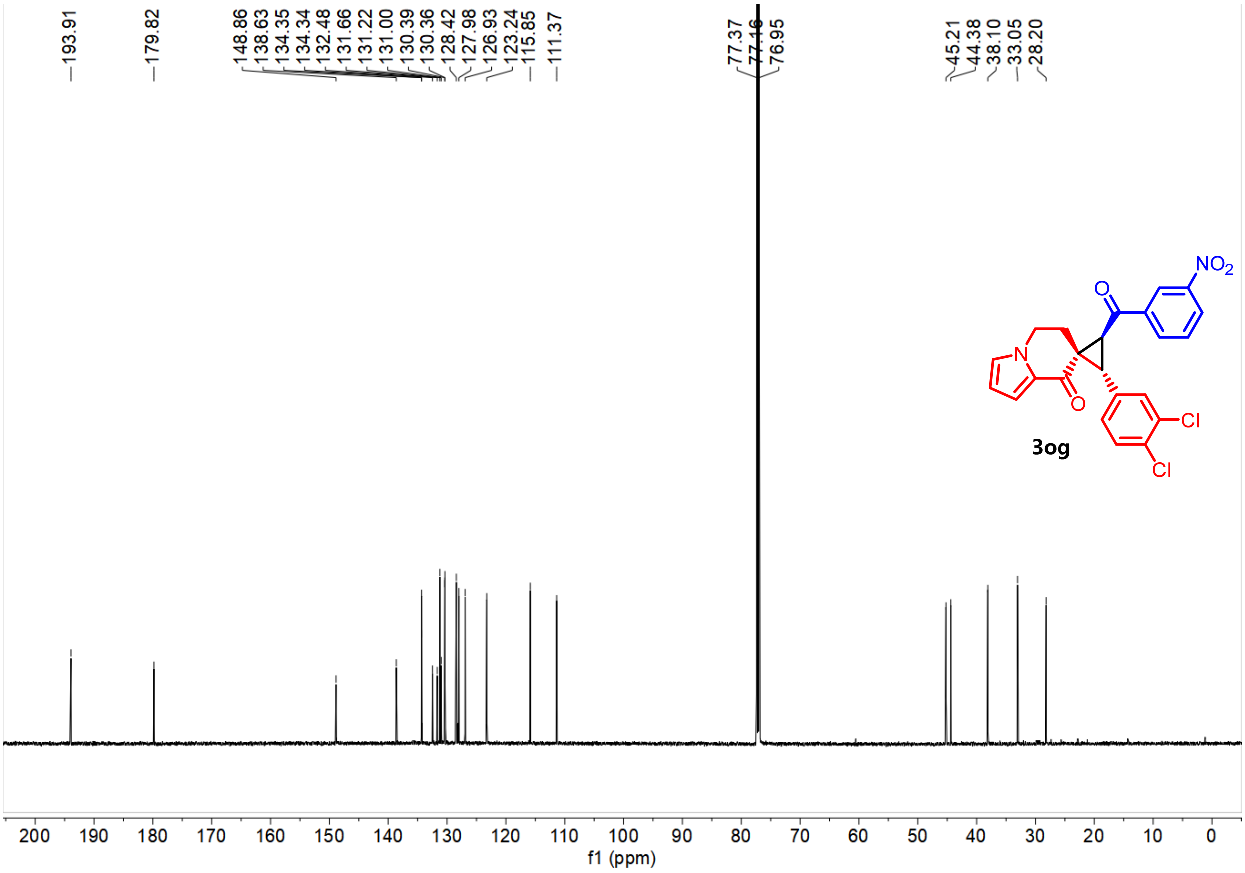

**Figure S56.** $^{13}$C-NMR of compound **3og**

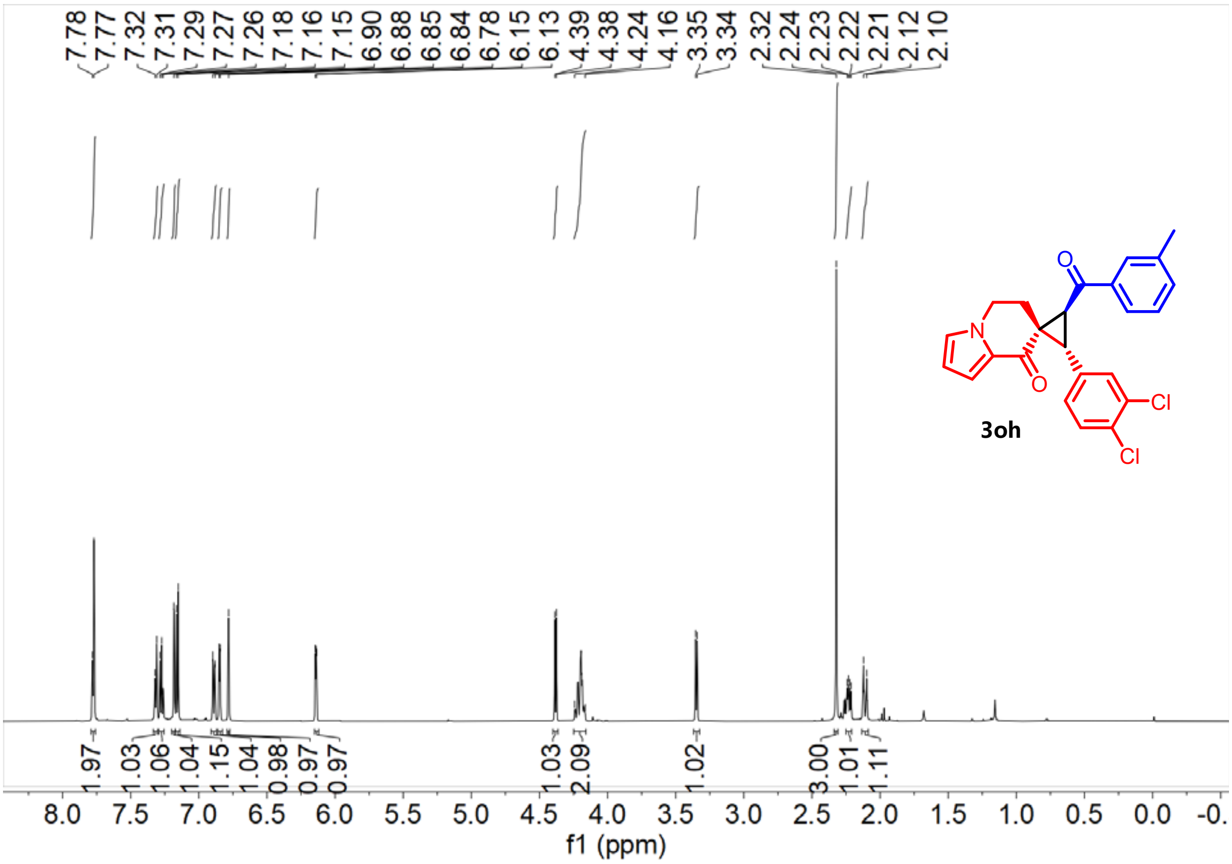


**Figure S57.** $^{1}H$-NMR of compound **3oh**

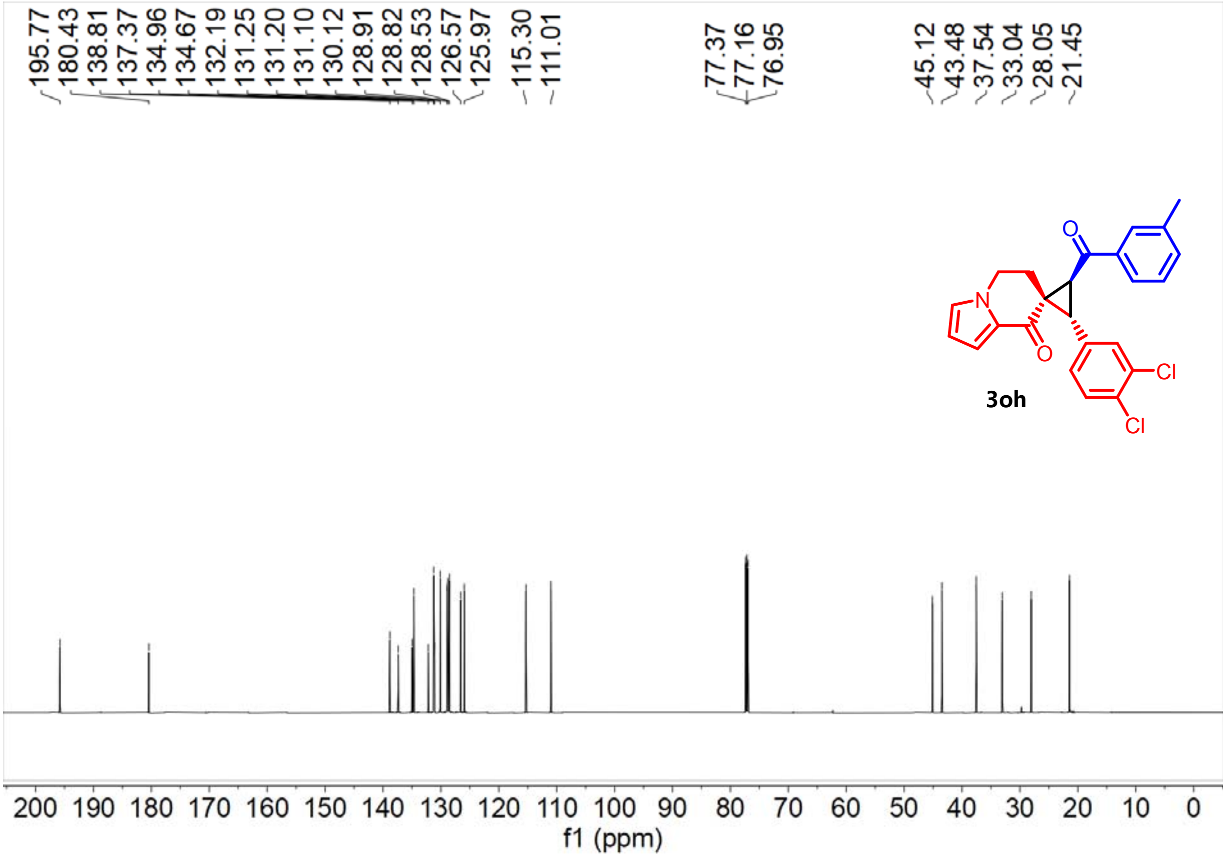


**Figure S58.** $^{13}C$-NMR of compound **3oh**

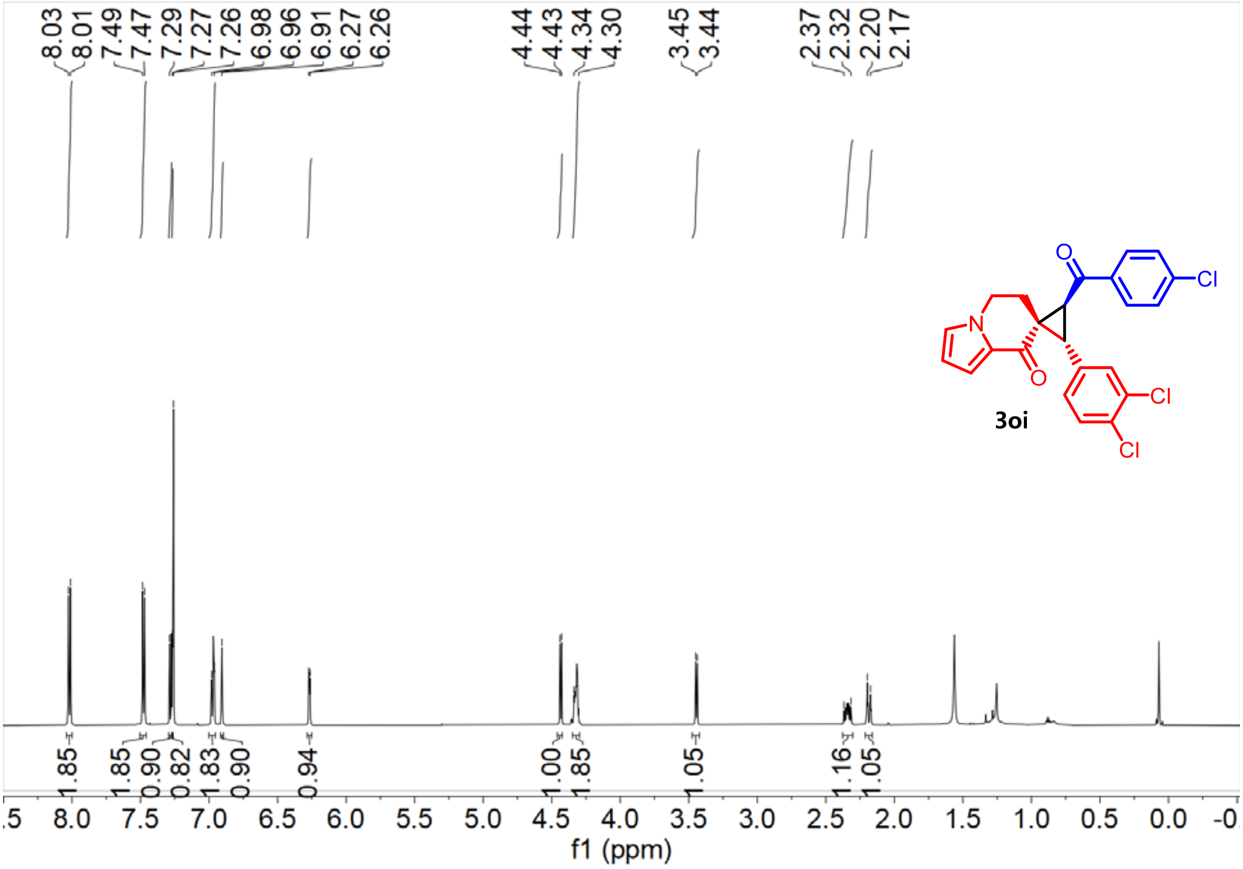


**Figure S59.** $^{1}$H-NMR of compound **3oi**

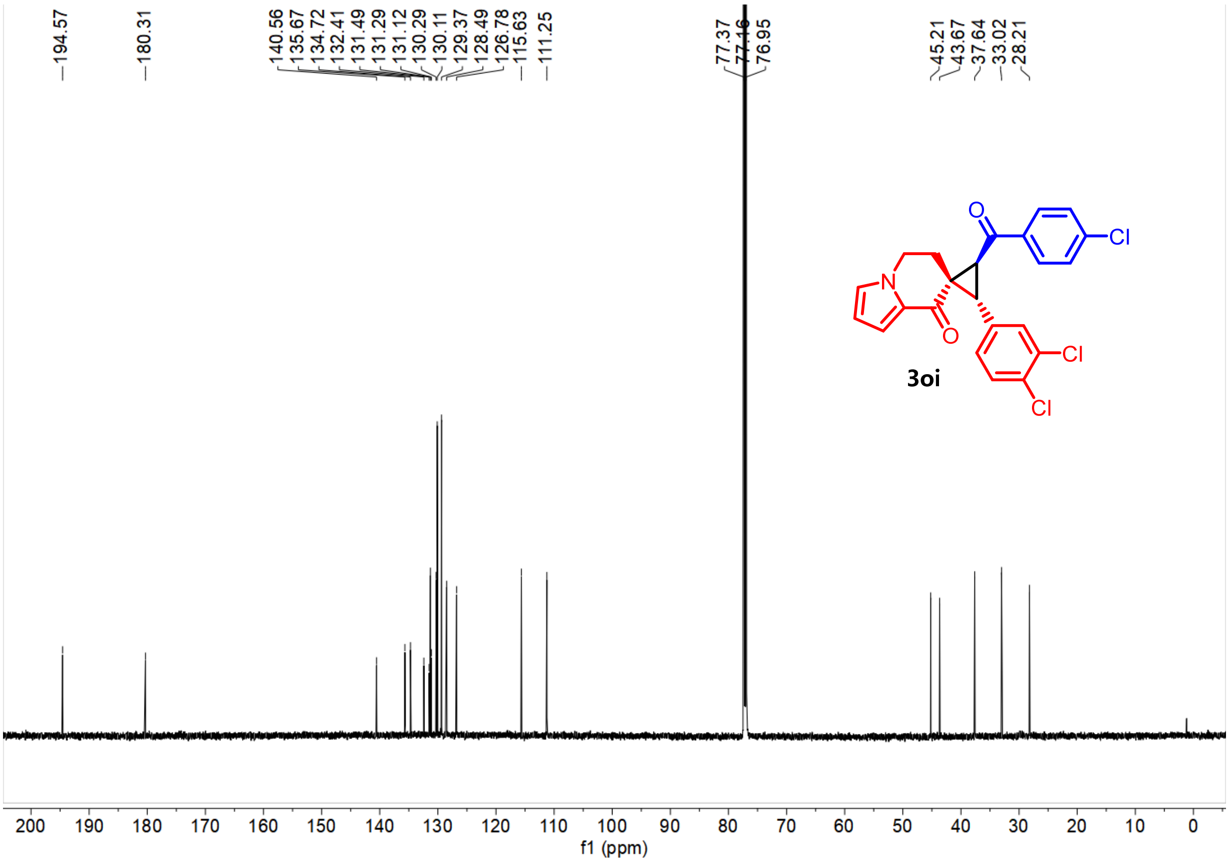


**Figure S60.** $^{13}$C-NMR of compound **3oi**

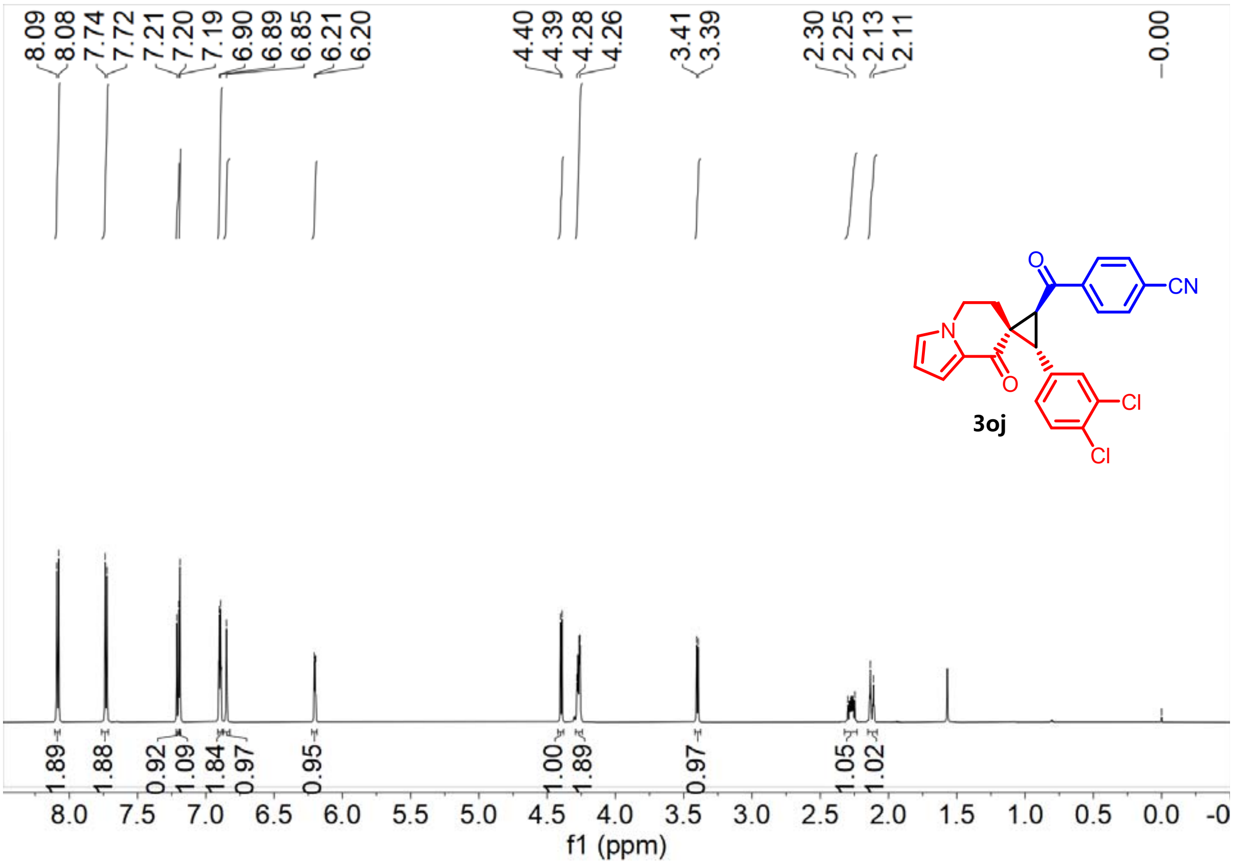


**Figure S61.** $^{1}$H-NMR of compound **3oj**

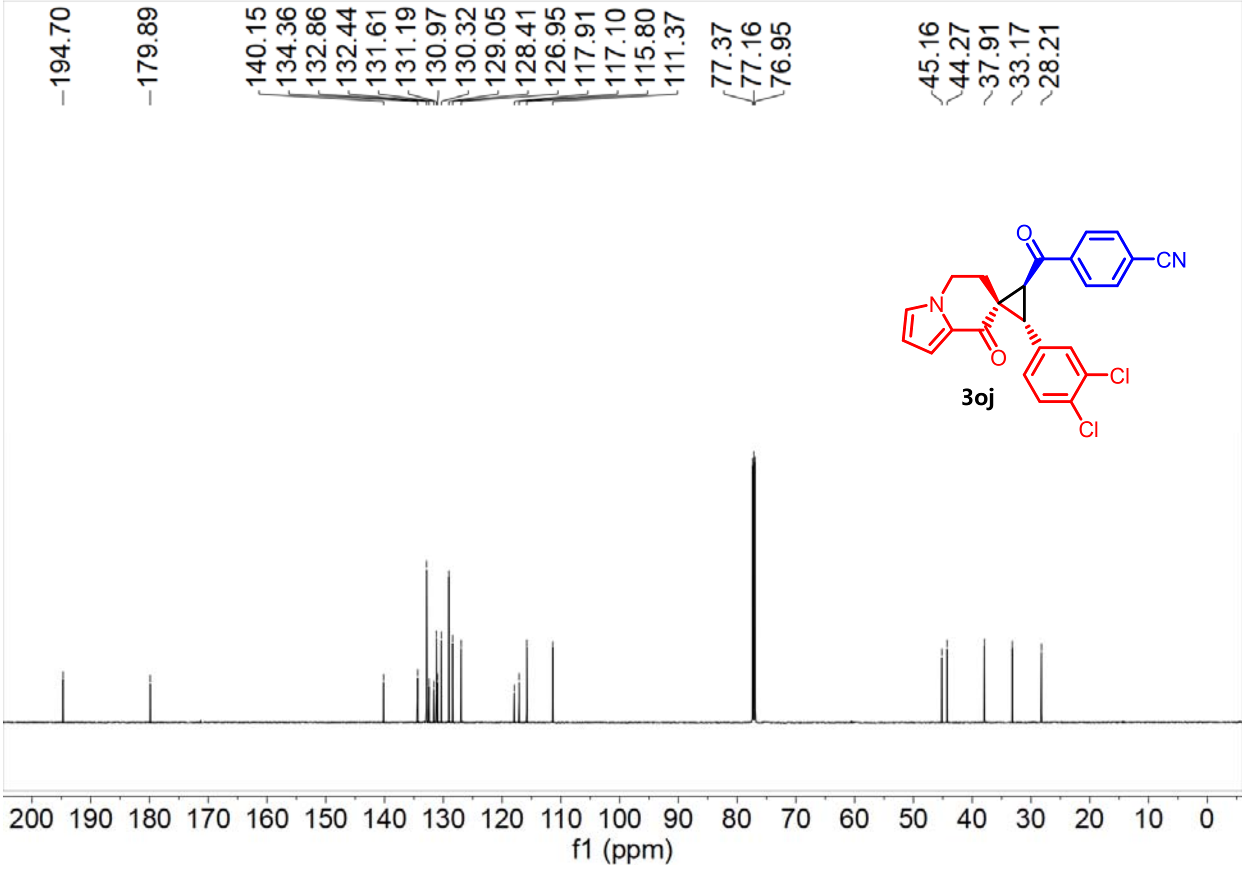


**Figure S62.** $^{13}$C-NMR of compound **3oj**

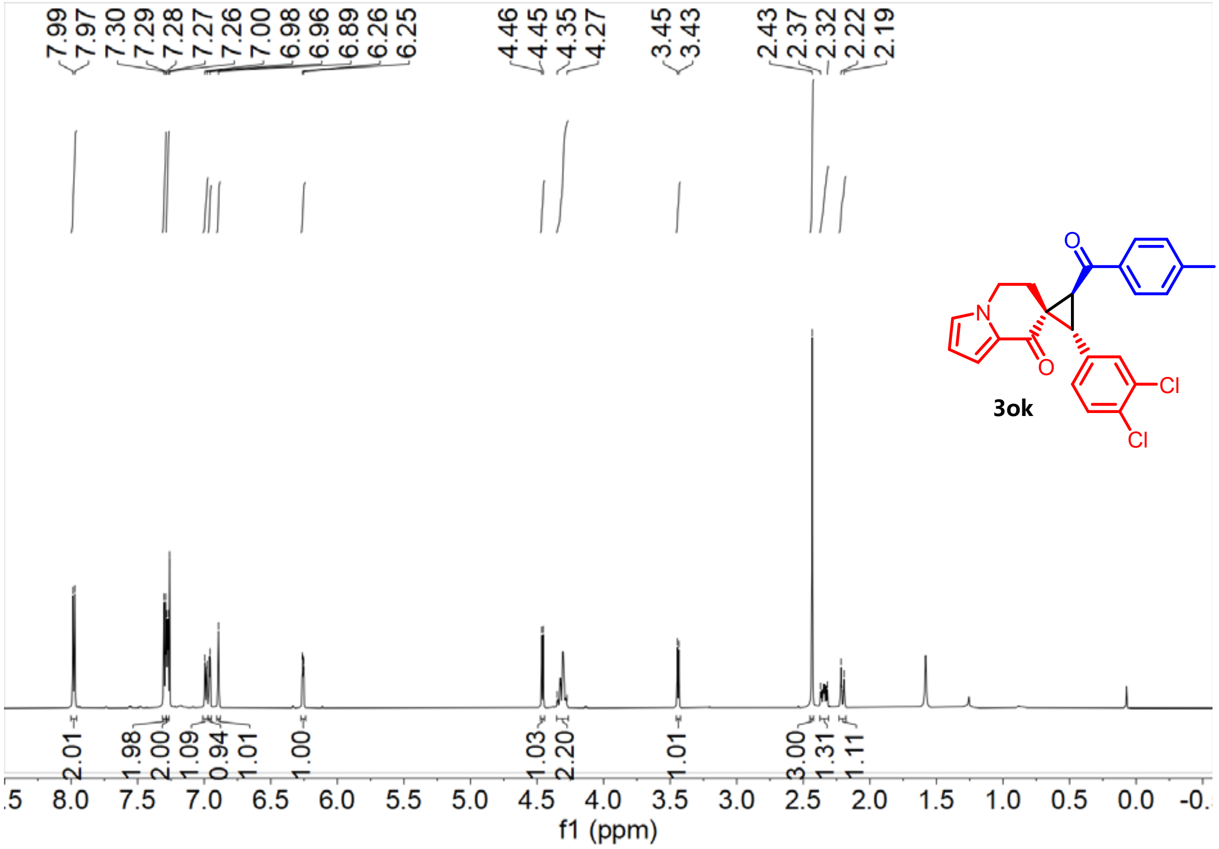


**Figure S63.** $^{1}H$-NMR of compound **3ok**

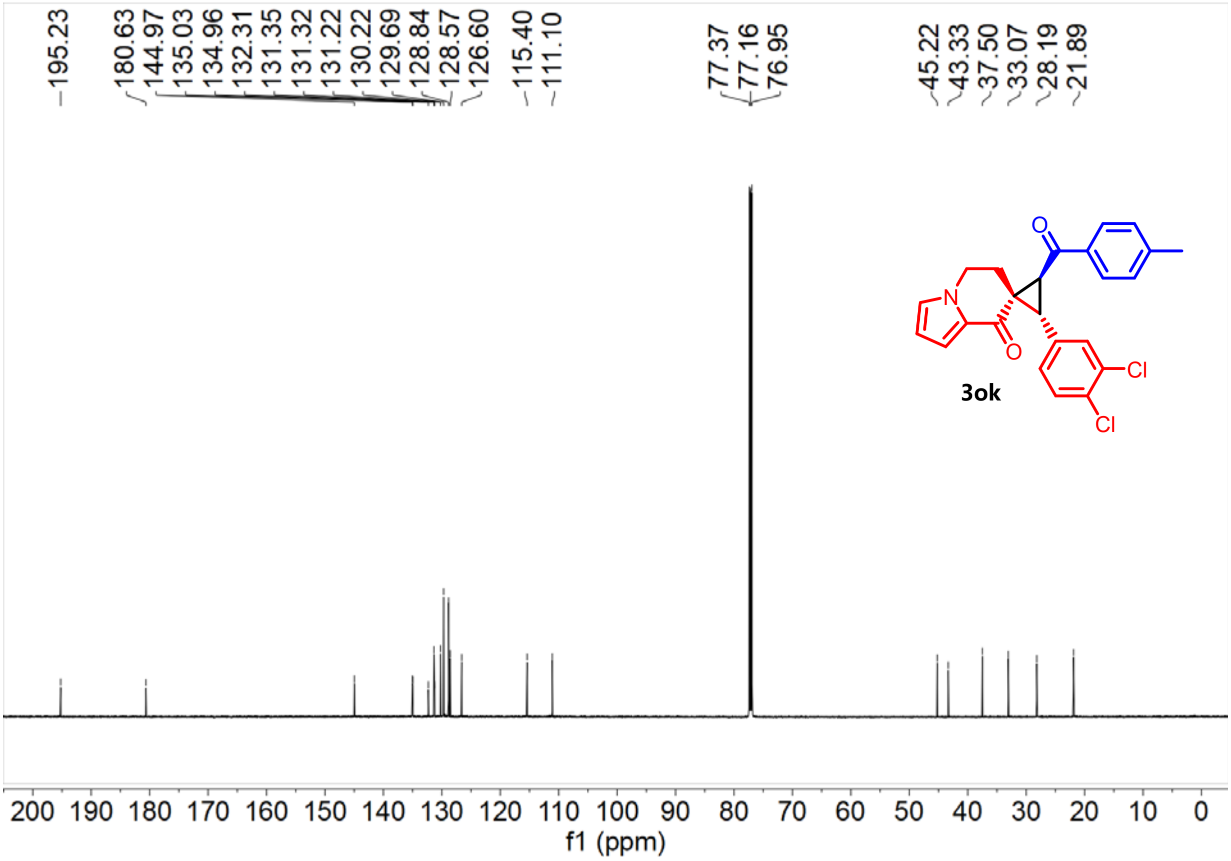


**Figure S64.** $^{13}C$-NMR of compound **3ok**

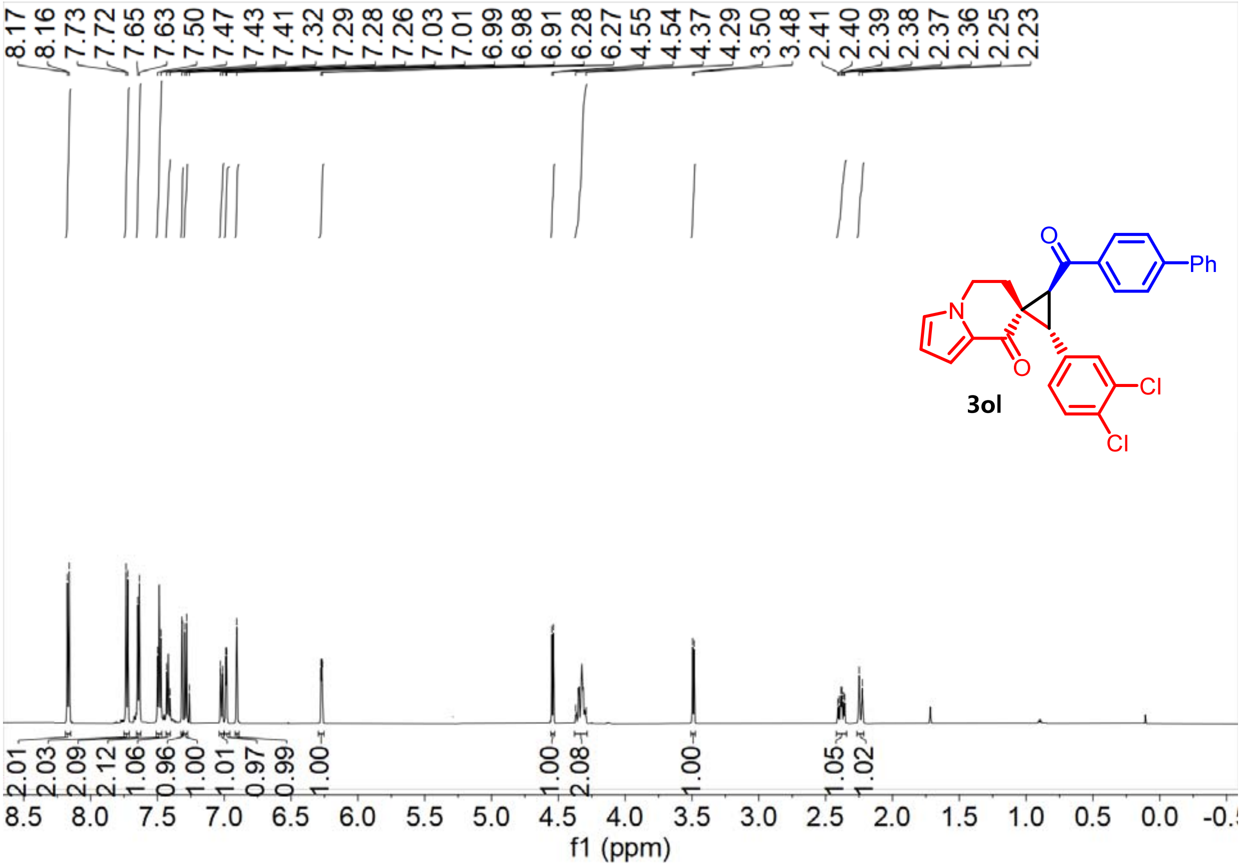

**Figure S65.** $^{1}$H-NMR of compound **3ol**

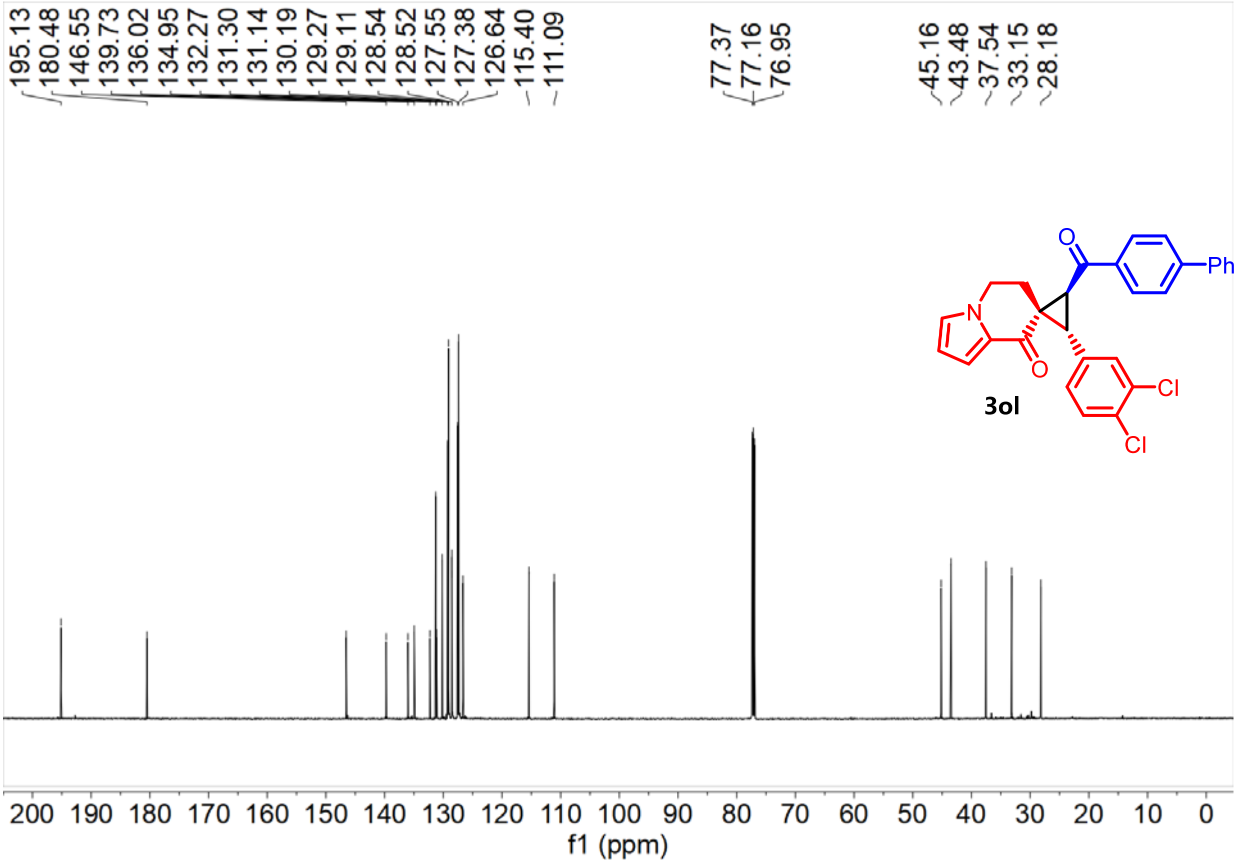

**Figure S66.** $^{13}$C-NMR of compound **3ol**

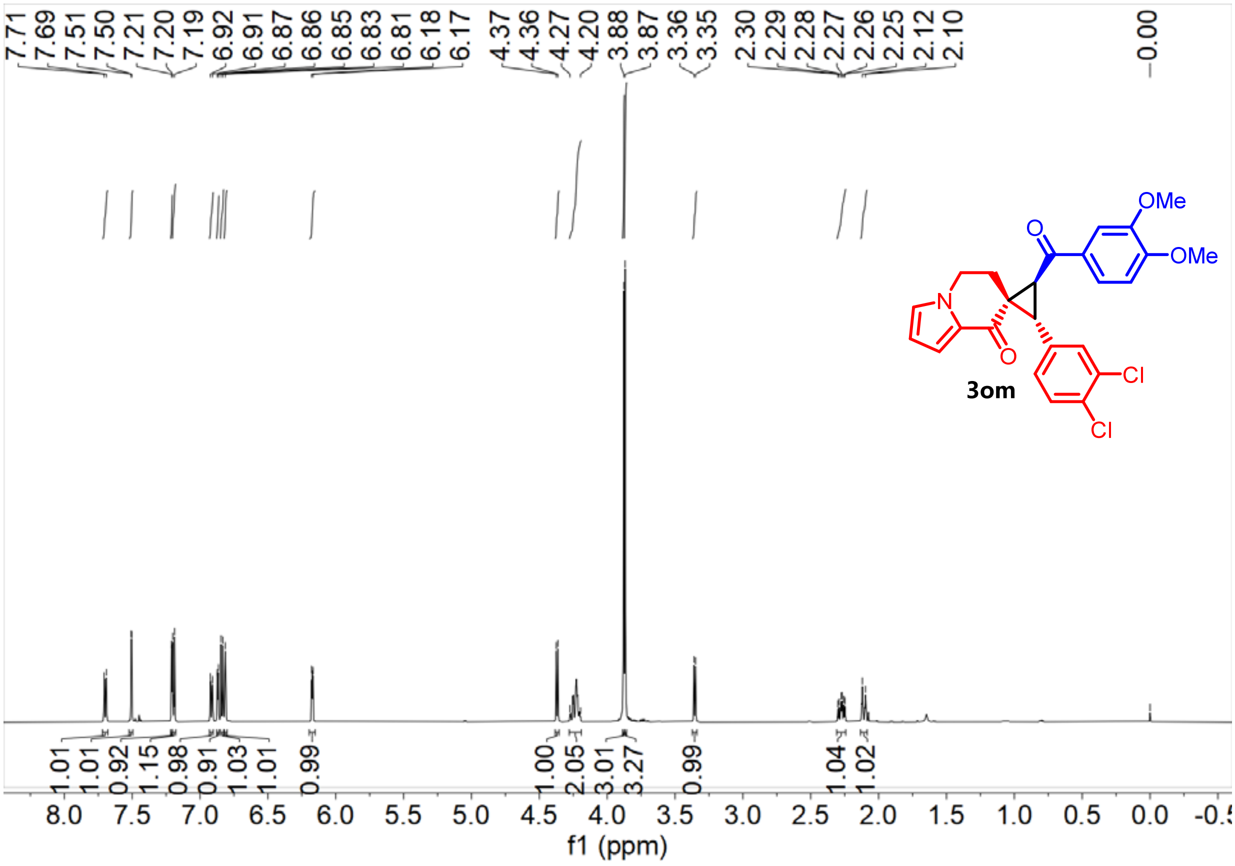

**Figure S67.** $^{1}$H-NMR of compound **3om**

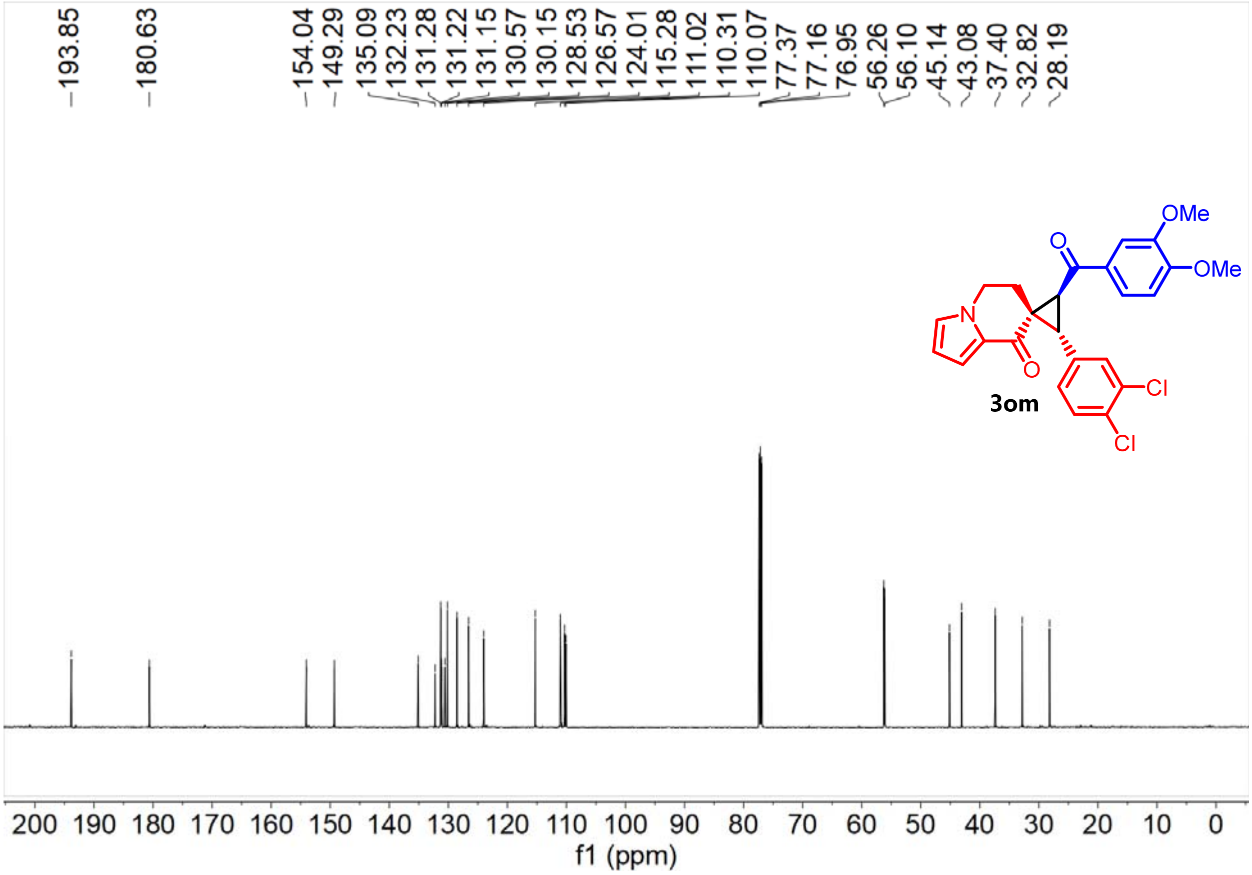

**Figure S68.** $^{13}$C-NMR of compound **3om**

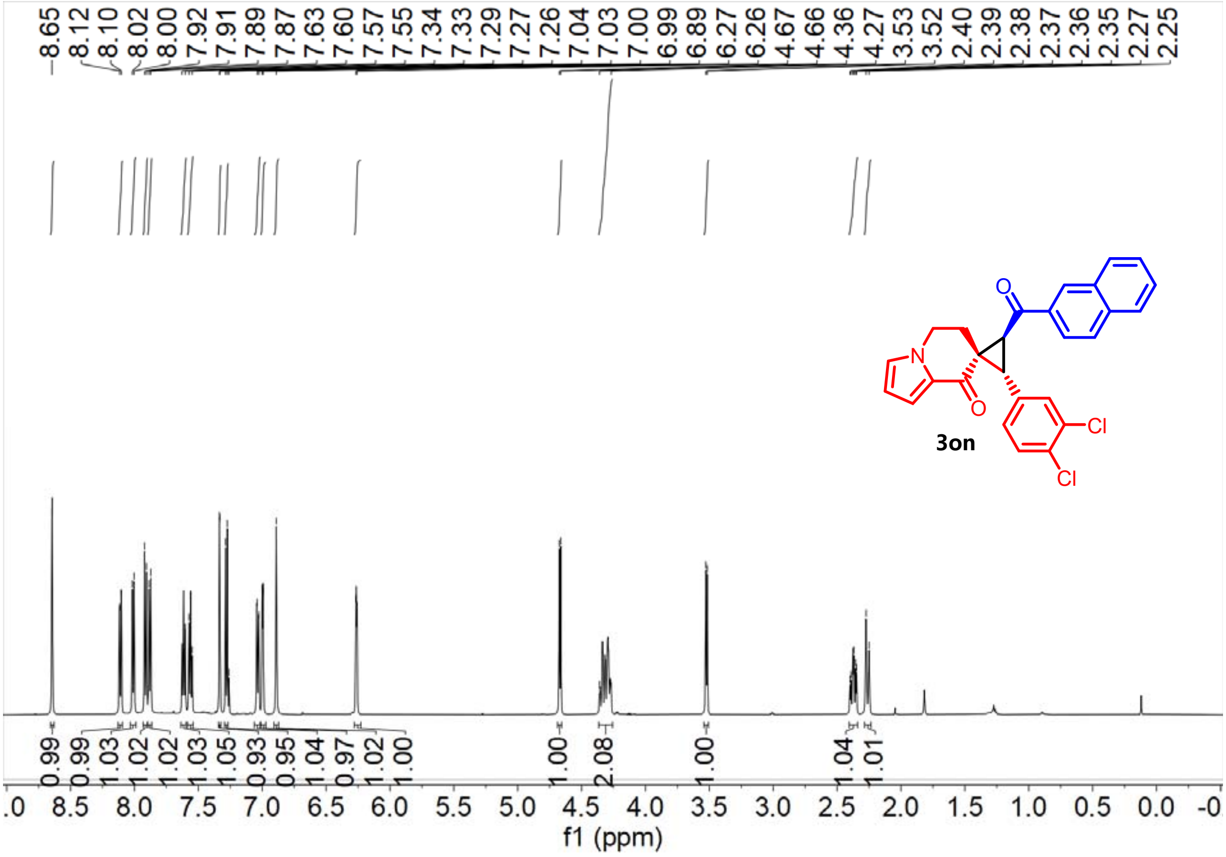


**Figure S69.** $^{1}$H-NMR of compound **3on**

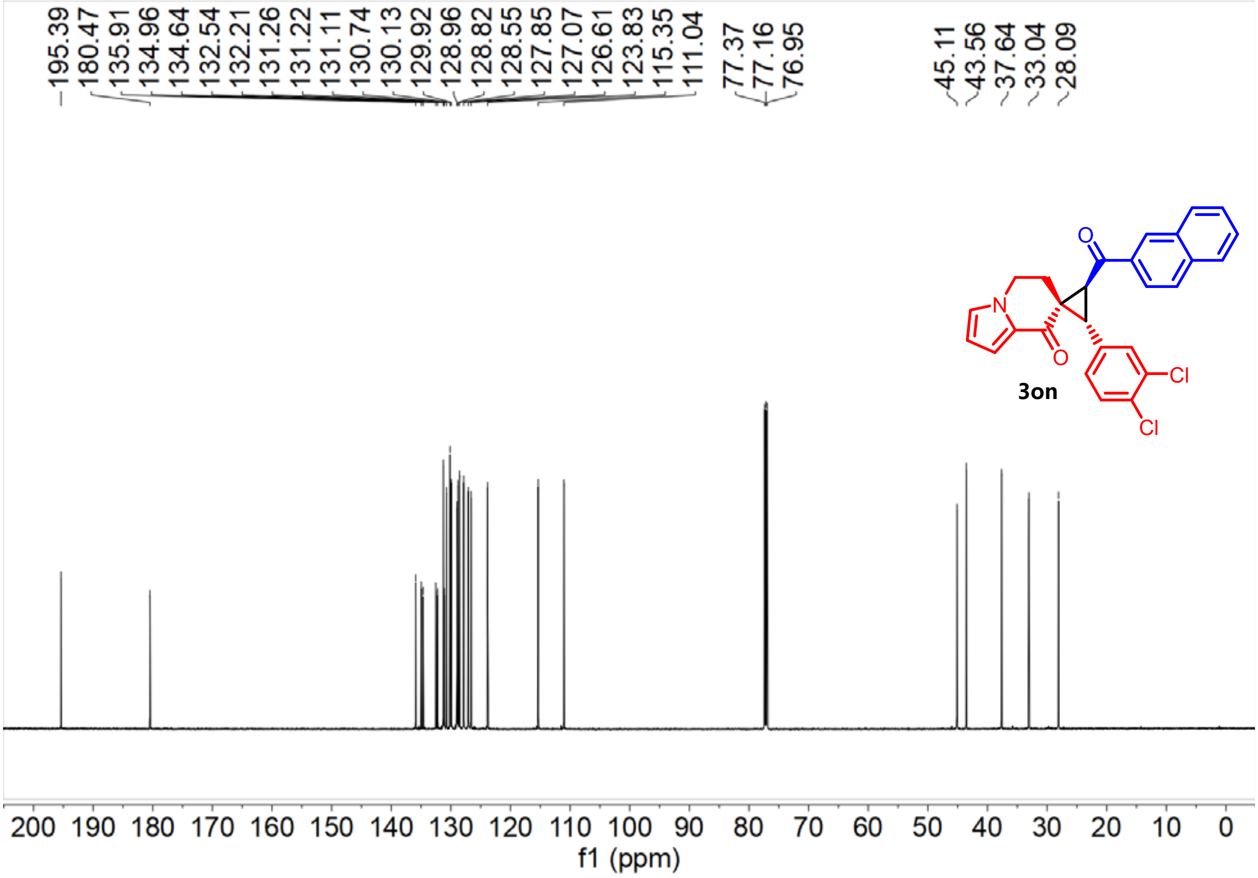


**Figure S70.** $^{13}$C-NMR of compound **3on**

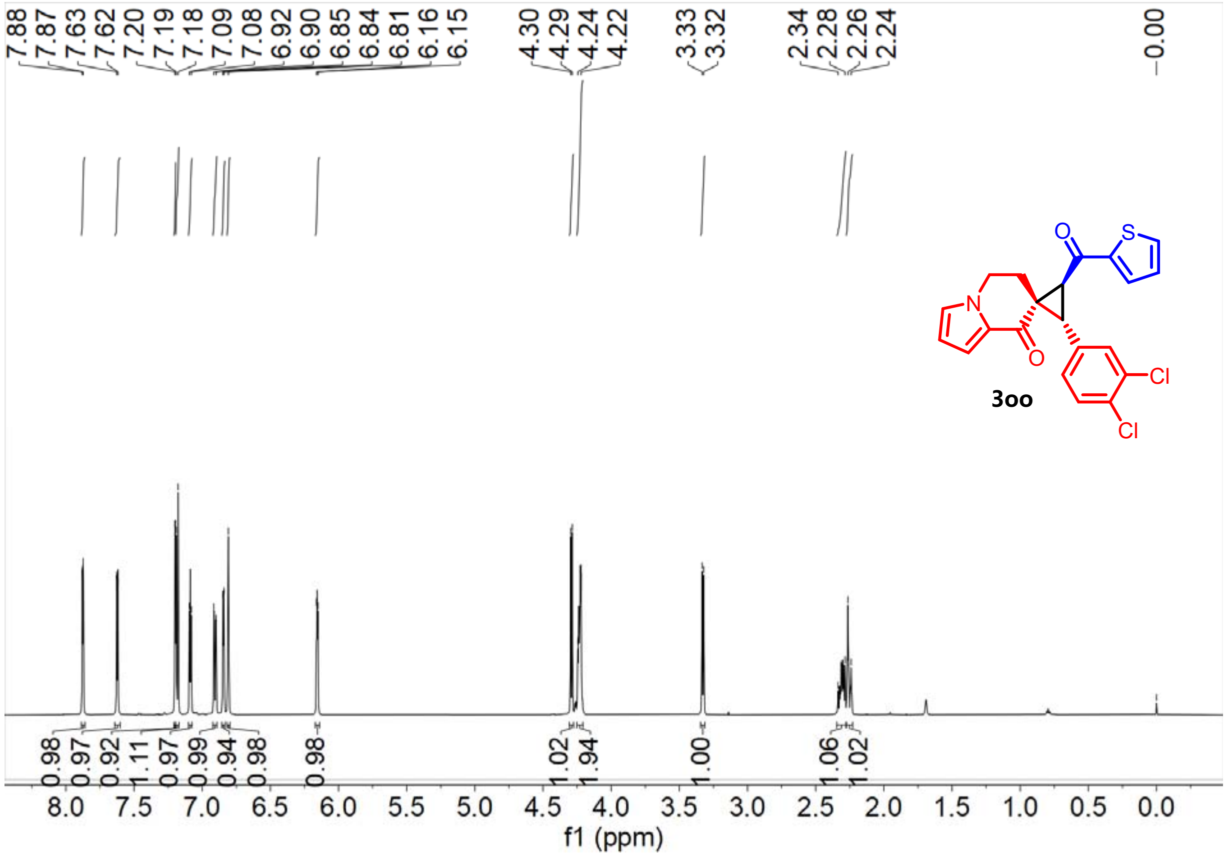


**Figure S71.** $^{1}$H-NMR of compound **3oo**

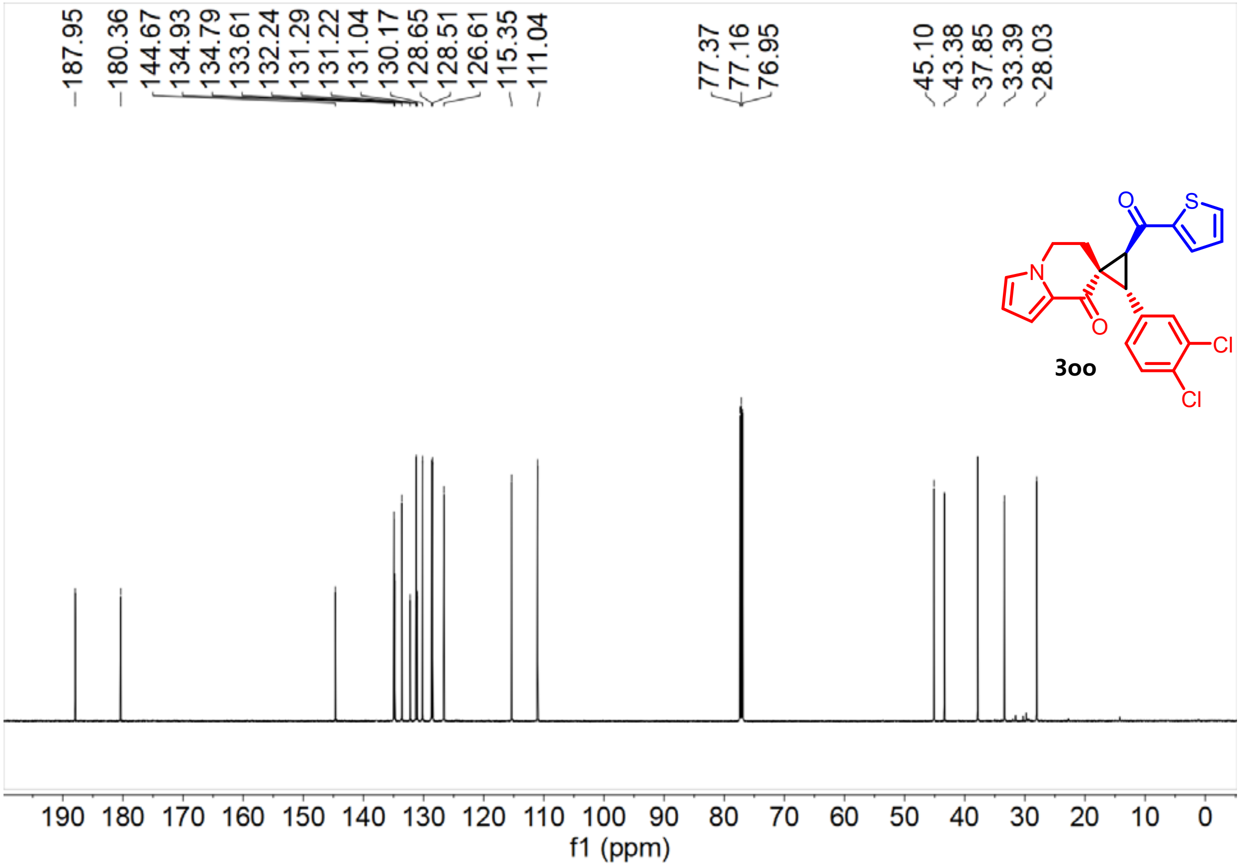


**Figure S72.** $^{13}$C-NMR of compound **3oo**

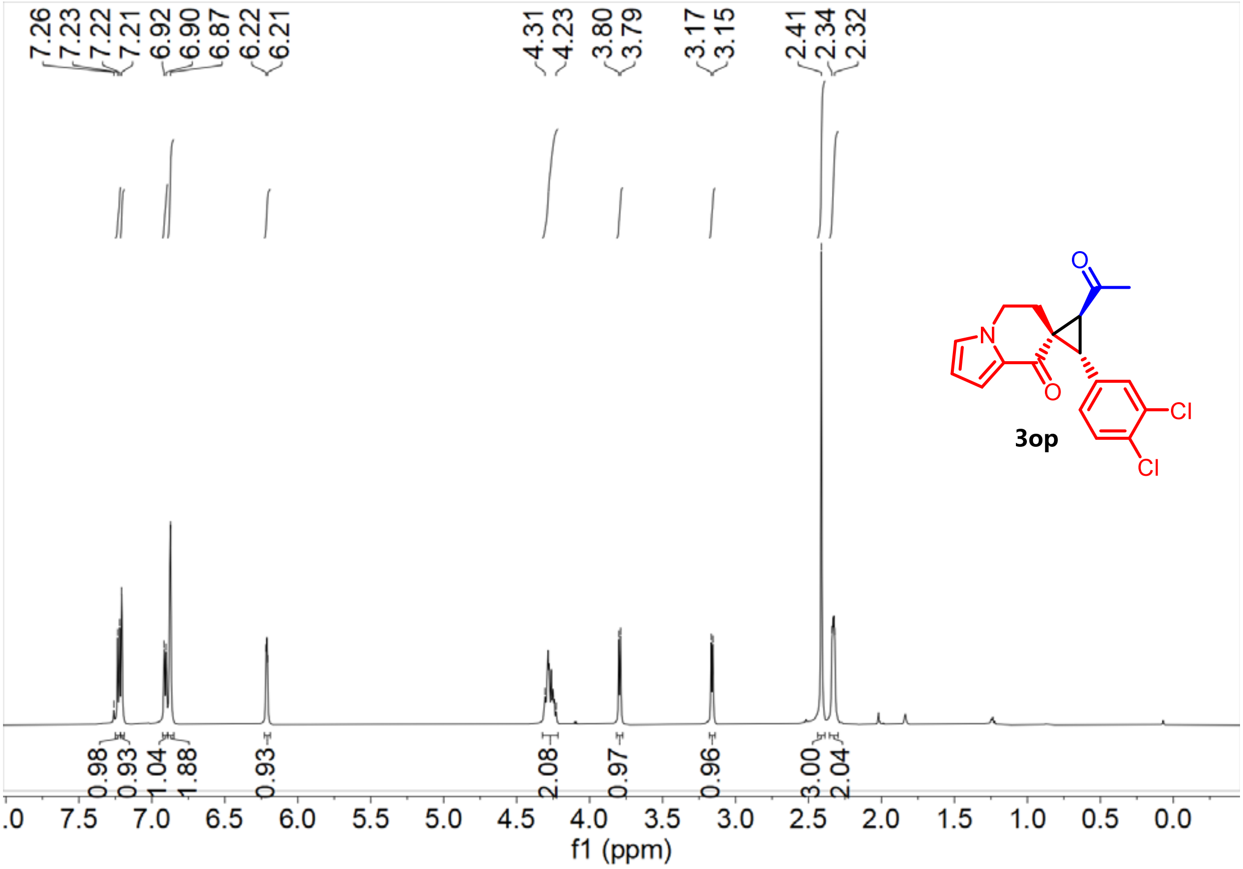


**Figure S73.** $^{1}$H-NMR of compound **3op**

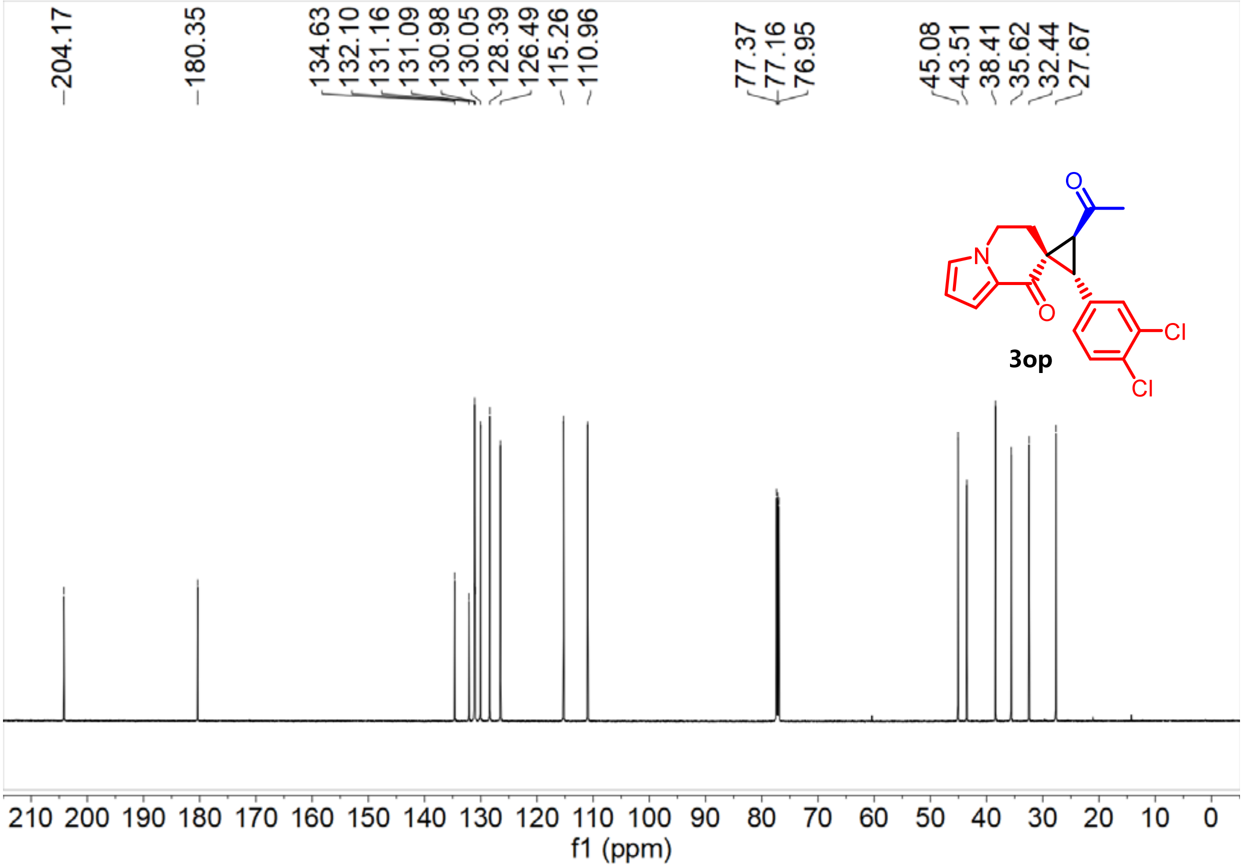


**Figure S74.** $^{13}$C-NMR of compound **3op**

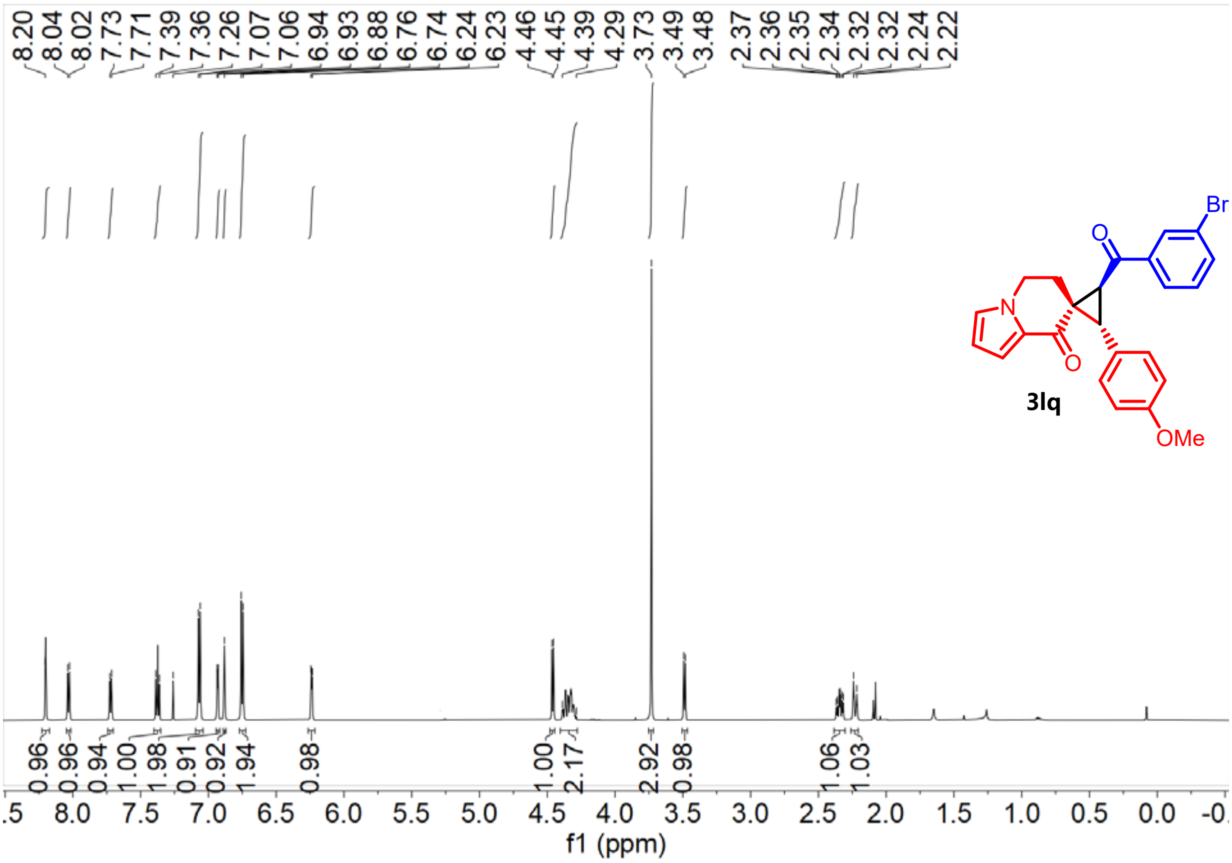


**Figure S75.** $^{1}$H-NMR of compound **3lq**

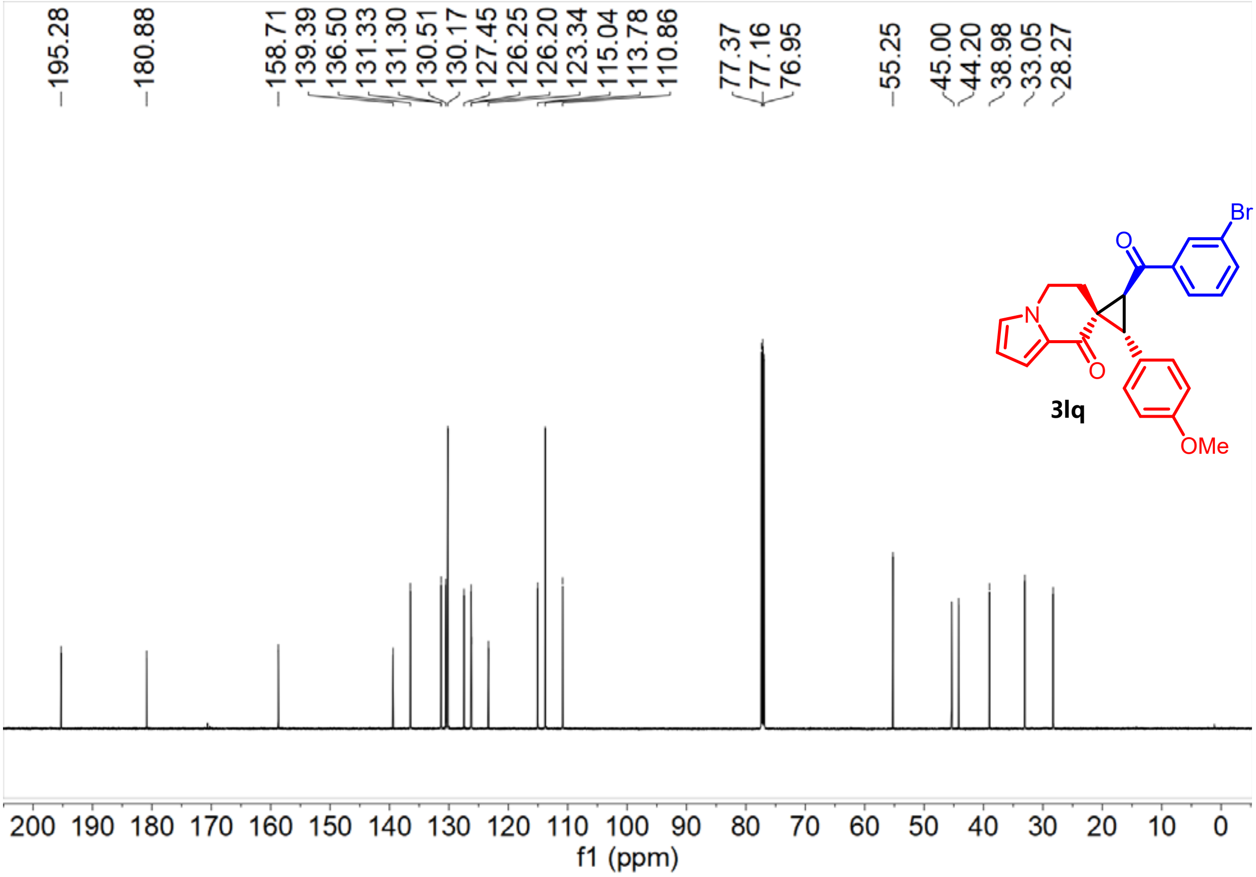


**Figure S76.** $^{13}$C-NMR of compound **3lq**

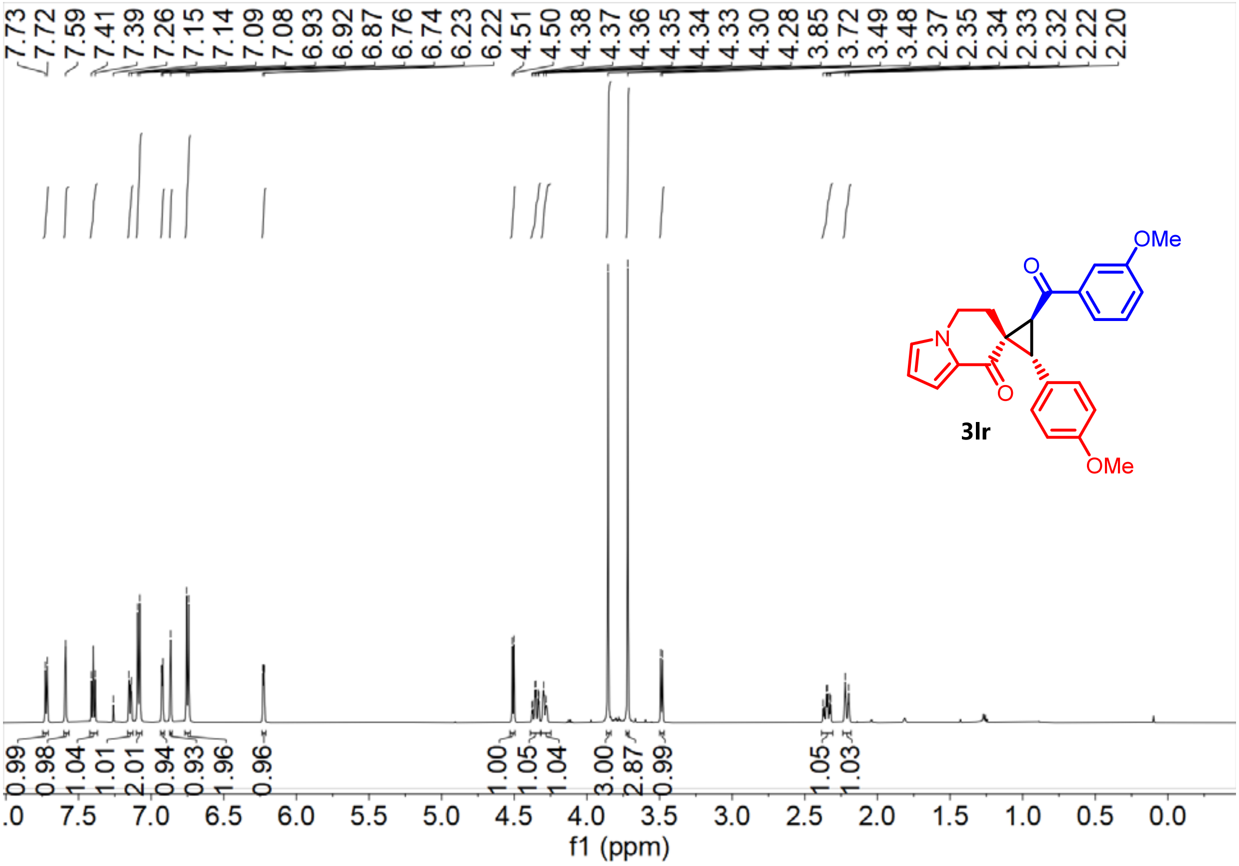


**Figure S77.** $^{1}$H-NMR of compound **3lr**

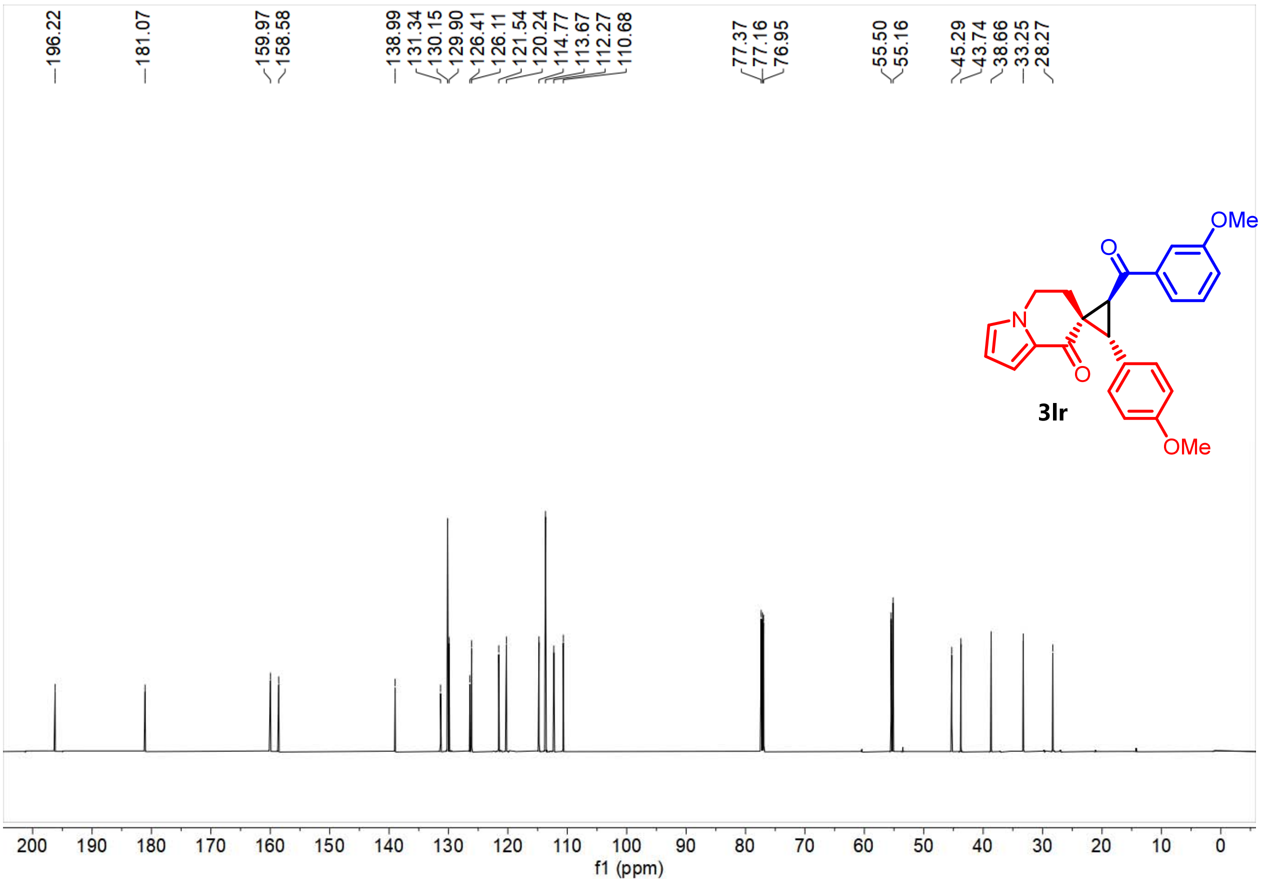


**Figure S78.** $^{13}$C-NMR of compound **3lr**

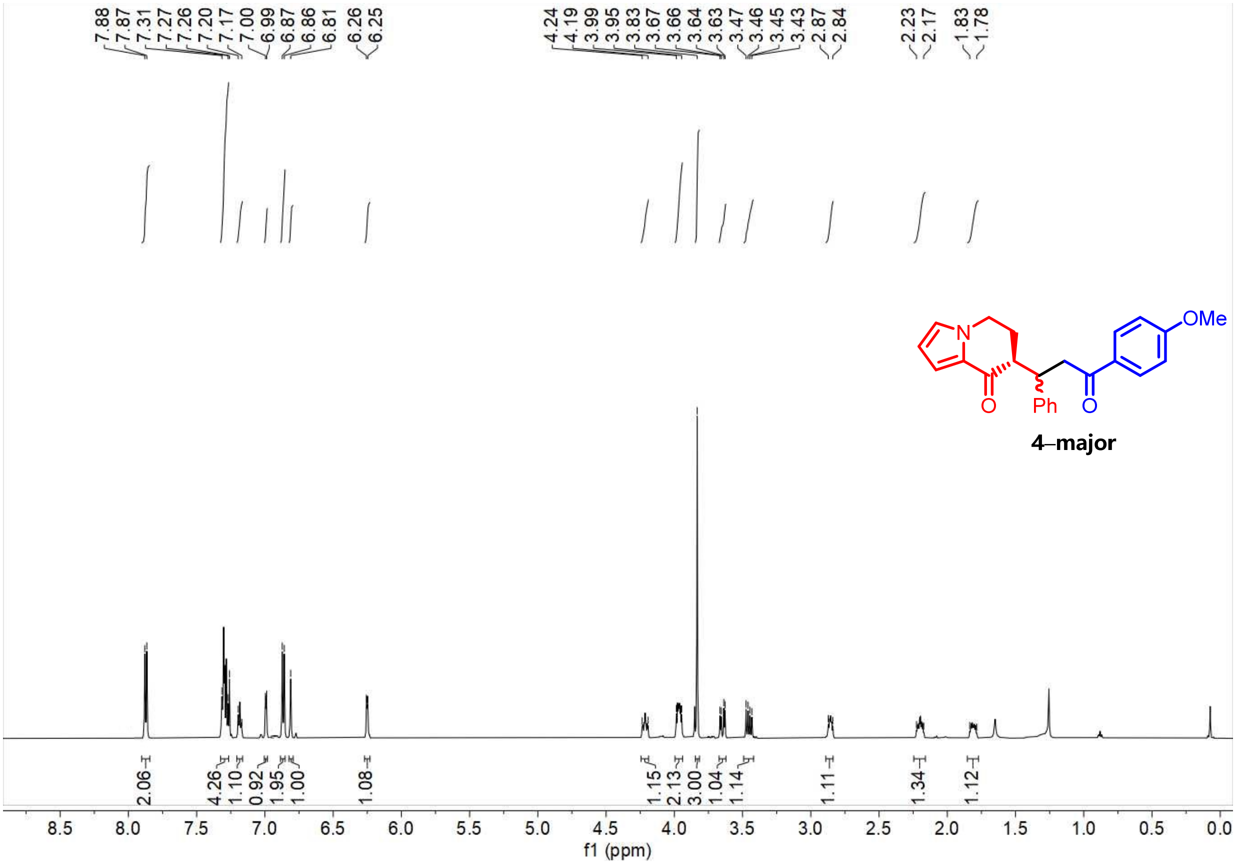


**Figure S79.** $^{1}$H-NMR of compound **4-major**

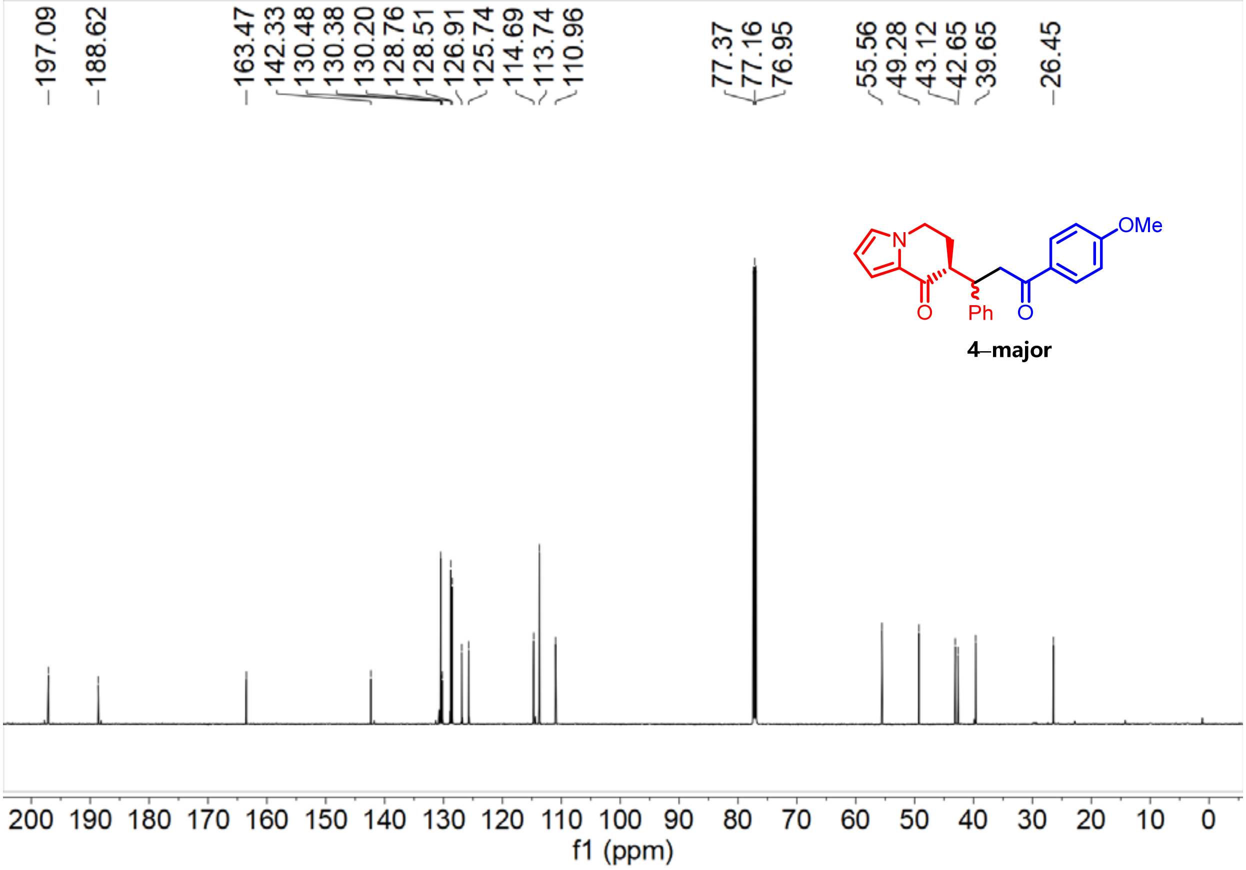


**Figure S80.** $^{13}$C-NMR of compound **4-major**

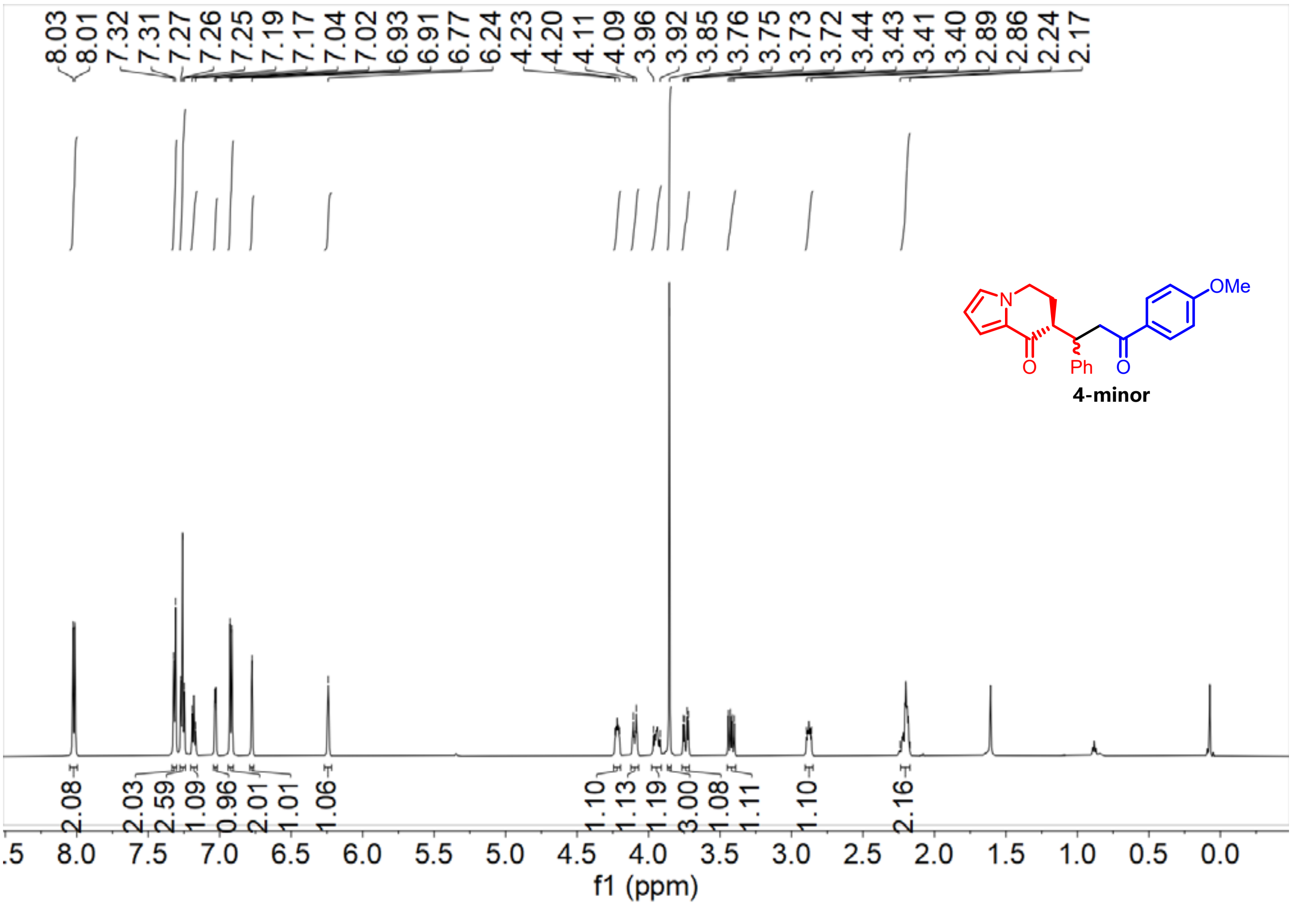


**Figure S81.** $^{1}$H-NMR of compound **4-minor**

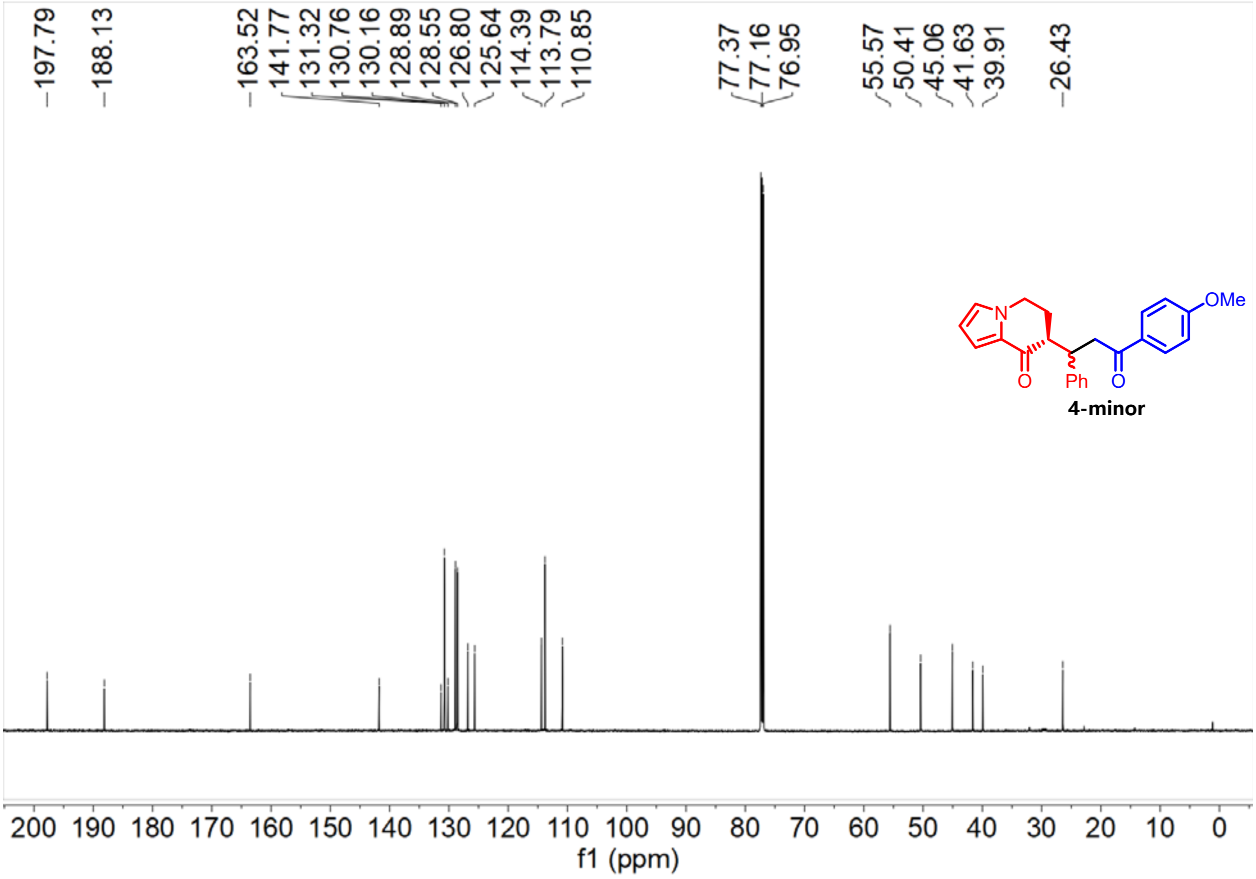


**Figure S82.** $^{13}$C-NMR of compound **4-minor**

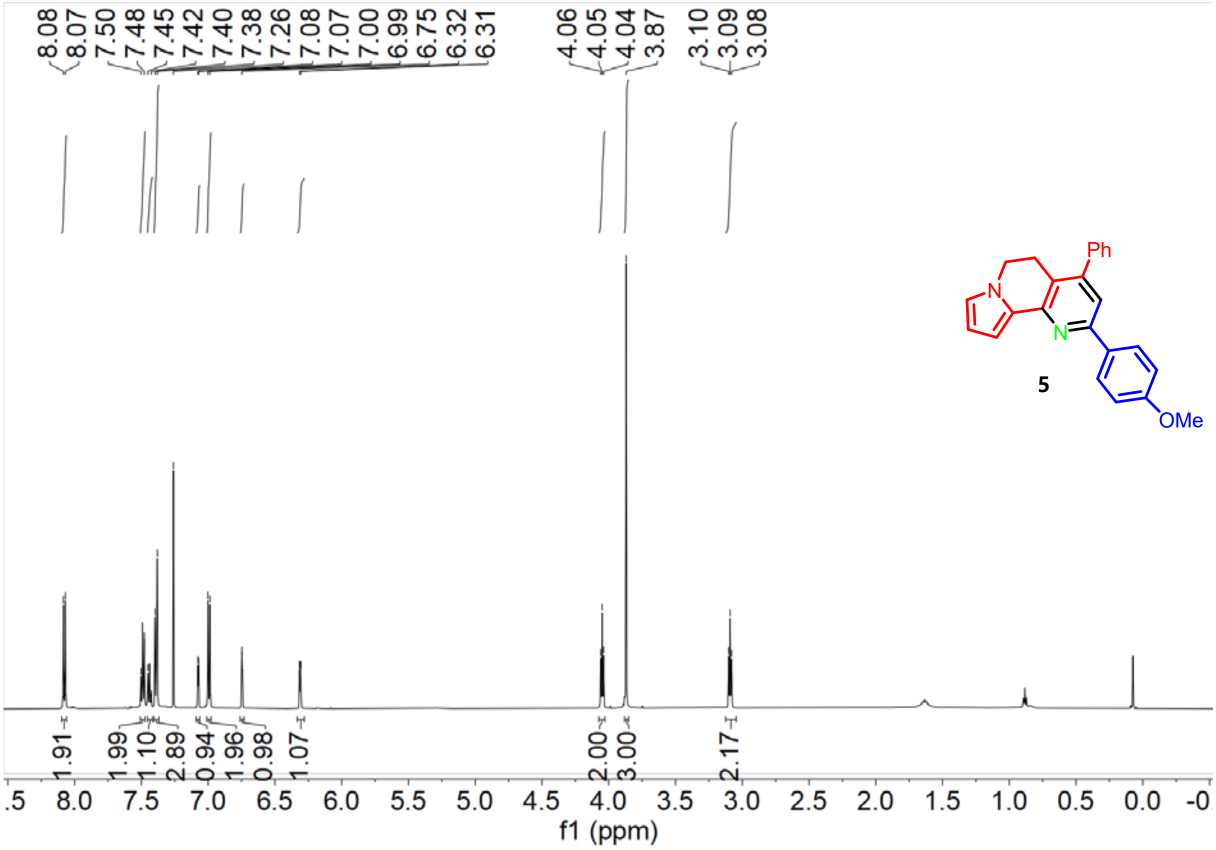

**Figure S83.** $^{1}$H-NMR of compound **5**

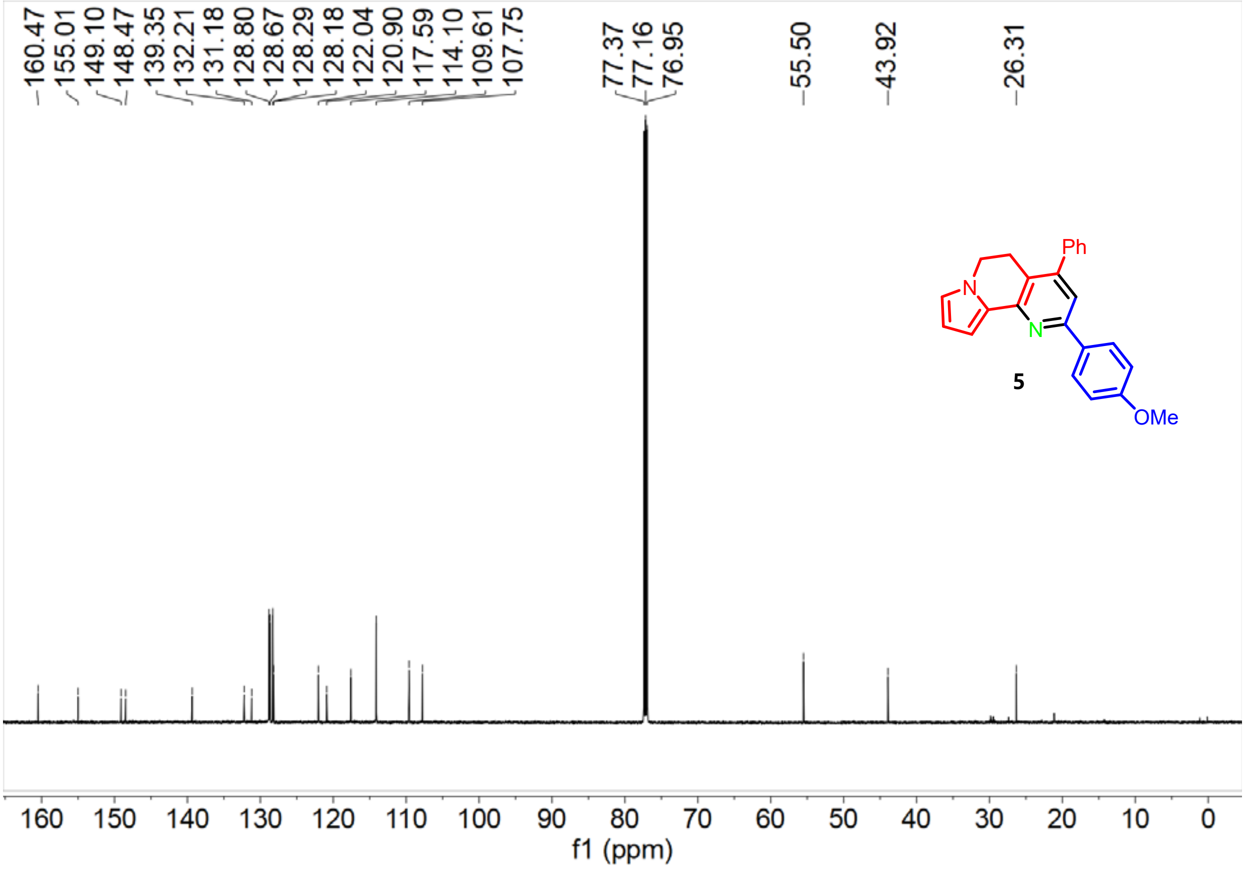

**Figure S84.** $^{13}$C-NMR of compound **5**

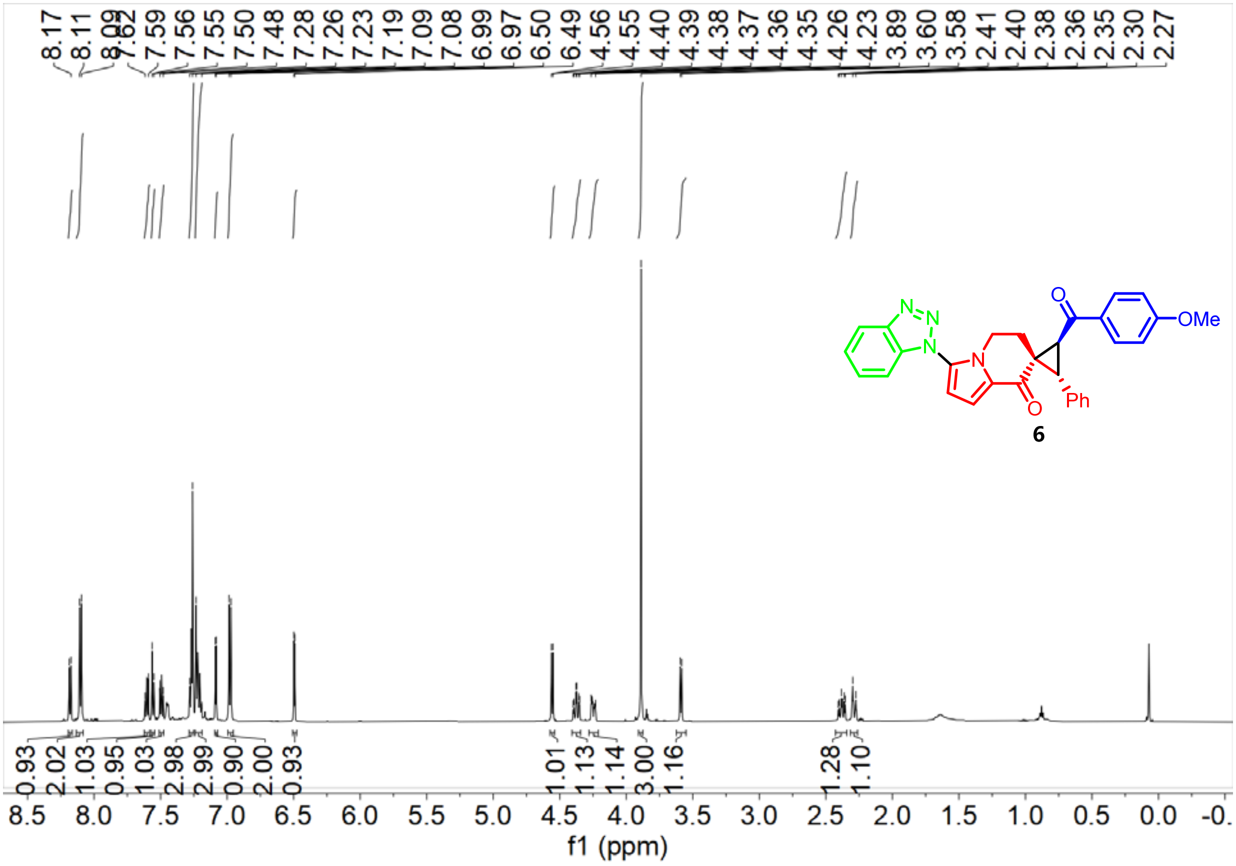

**Figure S85.** $^{1}$H-NMR of compound **6**

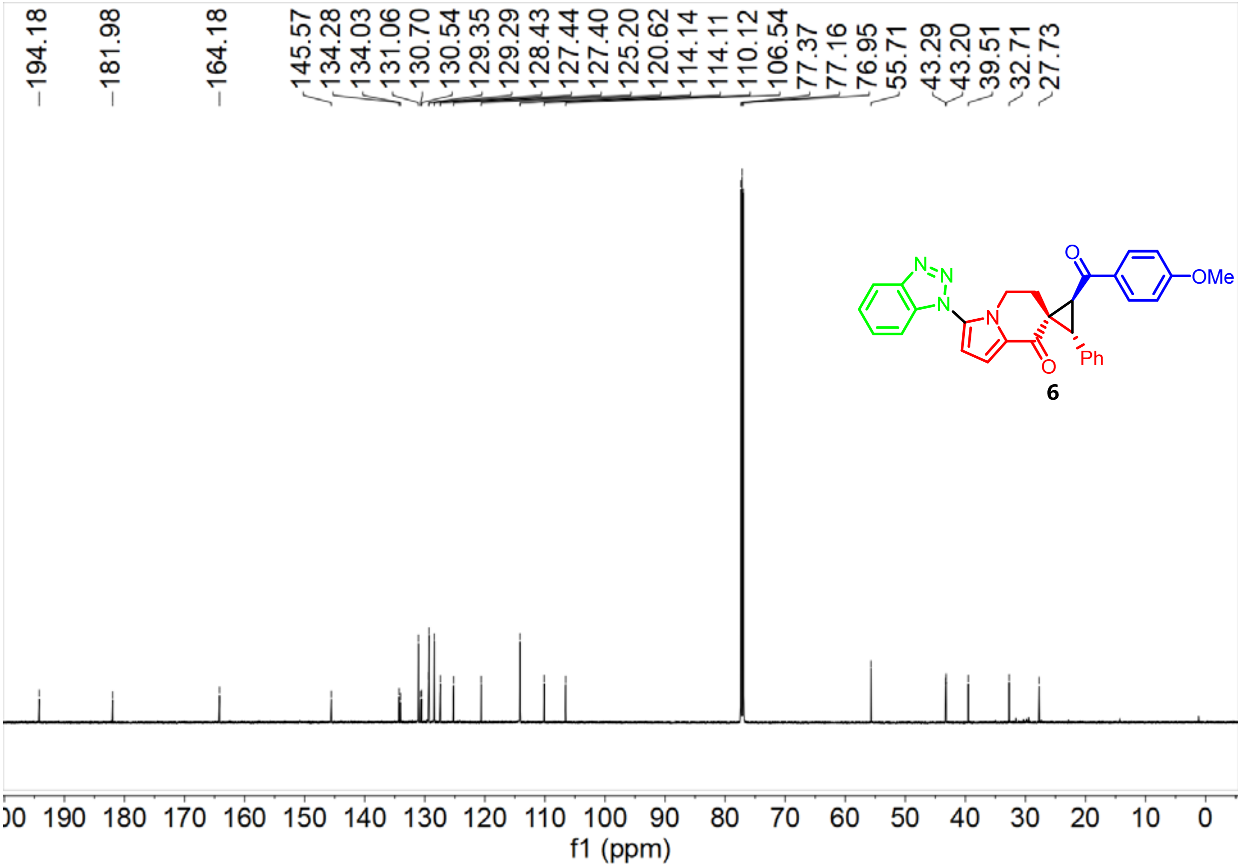

**Figure S86.** $^{13}$C-NMR of compound **6**

## VI. X-ray crystallographic data of product 3aa

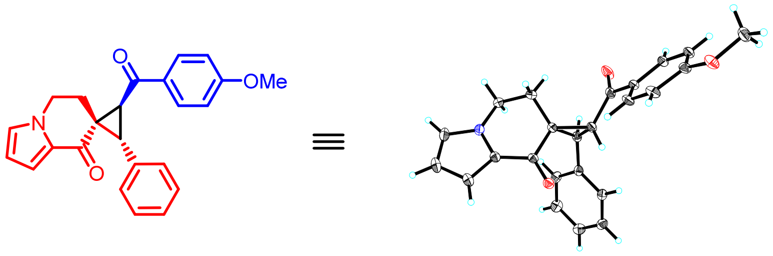


X-ray of **3aa**
CCDC: 2539636

**Figure S87.** Crystal data and structure refinement for **3aa**

Single crystal of **3aa** [$C_{24}H_{21}NO_3$] was obtained from the dichloromethane/ethyl acetate. CCDC 2539636 containing the supplementary crystallographic data can be obtained free of charge from The Cambrige Crystallographic Data Centere via www.ccdc.cam.ac.uk/data_request/cif.

| | | |
|---|---|---|
| Empirical formula | $C_{24}H_{21}NO_3$ | |
| Formula weight | 371.42 | |
| Temperature | 150(2) K | |
| Wavelength | 1.54178 Å | |
| Crystal system | Triclinic | |
| Space group | P-1 | |
| Unit cell dimensions | a = 8.2858(2) Å | α= 76.5620(10)°. |
| | b = 10.6617(2) Å | β= 86.6890(10)°. |
| | c = 11.4374(2) Å | γ = 76.8950(10)°. |
| Volume | 957.12(3) Å$^3$ | |
| Z | 2 | |
| Density (calculated) | 1.289 Mg/m$^3$ | |
| Absorption coefficient | 0.680 mm$^{-1}$ | |
| F(000) | 392 | |
| Crystal size | 0.270 x 0.230 x 0.200 mm$^3$ | |
| Theta range for data collection | 3.97 to 68.31°. | |
| Index ranges | -9<=h<=9, -12<=k<=12, -13<=l<=13 | |
| Reflections collected | 15037 | |
| Independent reflections | 3476 [R(int) = 0.0502] | |

| | |
|---|---|
| Completeness to theta = 68.31° | 99.2% |
| Absorption correction | Semi-empirical from equivalents |
| Max. and min. transmission | 0.88 and 0.75 |
| Refinement method | Full-matrix least-squares on $F^2$ |
| Data / restraints / parameters | 3476 / 0 / 254 |
| Goodness-of-fit on $F^2$ | 1.040 |
| Final R indices [I>2sigma(I)] | $R_1$ = 0.0403, $wR_2$ = 0.1008 |
| R indices (all data) | $R_1$ = 0.0448, $wR_2$ = 0.1043 |
| Largest diff. peak and hole | 0.186 and -0.228 e.Å$^{-3}$ |
